# Novel 3-aminobenzofuran derivatives as multifunctional agents for the treatment of Alzheimer’s disease

**DOI:** 10.3389/fchem.2022.882191

**Published:** 2022-08-09

**Authors:** Zaman Hasanvand, Rasoul Motahari, Hamid Nadri, Setareh Moghimi, Roham Foroumadi, Adileh Ayati, Tahmineh Akbarzadeh, Syed Nasir Abbas Bukhari, Alireza Foroumadi

**Affiliations:** ^1^ Department of Medicinal Chemistry, Faculty of Pharmacy, Tehran University of Medical Sciences, Tehran, Iran; ^2^ Department of Medicinal Chemistry, Faculty of Pharmacy and Pharmaceutical Sciences Research Center, Shahid Sadoughi University of Medical Sciences, Yazd, Iran; ^3^ Drug Design and Development Research Center, The Institute of Pharmaceutical Sciences (TIPS), Tehran University of Medical Sciences, Tehran, Iran; ^4^ Department of Pharmacology, School of Medicine, Tehran University of Medical Sciences, Tehran, Iran; ^5^ Department of Pharmaceutical Chemistry, College of Pharmacy, Jouf University, Sakaka, Saudi Arabia

**Keywords:** Alzheheimer’s disease, acetylcholinesterase, neuroprotection, docking studies, ab-aggregation, 3-amino benzofuran

## Abstract

A novel multifunctional series of 3-aminobenzofuran derivatives **5a-p** were designed and synthesized as potent inhibitors of acetylcholinesterase (AChE) and butyrylcholinesterase (BuChE). The target compounds **5a-p** were prepared *via* a three-step reaction, starting from 2-hydroxy benzonitrile. *In vitro* anti-cholinesterase activity exhibited that most of the compounds had potent acetyl- and butyrylcholinesterase inhibitory activity. In particular, compound **5f** containing 2-fluorobenzyl moiety showed the best inhibitory activity. Furthermore, this compound showed activity on self- and AChE-induced Aβ-aggregation and MTT assay against PC12 cells. The kinetic study revealed that compound **5f** showed mixed-type inhibition on AChE. Based on these results, compound **5f** can be considered as a novel multifunctional structural unit against Alzheimer’s disease.

## 1 Introduction

Alzheimer’s disease (AD) is the most prominent neurodegenerative illness of the Central Nervous System (CNS) progressed by abnormal cholinergic neurons loss, and deposition of extracellular amyloid-β and tau-proteins into plaques and neurofibrillary tangles (Scarpini et al., 2003; McKhann et al., 2011; Li et al., 2017). This debilitating disease is mainly characterized by a deterioration of memory, and changes in learning and personality (Lai et al., 2012). Despite the implications of many factors, still there are unclear points in the pathogenesis and etiology of AD (Gauthier et al., 2006; Poprac et al., 2017; Talesa, 2021).

Acetylcholine (ACh), is a cholinergic neurotransmitter and its deficiency is responsible for the cognitive decline in AD patients which has been considered a target to designing novel agent with an AChE-inhibition profile to increase the cerebral acetylcholine level (Lane et al., 2004; Marco-Contelles et al., 2006). Cholinesterase has two types involving acetylcholinesterase (AChE, EC 3.1.1.7) and butyrylcholinesterase (BuChE, EC 3.1.1.8) catalyzing the hydrolysis of ACh to choline and acetate (Mesulam and Asunction, 1987; Weinstock, 1999). Due to the evidence about the activity of BuChE in the pathogenesis of AD, the use of nonselective inhibitors targeting both BuChE and AChE would be more beneficial in AD patients compared to selective ones. Quite a few selective and reversible anti-AChE drugs (donepezil, rivastigmine, and galantamine) have been approved for the treatment of AD. However, these drugs suffer from severe side effects including vomiting, nausea, diarrhea, bradycardia, abnormal dreams, and fatigue (Smith et al., 1997; Forette et al., 1999; Schneider 2000; Cummings et al., 2014). Considering the complex pathophysiology of this disease, there is a high demand for the discovery and development of efficient new therapeutic agents with minimal side effects to effectively combat AD.

The development of multifunctional molecules which are capable of influencing a number of targets has represented considerable attention for the treatment of AD (Cavalli et al., 2008; Camps et al., 2008; León et al., 2013; Rosini et al., 2014). Previous studies revealed that the crystal structure of AChE consists of two binding sites, the peripheral anionic site (PAS) at the entrance and the catalytic active site (CAS) at the bottom of the gorge (Muñoz-Ruiz et al., 2005). It is proposed that the peripheral anionic site of AChE could play an important role in the aggregation of insoluble forms of Aβ plaques in the brain. So, inhibition of the self-assembled Aβ peptide formation would be a promising therapeutic strategy for AD treatment (Inestrosa et al., 1996; Castro and Martinez 2001). In addition, considerable evidence implicated the important role of oxidative stress in the development and progression of AD pathogenesis. Therefore, the neuroprotection approach against oxidative stress has beneficial effects on AD (Longo and Massa 2004; Greenough et al., 2013). In order to explain the pathogenesis of AD, the cholinergic hypothesis was first proposed and is relatively accepted, indicating that the decreased levels of ACh are related to the pathogenesis of AD. As the main cholinesterase (ChE), 80% of hydrolytic activity in the brain is performed by acetylcholinesterase (AChE), so inhibiting AChE which will result in increased levels of ACh in the brain has been considered as a good strategy to treat AD. While inhibition of BuChE might be considered a promising strategy for the treatment of AD, the development of AChE inhibitors is still the most important strategy to treat AD.

**FIGURE 1:**
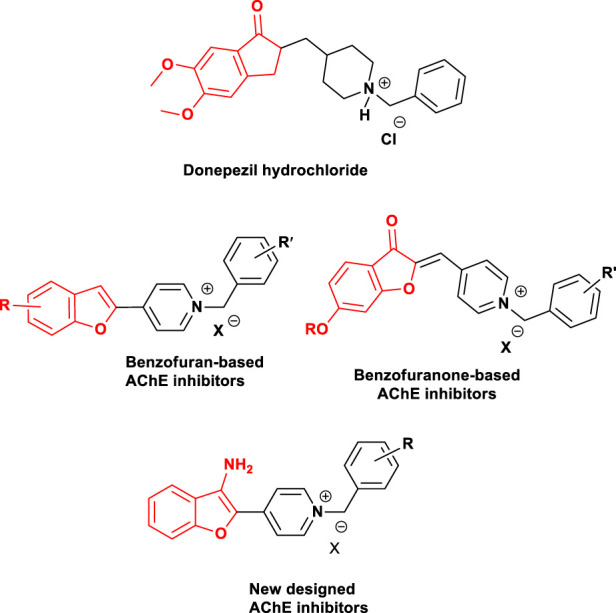
Structures of donepezil hydrochloride-based cholinesterase inhibitors, benzofuran-based compounds reported as anticholinesterase inhibitors, and newly designed benzofuran-based derivatives (Baharloo et al., 2015).

Benzofuran derivatives have attracted considerable attention in numerous natural products and therapeutic agents due to their interesting biological and pharmacological properties, including anti-Alzheimer’s, anti-inflammatory, analgesic, anti-hyperlipidemic, antiviral, antimicrobial, anti-inflammatory, and antitumor (Alper-Hayta et al., 2008; Choi et al., 2011; Al-Qirim et al., 2012; Rizzo et al., 2013; Nevagi et al., 2015). Synthesis and evaluation of different benzofuran derivatives have been considered by our research group for many years (Pouramiri et al., 2016; Mehrabi et al., 2017). In terms of the AD research, we intend to design and develop multifunctional small molecules, based on this valuable pharmacophore which can be regarded as a mimic of the indanone part of donepezil (Choi et al., 2004). The Aβ antiaggregants and site interactions with Aβ were recognized in the literature. Substitution of the C-2 position of benzofuran was done to increase the inhibitory activity towards AChE (Goyal et al., 2018). Moreover, many reports in the literature with substitution at the C-3 position of benzofuran were done to evaluate their activity as anti-Alzheimer’s agents exhibiting increased inhibitory activity towards Aβ (Howlett et al., 1999; Byun et al., 2008). Donepezil is an FDA-approved drug showing dual-binding site interactions. Docking studies indicated the interactions of *N*-benzyl piperidine residue with the catalytic active site (CAS) and interactions of hydrophobic aromatic part of the molecule with the peripheral anionic site (PAS) of AChE (Koellner et al., 2000; Saxena et al., 2003; Kapková et al., 2006). Up to now, quite a few numbers of aromatic rings have been reported as PAS binding scaffolds. In the light of the aforementioned and following our previous attempts (Nadri et al., 2010; Baharloo et al., 2015), in this study, we describe the design, synthesis, and biological evaluation of a novel series of 3-aminobenzofuran-based derivatives as multifunctional ligands targeting acetylcholinesterase, butyrycholinesterase, and β-Amyloid aggregation (Figure 1).

## 2 Results and discussion

### 2.1 Chemistry

**SCHEME 1:**
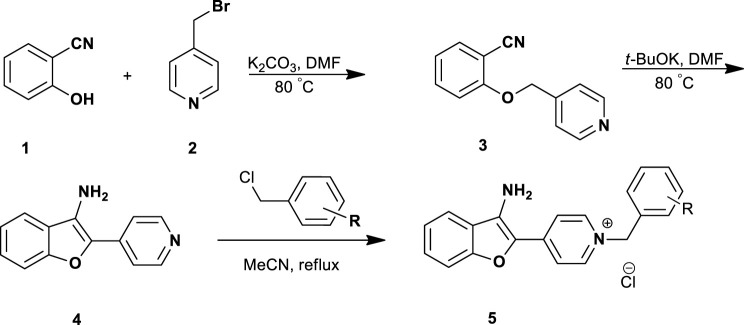
Synthesis of *N*-benzyl-4-(3-aminobenzofuran-2-yl)pyridinium chloride compounds **5a-p**.

The 3-aminobenzofuran derivatives **5a-p** were prepared according to Scheme 1. The pathway was started from the reaction of commercially available 2-hydroxybenzonitrile **1** with 4-(bromomethyl) pyridine **2** in the presence of K_2_CO_3_, afforded 2-(pyridin-4-ylmethoxy)benzonitrile **3**. The cyclization reaction of compound **3** in the presence of *t*-BuOK in DMF at 80°C afforded 4-(3-aminobenzofuran-2-yl) pyridine **4** (Chen et al., 2013; Loidreau et al., 2013). Accordingly, the final compounds **5a-p** were prepared by the reaction of compound **4** with different benzyl chloride derivatives in dry acetonitrile under reflux conditions (1–6 h). After the completion of the reaction, the final products were collected by filtration and washed with *n*-hexane to afford target compounds in good yields ranging from 56% to 74%.

### 2.2 Biological screenings

#### 2.2.1 Cholinesterase inhibition

The in-vitro cholinesterase inhibitory activity of synthesized 3-aminobenzofuran-based compounds **5a-p** was evaluated by using the Ellman’s method (Ellman et al., 1961). This method is based on the reaction of acetylthiocholine with 5,5′´-dithio-bis-(2-nitrobenzoic) acid (DTNB) to yield the colored product. The obtained IC_50_ values are summarized in Table 1 and compared with donepezil as the standard drug. Accordingly, almost all 3-aminobenzofuran derivatives exhibited moderate to good inhibitory activity with IC_50_s ranging from 0.64 to 81.06 μM. Among the tested compounds, analog **5f** was the most effective inhibitor against AChE and BuChE, while compound **5e** containing 3-methoxybenzyl moiety showed the least inhibitory activity on both. The nonpolar group (OCH_3_) has decreased the AChE inhibitory potency due to increased lipophilic characteristics.

**TABLE 1:** Inhibitory activity of the target compounds **5a-p** against AChE and BuChE.

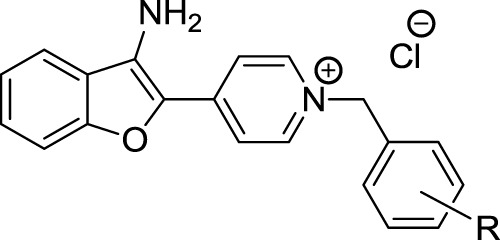			
Compounds	R	IC_50_ (μM)^a^ AChE	IC_50_ (μM) BuChE
**5a**	H	0.81 ± 0.02	0.82 ± 0.07
**5b**	2-Me	3.29 ± 0.01	17.35 ± 2.45
**5c**	3-Me	3.32 ± 0.04	12.89 ± 3.56
**5d**	4-Me	29.31 ± 0.82	25.33 ± 1.91
**5e**	3-OCH_3_	81.06 ± 1.61	>100
**5f**	**2-F**	**0.64 ± 0.04**	**0.55 ± 0.07**
**5g**	3-F	1.68 ± 0.02	2.89 ± 0.42
**5h**	4-F	0.88 ± 0.04	5.05 ± 0.73
**5i**	2-Cl	0.70 ± 0.01	0.99 ± 0.05
**5j**	3-Cl	2.99 ± 0.03	18.77 ± 1.11
**5k**	4-Cl	16.60 ± 0.52	31.18 ± 2.56
**5l**	2-Br	1.34 ± 0.01	10.54 ± 0.19
**5m**	3-Br	4.47 ± 0.02	13.95 ± 1.20
**5n**	4-Br	15.84 ± 0.91	22.55 ± 3.33
**5o**	2-NO_2_	7.84 ± 0.05	28.63 ± 0.13
**5p**	4-NO_2_	20.55 ± 0.8	28.26 ± 0.48
Donepezil	—	0.016 ± 0.001	3.99 ± 0.27

a The concentration (mean ± SEM of three experiments) required for 50% inhibition. The bold value was the most active compound.

Based on the structure-activity relationship (SAR) study, the potency of compounds was influenced by the type and the position of the substitutions. The unsubstituted derivative **5a** showed potent activity against the AChE enzyme with IC_50_ = 0.81 μM, however, the attachment of electron-donating groups such as methyl and methoxy significantly reduced the inhibitory activities of compounds. Those compounds containing electron-withdrawing groups (fluoro, chloro, and bromo) showed better inhibitory potencies than the compounds containing electron-donating groups **5b-5e**.

The SAR analysis indicated that fluorobenzyl-containing compounds **5f-5h** had promising enzymatic inhibitory activity (IC_50_s ranging from 0.64 to 1.68 μM) and the presence of fluoro substituent at *ortho* and *para* positions of the benzyl moiety led to better anti-AChE activity than *meta* substituted one **5g**. In the chloro and bromo substituted derivatives, compounds containing *ortho* and *metha* substitutions (**5i, 5j**, **5l,** and **5m**) showed moderate inhibitory activities, however, the attachment of these substitutions to *para* position dramatically decreased inhibitory activity of compounds (**5k** and **5n**). Among the electron-withdrawing-containing compounds, nitro-containing derivatives showed weak inhibitory potencies, and the compound with 2-nitrobenzyl group **5o** showed better activity than that of 4-nitrobenzyl derivative **5p**.

The obtained results showed that compounds bearing fluorobenzyl moiety were more effective than other benzyl derivatives. In addition, *ortho* position was more favorable than *meta*- or *para*- substituted analogs. All compounds except **5e** showed BuChE inhibition. By comparing the IC_50_ values against AChE and BuChE, it could be concluded that nearly the same trend was observed. The potent inhibitory activity toward BuChE was observed in **5f**. This compound showed good inhibition with an IC_50_ value of 0.55 μΜ. The movement of substituents involving fluorine, chlorine, bromine, and methyl from *ortho* to *para* resulted in decreased inhibitory activity.

#### 2.2.2 Kinetics studies

**FIGURE 2:**
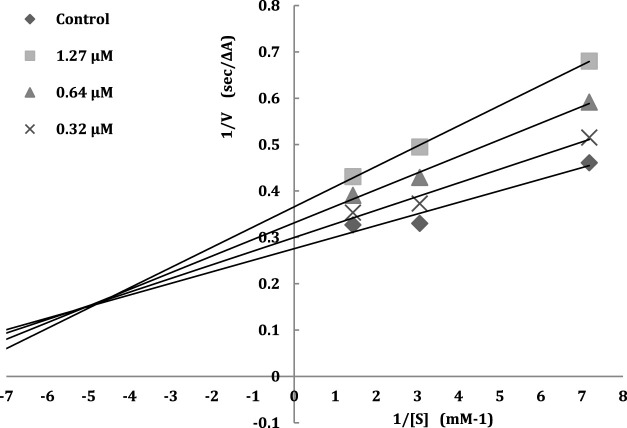
The Lineweavere-Burk plot for the inhibition of AChE by compound **5f** with increasing substrate concentration.

The inhibition model of acetylcholinesterase can be categorized into irreversible-type inhibition and reversible-type inhibition. Therefore, to achieve further insight into the mode of interaction of designed compounds on AChE, the rate of enzyme activity was measured at different concentrations of the most potent compound **5f** 0.32, 0.64, 1.27 μM and in the presence of substrate (ATCh). The type of inhibition can be concluded with a graphical analysis of the Lineweaver-Burk reciprocal plots (Baharloo et al., 2015). The higher concentration of inhibitor showed increasing slopes and intercepts. This proves a mixed-type inhibitory pattern on AChE (Figure 2).

#### 2.2.3 Inhibition of β-amyloid aggregation of selected compounds

The aggregation of the β-amyloid peptide is a negative event in the pathogenesis of Alzheimer’s disease, so, anti-amyloid therapy is an important factor in the treatment of Alzheimer’s. To evaluate the anti-aggregating activity of designed compounds, the five most potent derivatives, namely **5a**, **5f**, **5h**, **5i,** and **5l** were selected and evaluated against self-induced and AChE-induced Aβ aggregation. For this purpose, we used the thioflavin T (ThT) assay. Donepezil was used as a reference compound. The obtained results showed that the tested 3-aminobenzofuran compounds (**5a**, **5f**, **5h**, **5i,** and **5l**) at 10 μM concentration exhibited good inhibitory activity of self-induced Aβ_1–42_ aggregation (17.6%, 29.8%, 38.8%, 24.8%, and 25.7% inhibition, respectively) compared to donepezil as the reference drug (14.9%). Compounds **5f** and **5h** were about 2-folds more effective than donepezil in the inhibition of Aβ aggregation (Table 2). At 100 µM concentration, compounds **5a**, **5f**, **5h**, **5i,** and **5l** showed moderate to good activity to inhibit AChE-induced Aβ aggregation. Notably, compounds **5f** and **5h** were more potent (30.1% and 35.6, respectively) than the reference drug (25.7%). The ThT assay indicated that target compounds could inhibit the self-induced Aβ_1–42_ aggregation and AChE-induced Aβ aggregation.

**TABLE 2:** Inhibition of self-induced and AChE-induced Aβ_1–42_ aggregation by selected compounds.

Compounds	Inhibition of Aβ aggregation (%)	
	Self-induced^a^	AChE-induced^b^
**5a**	17.6 ± 3.8	22.4 ± 1.6
**5f**	29.8 ± 1.8	30.1 ± 1.9
**5h**	38.8 ± 2.8	35.6 ± 1.5
**5i**	24.8 ± 1.5	26.5 ± 3.8
**5l**	25.7 ± 2.7	22.4 ± 2.3
Donepezil	14.9 ± 2.5	25.7 ± 1.9

a Inhibition of Aβ_1–42_ aggregation was produced by the tested compound at 10 μM concentration. Values are expressed as means ± SEM of three experiments.

b Co-aggregation inhibition of Aβ_1–42_ and AChE (0.01 u/ml) by the tested compounds at 100 μM concentration was detected by the ThT assay. Values are expressed as means ± SEM of three experiments.

#### 2.2.4 Neuronal cell treatment and viability measurements

Safety is an important factor for CNS agents. In order to evaluate the cytotoxic effect of selected compounds **5a**, **5f**, **5h**, **5i,** and **5l**, the in-vitro cell viability assay was investigated on the PC12 cell line using the MTT (3-(4,5-dimethylthiazol-2-yl)-2,5-diphenyltetrazolium bromide) assay. The PC12 cells were incubated with varying concentrations (0.01–100 μM) of the test compounds for 24 h. The obtained results revealed that all tested compounds were nontoxic to PC12 cells (Table 3). The results are expressed as the percentage of viable cells.

**TABLE 3:** Effects of compounds **5a, 5f, 5h, 5i,** and **5l** on cell viability in PC12 cells.

Compounds	Viability (%) of PC12 cells^a^ ^,^ ^b^
**5a**	85.9 ± 1.5
**5f**	86.4 ± 2.6
**5h**	88.2 ± 2.4
**5i**	90.2 ± 4.1
**5l**	87.1 ± 2.9

a Cell viability is expressed as the mean percentage of viable cells.

b Mean percentage of viable cells compared with the untreated cells was determined to be 56.2 ± 1.96%.

#### 2.2.5 Molecular docking studies

**FIGURE 3:**
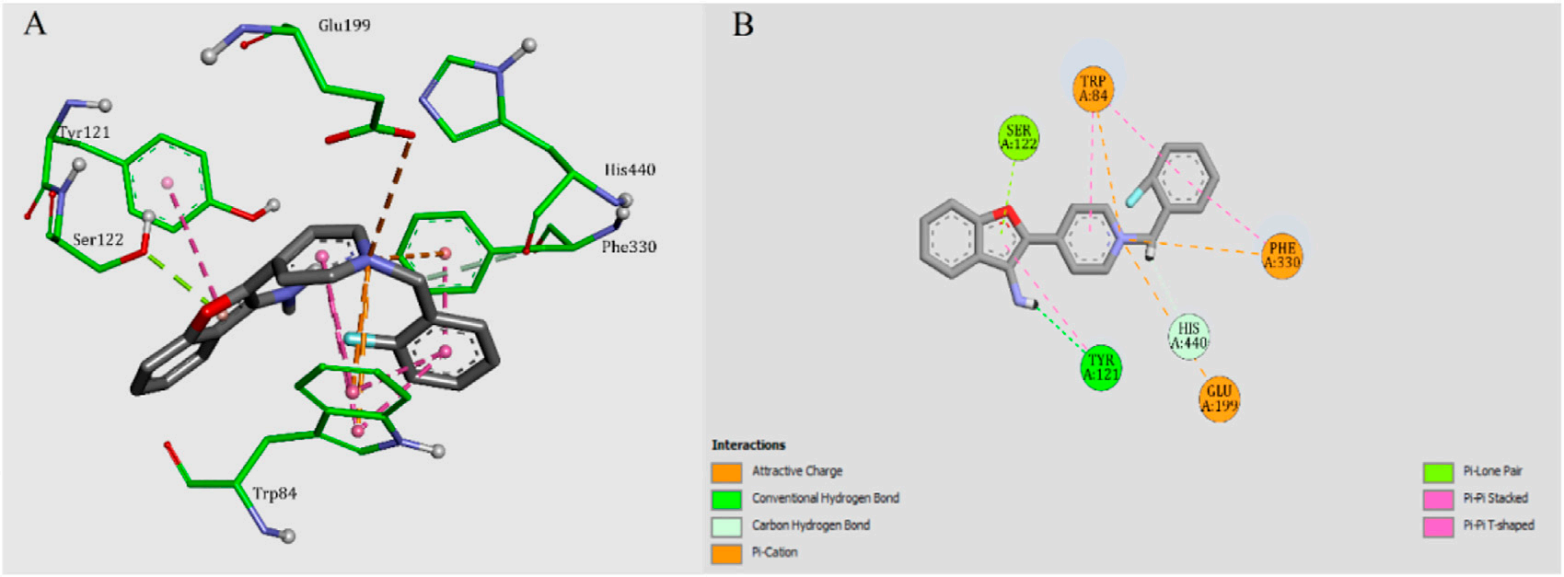
**(A)** 3D mode of interactions of compound **5f** with AChE. **(B)** 2D docking models of compound **5f** with AChE.

To get a better insight into the binding patterns between AChE and our new 3-aminobenzofuran derivatives, docking simulation were performed for the most active compound **5f** in the active site of AChE (PDB code: 1EVE) by using AutoDock software. The two- and three-dimensional modes of interactions were prepared and illustrated in Figure 3. Compound **5f** was anchored in the mid-gorge by some attractive charge among the Trp84, Phe330, and Glu199. Moreover, the pyridine ring was stacked against Trp84. The pendant 2-fluouropheny ring was sandwiched in between Trp84 and Phe330. The amino benzofuran ring made a T-shape stacking with Tyr121. A hydrogen bond between amino function and Tyr121 could stabilize such interaction.

## 3 Experimental

### 3.1 Chemistry

All reagents and solvents were obtained from commercial suppliers and used without purification. ^1^H NMR and ^13^C NMR spectra were acquired on a Bruker 500, 300 MHz NMR spectrometer and referenced to TMS. IR (KBr-disc) spectra were obtained by using a Bruker spectrometer. Melting points were determined by using a Kofler hot stage apparatus. All Reactions were monitored with thin layer TLC, using silica gel (60 Å) purchased from Merck. Mass analysis was carried out using an Agilent mass spectrometer. The elemental analysis for C, H, and N was carried out with an ElementarAnalysen system GmbH VarioEL.

#### 3.1.1 General procedure for the preparation of 2-((pyridine-4-yl) methoxy) benzonitrile 3

4-(Bromomethyl) pyridine **2** (10 mmol) was added to a mixture of 2-hydroxybenzonitrile **1** (10 mmol) and K_2_CO_3_ (20 mmol) in DMF (10 ml) and stirred at 80°C for 8 h. After completion of the reaction, the reaction mixture was slowly cooled down to room temperature and poured into ice and water. The precipitated solid was filtered, washed, and recrystallized from EtOH to afford 2-((pyridine-4-yl) methoxy) benzonitrile intermediate **3**.

White solid, yield: (83%). IR (KBr) (*ν*_*max*_/cm^−1^): 3388, 2220, 1591, 1484, 1444, 1384, 1285, 1156, 876, 754. ^1^H NMR (500 MHz, DMSO-*d*_6_): *δ* = 5.22 (s, 2H, CH_2_), 6.95 (d, *J* = 8.5 Hz, 2H, Ar-H), 7.06 (t, *J* = 7.5 Hz, 2H, Ar-H), 7.40 (d, *J* = 6.0 Hz, 2H, Ar-H), 7.50–7.54 (m, 1H, Ar-H), 7.62 (dd, *J* = 7.5, 1.5 Hz, 1H, Ar-H).

#### 3.1.2 General procedure for the preparation of 2-(pyridine-4-yl) 3-aminobenzofuran derivative 4

A mixture of intermediate **3** (3 mmol) and potassium *tert*-butoxide (5 mmol) in DMF (5 ml) was stirred at 80°C for 5 h. After the completion of the reaction, the reaction mixture was cooled down to room temperature and was poured into ice and water. The precipitated solid was filtered, washed, and recrystallized from EtOH to afford 2-(pyridine-4-yl) 3-aminobenzofuran **4**.

Yellow solid, yield: (73%). IR (KBr) (*ν*_*max*_/cm^−1^): 3360, 3311, 3191, 1596, 1409, 1355, 1274, 1212, 1166, 1143, 993, 822, 734, 682, 600, 527. ^1^H NMR (500 MHz, DMSO-*d*_6_): *δ* = 5.83 (s, 2H, CH_2_), 7.24 (t, *J* = 10.0 Hz, 1H, Ar-H), 7.38 (t, *J* = 10.0 Hz, 1H, Ar-H), 7.48 (d, *J* = 10.0 Hz, 1H, Ar-H), 7.65 (d, *J* = 5.0 Hz, 2H, Ar-H), 7.91 (d, *J* = 10.0 Hz, 1H, Ar-H), 8.53 (d, *J* = 5.0 Hz, 2H, Ar-H).

#### 3.1.3 General procedure for the preparation of *N*-benzyl-4-(3-aminobenzofuran-2-yl) pyridinium bromides (**5a-p**)

A mixture of 2-(pyridine-4-yl)-3-aminobenzofuran **4** (1 mmol) and the appropriate benzyl chloride derivatives (1.2 mmol) in dry acetonitrile (7 ml) was heated under reflux for 1–6 h. The precipitated solid was collected by filtration and washed with *n*-hexane (5 ml) to give **5a-p** in 56%–74% yields.

##### 3.1.3.1 4-(3-Aminobenzofuran-2-yl)-1-benzylpyridin-1-ium chloride (**5a**)

White solid, yield: (72%); mp 160–162°C. IR (KBr) (*ν*_*max*_/cm^−1^): 3293, 3158, 3033, 1631, 1592, 1542, 1312, 1215, 1160, 1100, 1030, 875, 834, 753, 690. ^1^H NMR (500 MHz, DMSO-*d*_6_): *δ* = 5.66 (s, 2H, CH_2_), 7.29 (t, *J* = 6.5 Hz, 1H, Ar-H), 7.43–7.46 (m, 5H, Ar-H), 7.51 (s, 4H, Ar-H), 7.97 (s, 2H, NH_2_), 8.10 (d, *J* = 7.5 Hz, 1H, Ar-H), 8.79 (d, *J* = 6.0 Hz, 2H, Ar-H). ^13^C NMR (125 MHz, DMSO-*d*_*6*_): *δ* = 60.7, 111.5, 116.7, 122.9, 128.2, 128.3, 128.6, 129.0, 130.4, 135.1, 140.1, 140.3, 142.1, 142.6, 142.7, 154.6. Anal. Calcd. for C_20_H_17_ClN_2_O, C, 71.32; H, 5.09; N, 8.32; Found C, 71.60; H, 4.79; N, 8.10.

##### 3.1.3.2 4-(3-Aminobenzofuran-2-yl)-1-(2-methylbenzyl) pyridin-1-ium chloride (**5b**)

White solid, yield: (67%); mp 182–184°C. IR (KBr) (*ν*_*max*_/cm^−1^): 3326, 3145, 1722, 1633, 1592, 1541, 1452, 1315, 1197, 1149, 1102, 1031, 968, 837, 746, 655. ^1^H NMR (500 MHz, DMSO-*d*_6_): *δ* = 2.31 (s, 3H, CH_3_), 5.70 (s, 2H, CH_2_), 7.11 (d, *J* = 6.5 Hz, 1H, Ar-H), 7.25–7.30 (m, 4H, Ar-H), 7.43 (s, 2H, Ar-H), 7.54 (d, *J* = 12.0 Hz, 2H, Ar-H), 8.00 (s, 2H, NH_2_), 8.11 (d, *J* = 7.0 Hz, 1H, Ar-H), 8.62 (d, *J* = 4.0 Hz, 2H, Ar-H). ^13^C NMR (125 MHz, DMSO-*d*_*6*_): *δ* = 18.7, 61.3, 111.7, 116.6, 116.8, 121.9, 122.1, 122.7, 126.5, 128.2, 128.8, 130.7, 133.2, 136.5, 140.2, 140.4, 142.2, 142.9, 154.6. Anal. Calcd. for C_21_H_19_ClN_2_O, C, 71.89; H, 5.46; N, 7.98; Found C, 72.06; H, 5.22; N, 8.15.

##### 3.1.3.3 4-(3-Aminobenzofuran-2-yl)-1-(3-methylbenzyl) pyridin-1-ium chloride (**5c**)

White solid, yield: (56%); mp 190–192°C. IR (KBr) (*ν*_*max*_/cm^−1^): 3122, 1974, 1639, 1542, 1226, 1162, 1048, 844, 762, 653. ^1^H NMR (500 MHz, DMSO-*d*_6_): *δ* = 2.41 (s, 3H, CH_3_), 5.59 (s, 2H, CH_2_), 7.40 (d, *J* = 7.0 Hz, 2H, Ar-H), 7.47 (d, *J* = 7.0 Hz, 2H, Ar-H), 7.50 (d, *J* = 10.5 Hz, 2H, Ar-H), 7.70 (t, *J* = 8.0 Hz, 1H, Ar-H), 7.74 (d, *J* = 8.0 Hz, 1H, Ar-H), 7.80 (d, *J* = 4.5 Hz, 1H, Ar-H), 8.22 (s, 2H, NH_2_), 8.35 (d, *J* = 8.0 Hz, 1H, Ar-H), 8.96 (d, *J* = 7.0 Hz, 2H, Ar-H). ^13^C NMR (125 MHz, DMSO-*d*_*6*_): *δ* = 20.9, 60.7, 116.6, 116.8, 122.1, 122.7, 125.4, 128.1, 128.7, 128.8, 129.5, 130.4, 135.1, 138.4, 140.3, 140.4, 141.7, 142.1, 154.6. Anal. Calcd. for C_21_H_19_ClN_2_O, C, 71.89; H, 5.46; N, 7.98; Found C, 72.02; H, 5.83; N, 7.77.

##### 3.1.3.4 4-(3-Aminobenzofuran-2-yl)-1-(4-methylbenzyl) pyridin-1-ium chloride (**5d**)

White solid, yield: (57%); mp 220–222°C. IR (KBr) (*ν*_*max*_/cm^−1^): 3313, 3136, 3038, 1643, 1537, 1350, 1154, 1028, 829, 752, 666. ^1^H NMR (500 MHz, DMSO-*d*_6_): *δ* = 2.29 (s, 3H, CH_3_), 5.58 (s, 2H, CH_2_), 7.23 (d, *J* = 7.5 Hz, 2H, Ar-H), 7.28 (t, *J* = 7.0 Hz, 2H, Ar-H), 7.41 (d, *J* = 7.5 Hz, 2H, Ar-H), 7.50 (t, *J* = 8.0 Hz, 1H, Ar-H), 7.54 (d, *J* = 8.0 Hz, 1H, Ar-H), 7.59 (s, 1H, Ar-H), 8.01 (s, 2H, NH_2_), 8.15 (d, *J* = 8.0 Hz, 1H, Ar-H), 8.76 (d, *J* = 6.5 Hz, 2H, Ar-H). ^13^C NMR (125 MHz, DMSO-*d*_*6*_): *δ* = 20.6, 60.6, 116.6, 122.3, 122.4, 122.7, 128.1, 128.3, 129.5, 130.4, 132.2, 138.4, 140.2, 142.0, 142.4, 142.5, 154.5. Anal. Calcd. for C_21_H_19_ClN_2_O, C, 71.89; H, 5.46; N, 7.98; Found C, 71.57; H, 5.80; N, 7.61.

##### 3.1.3.5 4-(3-Aminobenzofuran-2-yl)-1-(3-methoxybenzyl) pyridin-1-ium chloride (**5e**)

White solid, yield: (61%); mp 210–212°C. IR (KBr) (*ν*_*max*_/cm^−1^): 3134, 1638, 1594, 1541, 1310, 1265, 1223, 1160, 1103, 1042, 840, 754, 694. ^1^H NMR (500 MHz, DMSO-*d*_6_): *δ* = 3.74 (s, 3H, OCH_3_), 5.61 (s, 2H, CH_2_), 6.93 (d, *J* = 8.5 Hz, 1H, Ar-H), 7.08 (d, *J* = 7.0 Hz, 1H, Ar-H), 7.18 (s, 1H, Ar-H), 7.24 (t, *J* = 7.5 Hz, 1H, Ar-H), 7.32 (t, *J* = 7.5 Hz, 1H, Ar-H), 7.45 (d, *J* = 8.0 Hz, 1H, Ar-H), 7.49 (d, *J* = 8.0 Hz, 1H, Ar-H), 7.69 (s, 2H, Ar-H), 8.00 (s, 2H, NH_2_), 8.19 (d, *J* = 7.5 Hz, 1H, Ar-H), 8.82 (d, *J* = 6.5 Hz, 2H, Ar-H). ^13^C NMR (125 MHz, DMSO-*d*_*6*_): *δ* = 55.2, 60.5, 111.5, 114.2, 116.6, 120.4, 122.3, 122.4, 122.7, 128.1, 130.3, 136.6, 140.3, 140.5, 141.9, 142.5, 142.6, 154.6, 159.5. Anal. Calcd. for C_21_H_19_ClN_2_O_2_, C, 68.76; H, 5.22; N, 7.64; Found C, 69.05; H, 5.51; N, 7.30.

##### 3.1.3.6 4-(3-Aminobenzofuran-2-yl)-1-(2-fluorobenzyl) pyridin-1-ium chloride (**5f**)

White solid, yield: (73%); mp 253–255°C. IR (KBr) (*ν*_*max*_/cm^−1^): 3175, 2982, 1635, 1541, 1314, 1225, 1159, 1107, 1040, 833, 757, 648. ^1^H NMR (500 MHz, DMSO-*d*_6_): *δ* = 5.74 (s, 2H, CH_2_), 7.26–7.32 (m, 3H, Ar-H), 7.46–7.55 (m, 3H, Ar-H), 7.58 (d, *J* = 7.5 Hz, 1H, Ar-H), 7.69 (s, 2H, Ar-H), 8.02 (s, 2H, NH_2_), 8.17 (d, *J* = 8.0 Hz, 1H, Ar-H), 8.68 (d, *J* = 6.5 Hz, 2H, Ar-H). ^13^C NMR (125 MHz, DMSO-*d*_*6*_): *δ* = 55.3, 115.8, 116.6, 122.1, 122.5, 125.1, 128.1, 130.5, 130.9, 131.5, 140.7, 141.8, 142.1, 142.7, 143.6, 154.7, 159.3, 161.3 (d, *J* = 240 Hz). Anal. Calcd. for C_20_H_16_ClFN_2_O, C, 67.70; H, 4.55; N, 7.90; Found C, 67.44; H, 4.24; N, 7.55.

##### 3.1.3.7 4-(3-Aminobenzofuran-2-yl)-1-(3-fluorobenzyl) pyridin-1-ium chloride (**5g**)

White solid, yield: (65%); mp 267–269°C. IR (KBr) (*ν*_*max*_/cm^−1^): 3298, 3167, 2981, 1630, 1543, 1455, 1313, 1253, 1158, 1098, 1036, 879, 835, 750, 692. ^1^H NMR (500 MHz, DMSO-*d*_6_): *δ* = 5.66 (s, 2H, CH_2_), 7.24 (t, *J* = 8.0 Hz, 1H, Ar-H), 7.29 (t, *J* = 7.0 Hz, 1H, Ar-H), 7.36 (d, *J* = 7.0 Hz, 1H, Ar-H), 7.43–7.47 (m, 3H, Ar-H), 7.48–7.54 (m, 3H, Ar-H), 7.97 (s, 2H, NH_2_), 8.10 (d, *J* = 8.0 Hz, 1H, Ar-H), 8.80 (d, *J* = 6.5 Hz, 2H, Ar-H). ^13^C NMR (125 MHz, DMSO-*d*_*6*_): *δ* = 60.0, 115.3, 115.5, 115.7, 116.7, 122.0, 122.6, 124.4, 128.2, 130.5, 131.2, 137.3, 140.3, 140.4, 142.2, 142.6, 142.7, 159.6 (d, *J* = 237 Hz). Anal. Calcd. for C_20_H_16_ClFN_2_O, C, 67.70; H, 4.55; N, 7.90; Found C, 67.48; H, 4.24; N, 7.66.

##### 3.1.3.8 4-(3-Aminobenzofuran-2-yl)-1-(4-fluorobenzyl) pyridin-1-ium chloride (**5h**)

White solid, yield: (71%); mp 258–260°C. IR (KBr) (*ν*_*max*_/cm^−1^): 3296, 3159, 2977, 1631, 1543, 1313, 1226, 1099, 1032, 876, 826, 753, 658. ^1^H NMR (500 MHz, DMSO-*d*_6_): *δ* = 5.63 (s, 2H, CH_2_), 7.29 (t, *J* = 9.0 Hz, 3H, Ar-H), 7.40 (d, *J* = 6.0 Hz, 2H, Ar-H), 7.50 (d, *J* = 8.0 Hz, 1H, Ar-H), 7.54 (d, *J* = 7.5 Hz, 1H, Ar-H), 7.62 (t, *J* = 6.5 Hz, 2H, Ar-H), 7.96 (s, 2H, NH_2_), 8.09 (d, *J* = 7.5 Hz, 1H, Ar-H), 8.79 (d, *J* = 7.0 Hz, 2H, Ar-H). ^13^C NMR (125 MHz, DMSO-*d*_*6*_): *δ* = 59.9, 115.8, 116.0, 116.8, 122.6, 128.1, 130.4, 130.8, 131.3, 140.2, 140.3, 142.1, 142.5, 142.6, 154.6, 161.3 (d, *J* = 237 Hz). Anal. Calcd. for C_20_H_16_ClFN_2_O, C, 67.70; H, 4.55; N, 7.90; Found C, 67.45; H, 4.89; N, 8.12.

##### 3.1.3.9 4-(3-Aminobenzofuran-2-yl)-1-(2-chlorobenzyl) pyridin-1-ium chloride (**5i**)

White solid, yield: (64%); mp 135–137°C. IR (KBr) (*ν*_*max*_/cm^−1^): 3347, 3290, 3171, 3081, 1637, 1592, 1545, 1467, 1314, 1216, 1158, 1101, 1042, 871, 827, 751, 671. ^1^H NMR (500 MHz, DMSO-*d*_6_): *δ* = 5.76 (s, 2H, CH_2_), 7.30 (t, *J* = 7.0 Hz, 1H, Ar-H), 7.41 (d, *J* = 7.0 Hz, 2H, Ar-H), 7.45 (d, *J* = 8.0 Hz, 2H, Ar-H), 7.52 (t, *J* = 8.0 Hz, 2H, Ar-H), 7.57 (t, *J* = 8.0 Hz, 2H, Ar-H), 7.99 (s, 2H, NH_2_), 8.11 (d, *J* = 7.5 Hz, 1H, Ar-H), 8.65 (d, *J* = 6.5 Hz, 2H, Ar-H). ^13^C NMR (125 MHz, DMSO-*d*_*6*_): *δ* = 58.7, 115.7, 116.4, 122.2, 122.6, 128.0, 128.2, 130.0, 130.6, 130.7, 132.4, 132.8, 140.6, 140.7, 142.2, 142.9, 143.0, 154.7. Anal. Calcd. for C_20_H_16_Cl_2_N_2_O, C, 64.70; H, 4.34; N, 7.55; Found C, 64.56; H, 4.02; N, 7.34.

##### 3.1.3.10 4-(3-Aminobenzofuran-2-yl)-1-(3-chlorobenzyl) pyridin-1-ium chloride (**5j**)

White solid, yield: (59%); mp 180–182°C. IR (KBr) (*ν*_*max*_/cm^−1^): 3291, 3139, 1635, 1540, 1313, 1208, 1160, 1097, 1032, 875, 833, 759, 688. ^1^H NMR (500 MHz, DMSO-*d*_6_): *δ* = 5.64 (s, 2H, CH_2_), 7.29 (t, *J* = 7.5 Hz, 1H, Ar-H), 7.43 (d, *J* = 5.5 Hz, 2H, Ar-H), 7.45–7.54 (m, 5H, Ar-H), 7.68 (s, 1H, Ar-H), 7.97 (d, *J* = 5.5 Hz, 2H, Ar-H), 8.09 (d, *J* = 7.5 Hz, 1H, Ar-H), 8.80 (d, *J* = 6.5 Hz, 2H, Ar-H). ^13^C NMR (125 MHz, DMSO-*d*_*6*_): *δ* = 59.9, 116.8, 121.9, 122.1, 122.6, 127.1, 128.2, 128.8, 130.5, 130.9, 133.5, 137.4, 140.3, 140.4, 142.2, 142.6, 142.7, 154.6. Anal. Calcd. for C_20_H_16_Cl_2_N_2_O, C, 64.70; H, 4.34; N, 7.55; Found C, 65.00; H, 4.63; N, 7.24.

##### 3.1.3.11 4-(3-Aminobenzofuran-2-yl)-1-(4-chlorobenzyl) pyridin-1-ium chloride (**5k**)

Yellow solid, yield: (69%); mp 192–194°C. IR (KBr) (*ν*_*max*_/cm^−1^): 3145, 1640, 1593, 1541, 1317, 1217, 1164, 1098, 1027, 825, 761. ^1^H NMR (500 MHz, DMSO-*d*_6_): *δ* = 5.64 (s, 2H, CH_2_), 7.28 (t, *J* = 7.5 Hz, 1H, Ar-H), 7.50 (d, *J* = 7.6 Hz, 2H, Ar-H), 7.53 (d, *J* = 7.6 Hz, 2H, Ar-H), 7.56–7.60 (m, 3H, Ar-H), 7.62 (d, *J* = 4.0 Hz, 1H, Ar-H), 8.02 (s, 2H, NH_2_), 8.15 (d, *J* = 8.0 Hz, 1H, Ar-H), 8.79 (d, *J* = 6.5 Hz, 2H, Ar-H). ^13^C NMR (125 MHz, DMSO-*d*_*6*_): *δ* = 59.8, 116.8, 122.1, 122.3, 122.5, 128.1, 129.0, 130.3, 133.7, 134.1, 140.5, 140.6, 142.1, 142.6, 142.7, 154.7. Anal. Calcd. for C_20_H_16_Cl_2_N_2_O, C, 64.70; H, 4.34; N, 7.55; Found C, 64.45; H, 4.09; N, 7.78.

##### 3.1.3.12 4-(3-Aminobenzofuran-2-yl)-1-(2-bromobenzyl) pyridin-1-ium chloride (**5l**)

White solid, yield: (61%); mp 191–193°C. IR (KBr) (*ν*_*max*_/cm^−1^): 3293, 3171, 3076, 1636, 1544, 1463, 1314, 1214, 1155, 1101, 1032, 870, 826, 750, 652. ^1^H NMR (500 MHz, DMSO-*d*_6_): *δ* = 5.73 (s, 2H, CH_2_), 7.31 (t, *J* = 8.0 Hz, 2H, Ar-H), 7.39 (t, *J* = 7.5 Hz, 2H, Ar-H), 7.47 (d, *J* = 6.5 Hz, 2H, Ar-H), 7.51 (d, *J* = 10.0 Hz, 1H, Ar-H), 7.54 (d, *J* = 8.5 Hz, 1H, Ar-H), 7.57 (d, *J* = 8.0 Hz, 1H, Ar-H), 7.99 (s, 2H, NH_2_), 8.11 (d, *J* = 8.0 Hz, 1H, Ar-H), 8.64 (d, *J* = 7.0 Hz, 2H, Ar-H). ^13^C NMR (125 MHz, DMSO-*d*_*6*_): *δ* = 60.9, 116.6, 122.0, 122.1, 122.6, 122.9, 128.2, 128.6, 130.3, 130.9, 133.2, 134.0, 140.6, 140.7, 142.3, 143.1, 143.9, 154.7. Anal. Calcd. for C_20_H_16_BrClN_2_O, C, 57.78; H, 3.88; N, 6.74; Found C, 57.45; H, 3.57; N, 6.47.

##### 3.1.3.13 4-(3-Aminobenzofuran-2-yl)-1-(3-bromobenzyl) pyridin-1-ium chloride (**5m**)

White solid, yield: (74%); mp 270–272°C. IR (KBr) (*ν*_*max*_/cm^−1^): 3292, 3159, 1634, 1539, 1313, 1160, 1098, 1032, 834, 757, 671. ^1^H NMR (500 MHz, DMSO-*d*_6_): *δ* = 5.63 (s, 2H, CH_2_), 7.30 (t, *J* = 7.0 Hz, 1H, Ar-H), 7.41 (d, *J* = 6.5 Hz, 2H, Ar-H), 7.51–7.57 (m, 3H, Ar-H), 7.61 (d, *J* = 7.5 Hz, 1H, Ar-H), 7.81 (s, 1H, Ar-H), 7.97 (s, 2H, NH_2_), 8.09 (d, *J* = 7.5 Hz, 2H, Ar-H), 8.79 (d, *J* = 6.0 Hz, 2H, Ar-H). ^13^C NMR (125 MHz, DMSO-*d*_*6*_): *δ* = 59.9, 115.6, 116.8, 121.9, 122.1, 122.6, 127.3, 128.2, 130.6, 131.2, 131.8, 137.6, 138.1, 140.3, 142.2, 142.6, 142.7, 154.6. Anal. Calcd. for C_20_H_16_BrClN_2_O, C, 57.78; H, 3.88; N, 6.74; Found C, 58.03; H, 3.61; N, 6.38.

##### 3.1.3.14 4-(3-Aminobenzofuran-2-yl)-1-(4-bromobenzyl) pyridin-1-ium chloride (**5n**)

White solid, yield: (68%); mp 254–257°C. IR (KBr) (*ν*_*max*_/cm^−1^): 3171, 1636, 1592, 1540, 1314, 1214, 1162, 1100, 1015, 823, 759. ^1^H NMR (500 MHz, DMSO-*d*_6_): *δ* = 5.63 (s, 2H, CH_2_), 7.29 (d, *J* = 7.5 Hz, 1H, Ar-H), 7.43 (d, *J* = 5.5 Hz, 2H, Ar-H), 7.49 (t, *J* = 8.0 Hz, 2H, Ar-H), 7.53 (d, *J* = 7.5 Hz, 2H, Ar-H), 7.64 (d, *J* = 7.5 Hz, 2H, Ar-H), 7.96 (s, 2H, NH_2_), 8.10 (d, *J* = 8.0 Hz, 1H, Ar-H), 8.77 (d, *J* = 6.5 Hz, 2H, Ar-H). ^13^C NMR (125 MHz, DMSO-*d*_6_): *δ* = 59.9, 116.6, 116.8, 121.9, 122.1, 122.3, 122.6, 128.1, 130.5, 132.0, 134.5, 140.3, 140.4, 142.1, 142.7, 154.6. Anal. Calcd. for C_20_H_16_BrClN_2_O, C, 57.78; H, 3.88; N, 6.74; Found C, 57.59; H, 4.02; N, 6.52.

##### 3.1.3.15 4-(3-Aminobenzofuran-2-yl)-1-(2-nitrobenzyl) pyridin-1-ium chloride (**5o**)

Yellow solid, yield: (67%); mp 148–150°C. IR (KBr) (*ν*_*max*_/cm^−1^): 3167, 2986, 1707, 1639, 1525, 1337, 1170, 1107, 1045, 854, 789, 736. ^1^H NMR (500 MHz, DMSO-*d*_6_): *δ* = 6.02 (s, 2H, CH_2_), 7.13 (d, *J* = 7.5 Hz, 1H, Ar-H), 7.32 (t, *J* = 8.0 Hz, 2H, Ar-H), 7.53 (d, *J* = 8.0 Hz, 2H, Ar-H), 7.57 (t, *J* = 7.0 Hz, 1H, Ar-H), 7.71 (d, *J* = 7.5 Hz, 1H, Ar-H), 7.80 (d, *J* = 7.5 Hz, 1H, Ar-H), 8.04 (s, 2H, NH_2_), 8.12 (d, *J* = 8.0 Hz, 1H, Ar-H), 8.24 (d, *J* = 7.5 Hz, 1H, Ar-H), 8.66 (d, *J* = 6.5 Hz, 2H, Ar-H). ^13^C NMR (125 MHz, DMSO-*d*_*6*_): *δ* = 58.2, 116.7, 122.0, 122.2, 122.6, 125.5, 128.2, 129.1, 129.9, 130.3, 130.6, 134.8, 140.7, 142.4, 143.3, 144.0, 147.3, 154.7. Anal. Calcd. for C_20_H_16_ClN_3_O_3_, C, 62.91; H, 4.22; N, 11.01; Found C, 63.11; H, 3.95; N, 11.35.

##### 3.1.3.16 4-(3-Aminobenzofuran-2-yl)-1-(4-nitrobenzyl) pyridin-1-ium chloride (**5p**)

Yellow solid, yield: (73%); mp 173–175°C. IR (KBr) (*ν*_*max*_/cm^−1^): 3302, 3178, 3104, 1637, 1533, 1340, 1213, 1165, 1103, 1031, 827, 753, 689. ^1^H NMR (500 MHz, DMSO-*d*_6_): *δ* = 5.81 (s, 2H, CH_2_), 7.31 (t, *J* = 7.0 Hz, 1H, Ar-H), 7.49–7.57 (m, 3H, Ar-H), 7.74 (d, *J* = 8.0 Hz, 2H, Ar-H), 7.99 (s, 2H, NH_2_), 8.10 (d, *J* = 7.5 Hz, 2H, Ar-H), 8.28 (d, *J* = 7.5 Hz, 2H, Ar-H), 8.79 (d, *J* = 6.0 Hz, 2H, Ar-H). ^13^C NMR (125 MHz, DMSO-*d*_*6*_): *δ* = 59.7, 116.8, 122.0, 122.2, 122.5, 123.9, 124.1, 128.2, 129.4, 130.6, 140.7, 142.2, 142.3, 142.8, 147.6, 154.7. Anal. Calcd. for C_20_H_16_ClN_3_O_3_, C, 62.91; H, 4.22; N, 11.01; Found C, 62.68; H, 4.51; N, 10.83.

### 3.2 *In vitro* biological evaluations

#### 3.2.1 Cholinesterase inhibition assay

The method described by Ellman was used to evaluate the inhibitory activity of compounds **5a-p** against AChE, according to procedures previously reported. AChE from E. electricus, and horse serum BuChE (eqBuChE, Sigma-Aldrich) 5,5-Dithiobis-(2-nitrobenzoic acid) (DTNB), and acetylthiocholine iodide (ATC) were purchased from Sigma Aldrich. Donepezil was used as a standard drug and all experiments were assayed in triplicate. In this procedure, five different concentrations of each compound were used at 25°C. To determine the IC_50_, 100 μl of DTNB, 3 ml phosphate buffer (0.1 M, pH = 8.0), 50 μl of the enzyme, and 50 μl of each compound were incubated in 24 well plates for 5 min followed by the addition of 20 μl of the substrate (acetylthiocholine iodide) and absorbance was detected at 415 nm for 6 min, and then the percent inhibition was plotted from inhibition curves. For the in-vitro BuChE assay similar method has been used.

#### 3.2.2 Kinetics study

To understand further insight into the inhibition model of compounds, the kinetic study was performed for the most active compound **5f** according to procedures previously reported (Baharloo et al., 2015). The experiment was assayed in triplicate.

#### 3.2.3 *In vitro* inhibition of self-induced and AChE-induced Aβ_1–42_ aggregation

To assess the inhibitory effect of compounds **5a, 5f, 5h, 5i,** and **5l** on the β-Amyloid aggregation, we used a thioflavin T (ThT)-based fluorescence assay to determine the self-induced and AChE-induced Aβ_1–42_ aggregation (Levine, 1993). Aβ_1–42_ (Sigma A9810) 50 μM was dissolved in ammonium hydroxide (1% *v/v*) for pre-fibrillation and incubated for 72 h at 37°C. Aβ_1–42_ (10 µl) in the absence and presence of human recombinant AChE (0.01 u/mL, Sigma C1682) were added to phosphate buffer (0.05 M, pH = 7.4) and incubated at 37°C for 48 h with or without target compounds **5a, 5f, 5h, 5i,** and **5l** (100 μM). Next, 50 μl of thioflavin T (ThT, 200 μM) containing 50 mM glycine-NaOH buffer (pH 8.5) was added. Fluorescences were recorded (λ_exc_ = 446 nm; λ_em_ = 490 nm) by Microplate Reader (Spectra Max). Donepezil (100 μM, Sigma D-6821) was used as a reference drug. The aggregation inhibition of the tested compounds was calculated on AChE or self-induced Aβ-aggregation according to the equation (1−IF_i_/IF_c_) × 100, where IF_i_ and IFc are the fluorescence intensities in the presence of inhibitors and in the absence of inhibitors, respectively.

#### 3.2.4 Cell viability assay

In order to evaluate cell viability of **5a, 5f, 5h, 5i,** and **5l**, the 3-(4,5-dimethylthiazol-2-yl)-2,5-diphenyl tetrazolium bromide (MTT) assay was performed using PC12 cell line. Cells were seeded in 96-well plates at a density of 5,000 cells/well for 24 h, and then cells were treated with tested compounds and glutamate (5 mM) and incubated at 37°C for 24 h. After that, the medium was removed and 20 μl of the MTT reagent (5 mg/ml) was added and incubated at 37 °C for 4 h. Then, the medium was replaced with 150 μl DMSO to solve formazan crystals. The absorbance was recorded by microplate reader apparatus (Biotek, Winooski, VT, United States) at 570 nm. The assay was performed in three independent experiments and triplicate.

#### 3.2.5 Docking simulations

The molecular docking studies were performed using the program Autodock Vina (Trott and Olson 2010). The crystal structure of acetyl cholinesterase (1EVE) was derived from the Protein Data Bank (http://www.rcsb.org/). The co-crystallized ligand and water molecules were deleted from the protein structure. The top 3-aminocoumarin derivative **5f** was selected for the docking study. The selected ligand was constructed using Marvin Sketch, 2012, Chem Axon (http://www.chemaxon.com). The center of the grid box were set as follow; x = 2.071, y = 63.616, z = 67.737, and the active site box were set at 15 Å × 15 Å × 15 Å. Molecular visualizations were carried out by Discovery Studio 4.5 client software (Accelrys, Inc., San Diego, CA, United States).

## 4 Conclusion

In the present study, a novel series of 3-aminobenzofuran derivatives with *N*-benzylpyridinium moiety were designed, synthesized, and evaluated as novel agents for the treatment of AD. The in-vitro assays revealed that most target compounds had moderate to good anti-AChE and -BuChE activity with no toxicity against PC12 cells. The best result was obtained from 2-fluorobenzyl derivative **5f** which exhibited potent AChE and BuChE inhibitory activity with IC_50_ = 0.64 and 0.55 μM, respectively. Moreover, compounds **5f** and **5h** have remarkable anti-aggregation activity compared to donepezil as the reference drug. Docking’s study demonstrated that 3-aminobenzofuran (hydrophobic aromatic fragment) can be positioned in the peripheral anionic site and the *N*-benzylpyridinium fragment bound to the catalytic anionic site of AChE. These results showed that compound **5f** could be considered a promising multifunctional derivative for further studies in the field of new anti-Alzheimer agents.

## Data Availability

The original contributions presented in the study are included in the article/Supplementary Materials; further inquiries can be directed to the corresponding author.
