# Supplementary material for: Novel 3-aminobenzofuran derivatives as multifunctional agents for the treatment of Alzheimer’s disease
Source: Front Chem. 2022 Aug 9;10:882191. doi: 10.3389/fchem.2022.882191 (PMC9395670; doi:10.3389/fchem.2022.882191)
Supplement: Supplementary file 1 [file DataSheet1.docx]

**Supplementary material**

# **Novel 3-aminobenzofuran derivatives as multifunctional agents for the treatment of Alzheimer's disease**


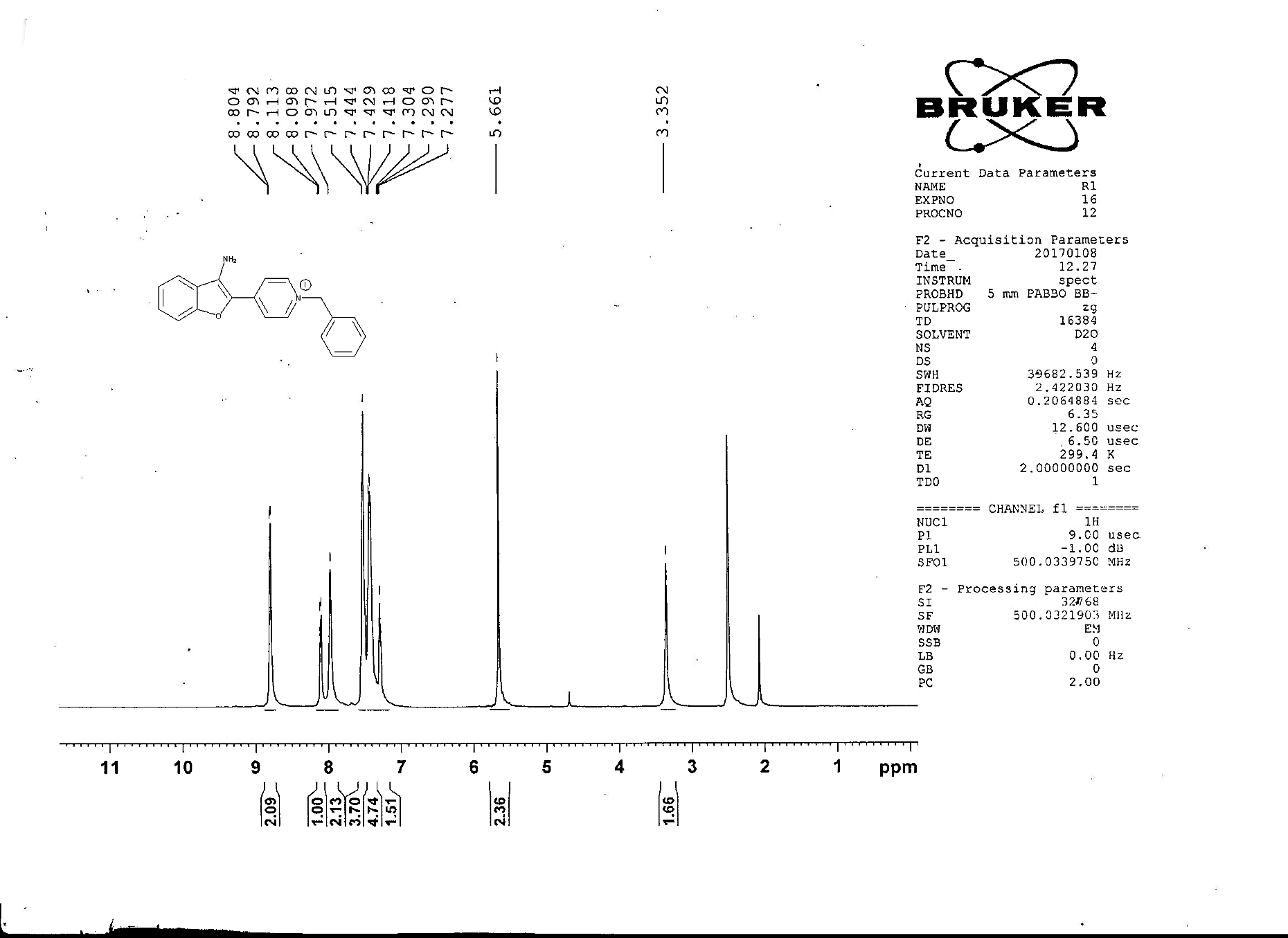
**
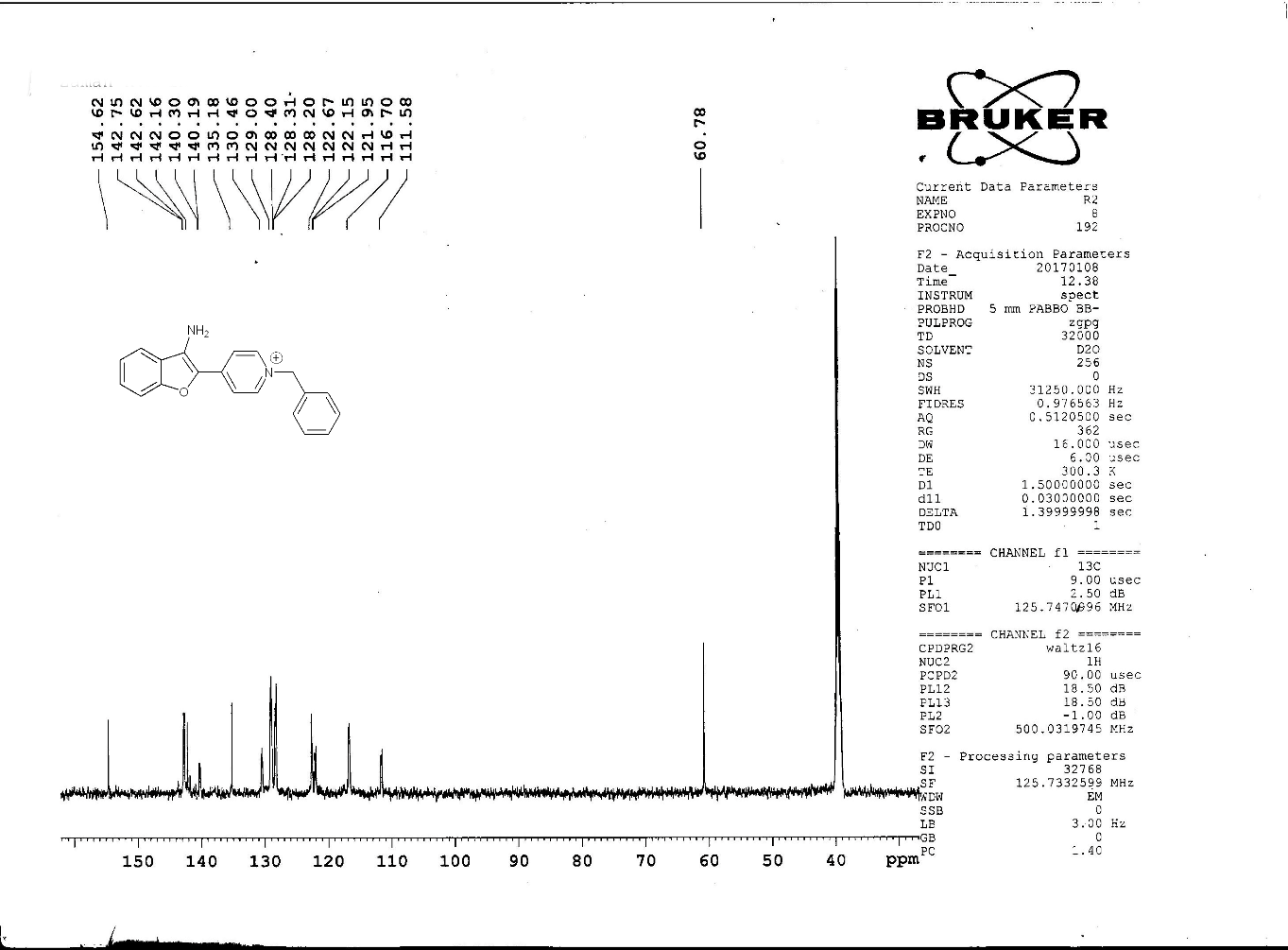
**

Figure S1. ^1^HNMR and ^13^CNMR spectra of compound **5a**

**
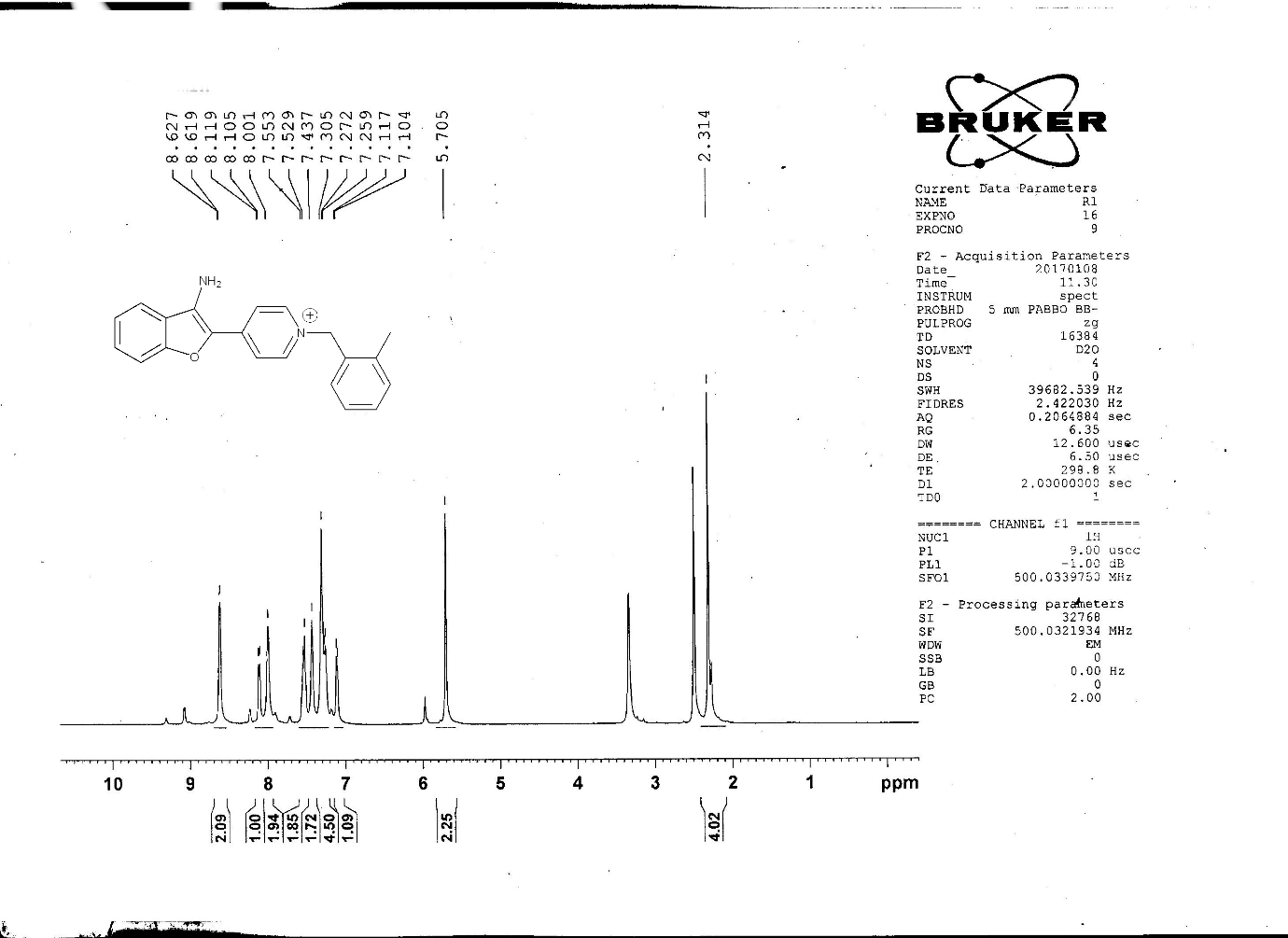
**

**
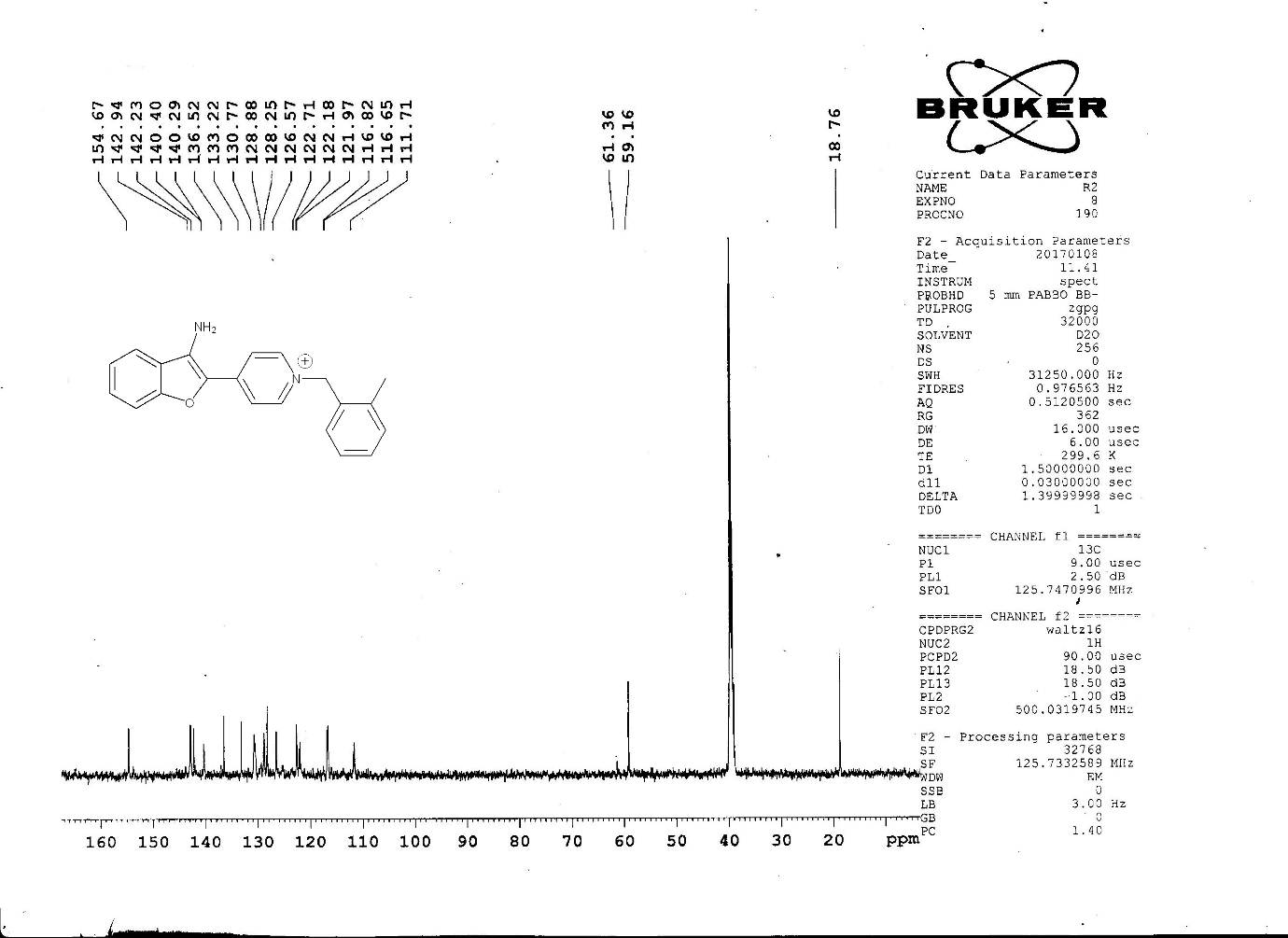
**

Figure S2. ^1^HNMR and ^13^CNMR spectra of compound **5b**

**
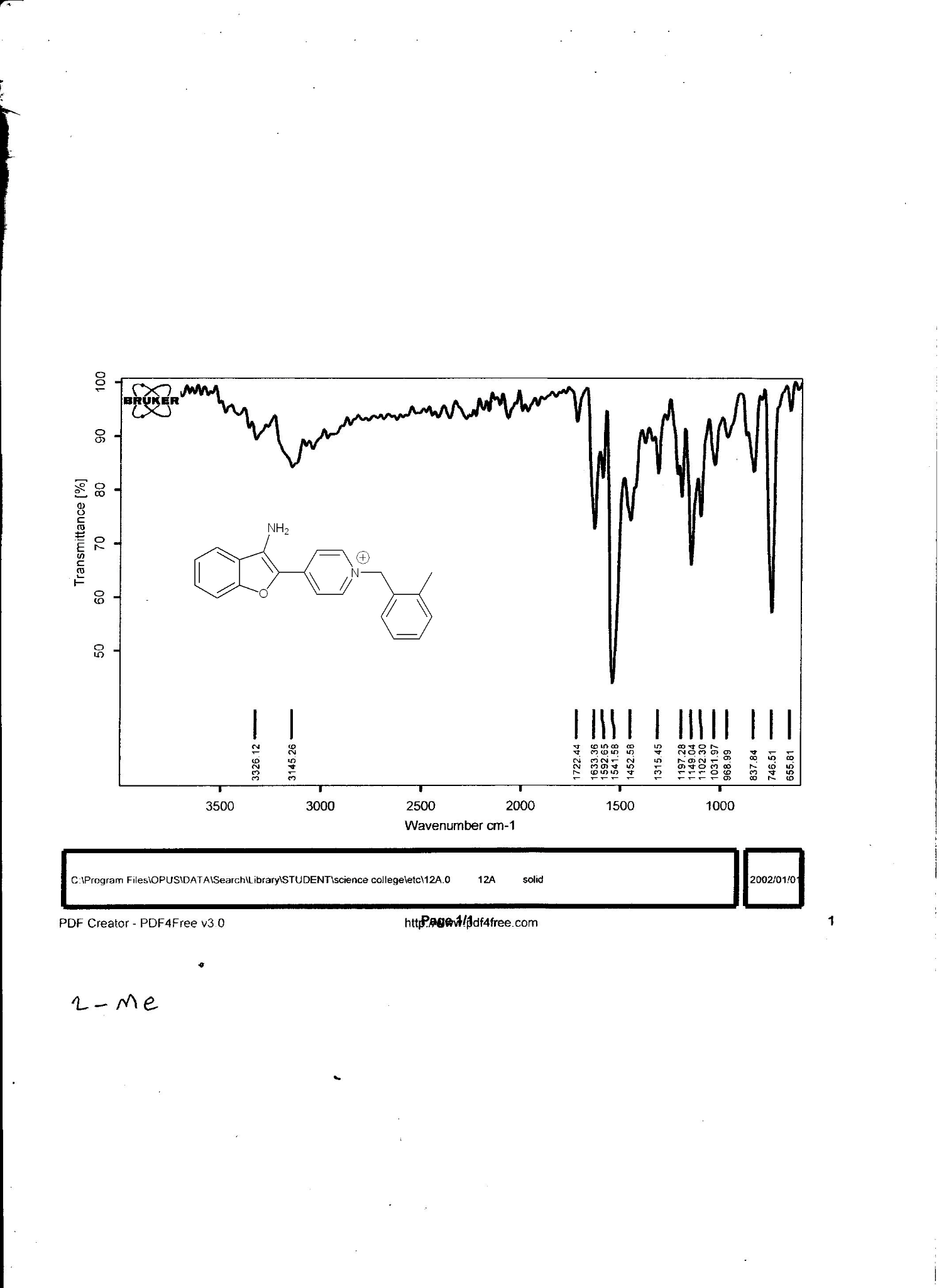
**

Figure S3. IR spectrum of compound **5b**

**
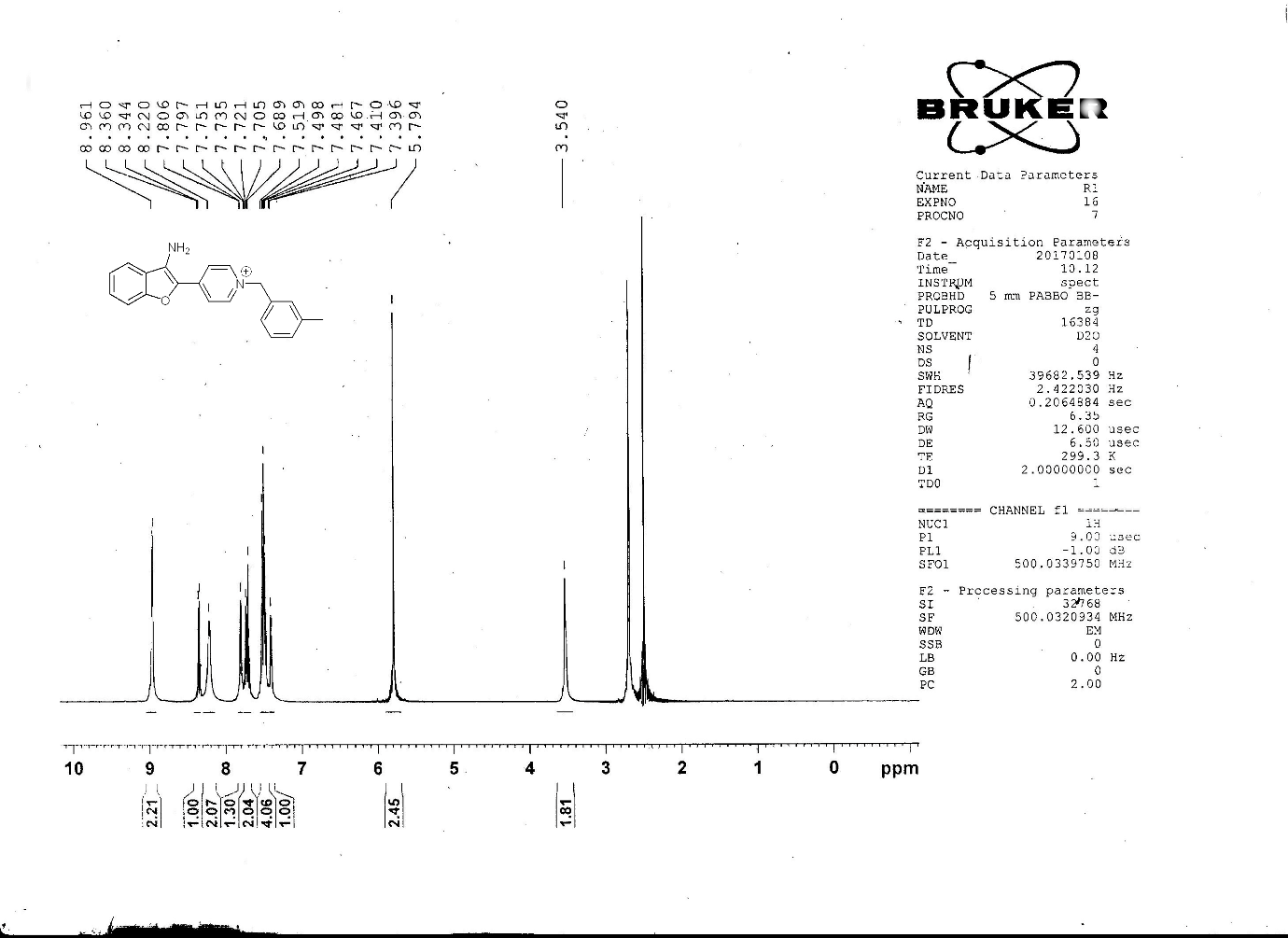
**


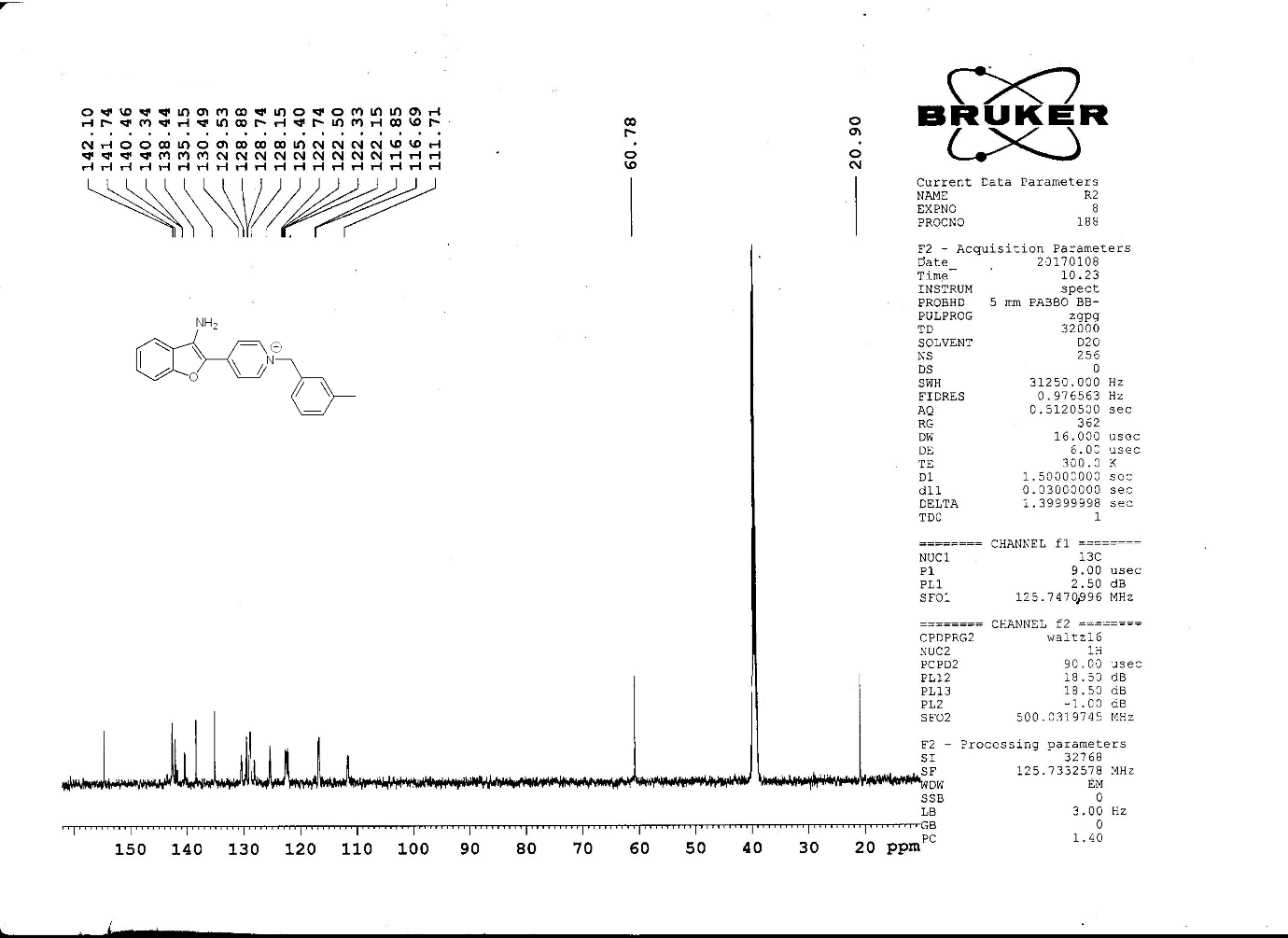


Figure S4. ^1^HNMR and ^13^CNMR spectra of compound **5c**

**
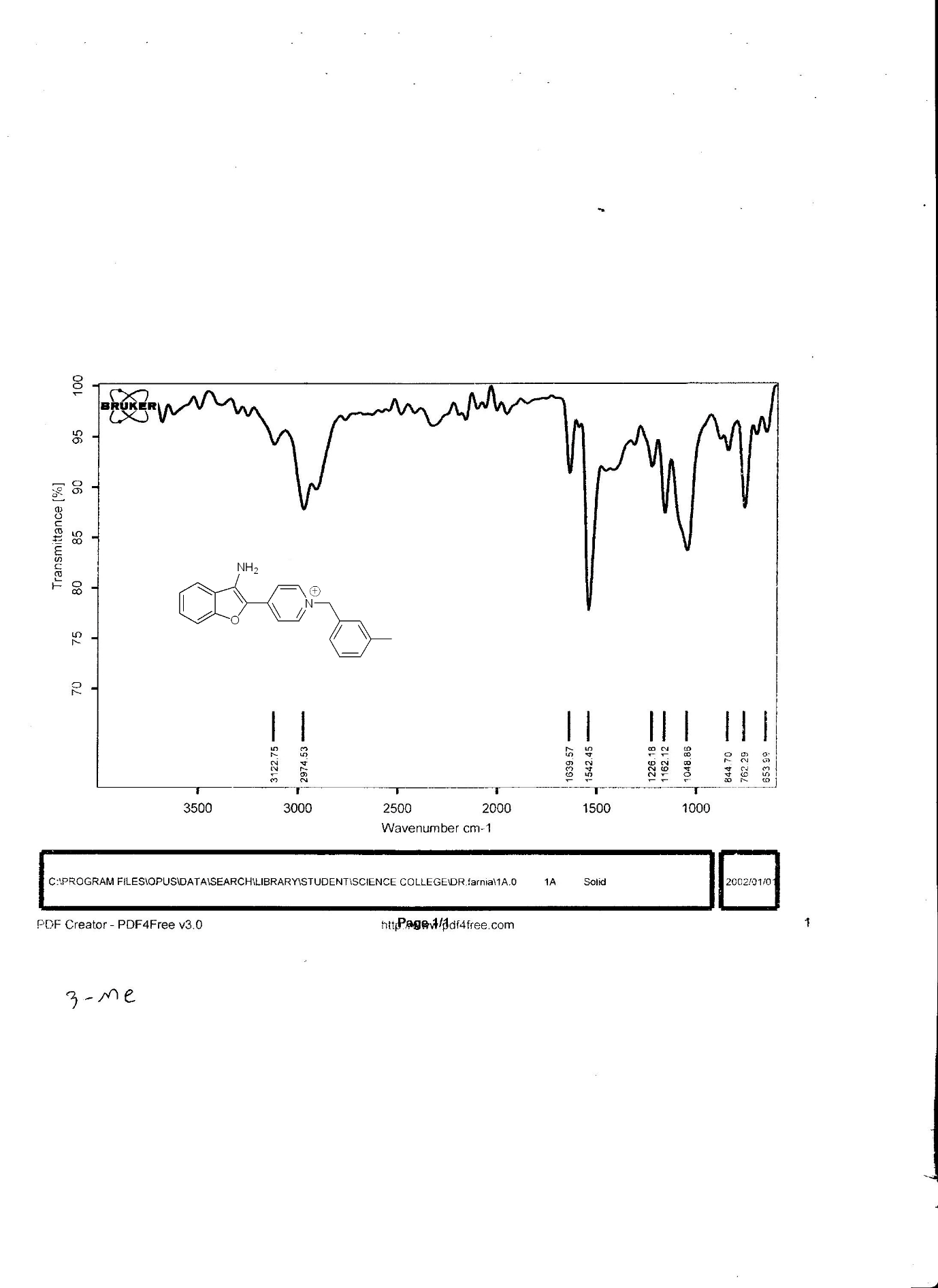
**

Figure S5. IR spectrum of compound **5c**

**
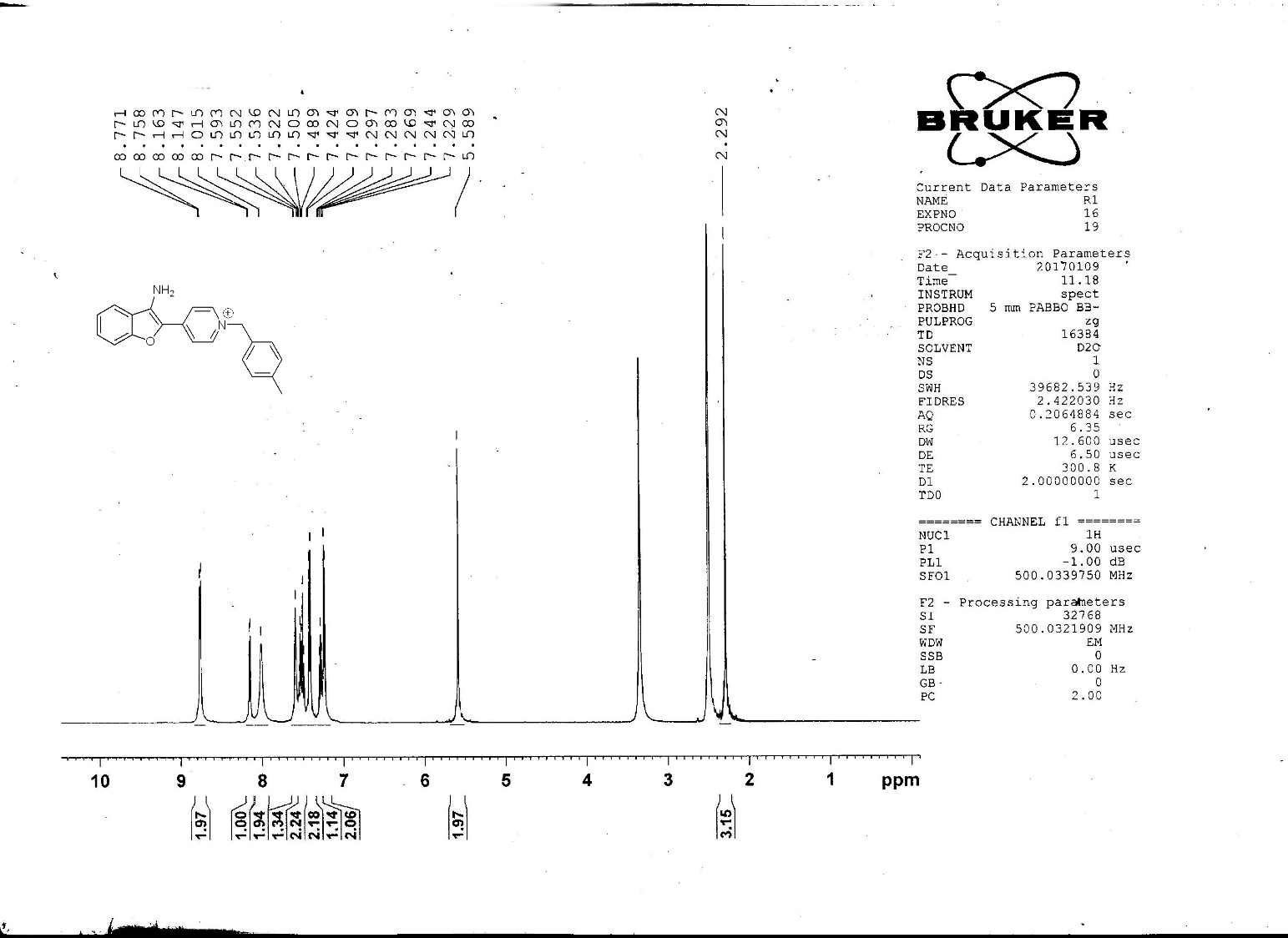
**


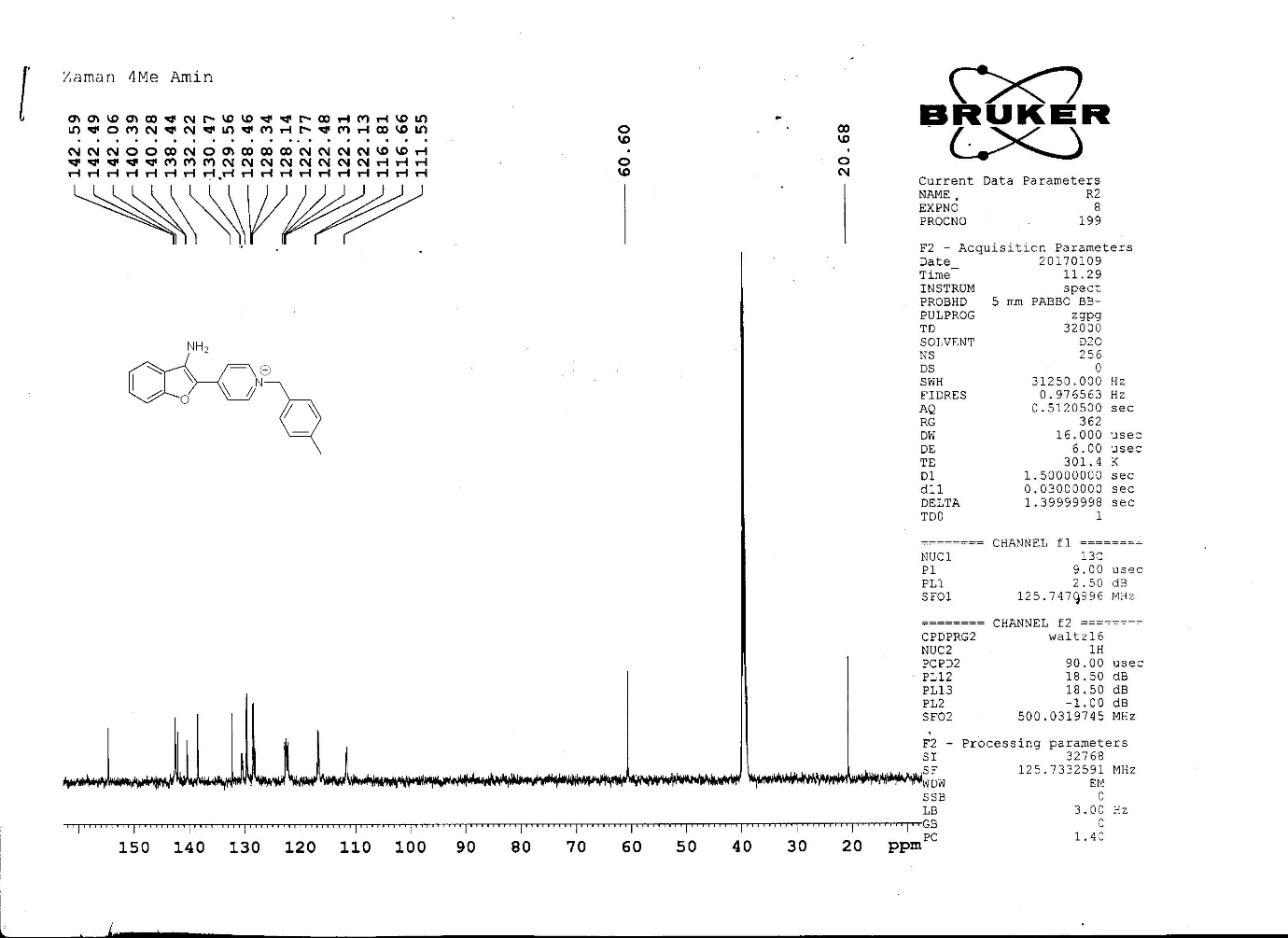


Figure S6. ^1^HNMR and ^13^CNMR spectra of compound **5d**

**
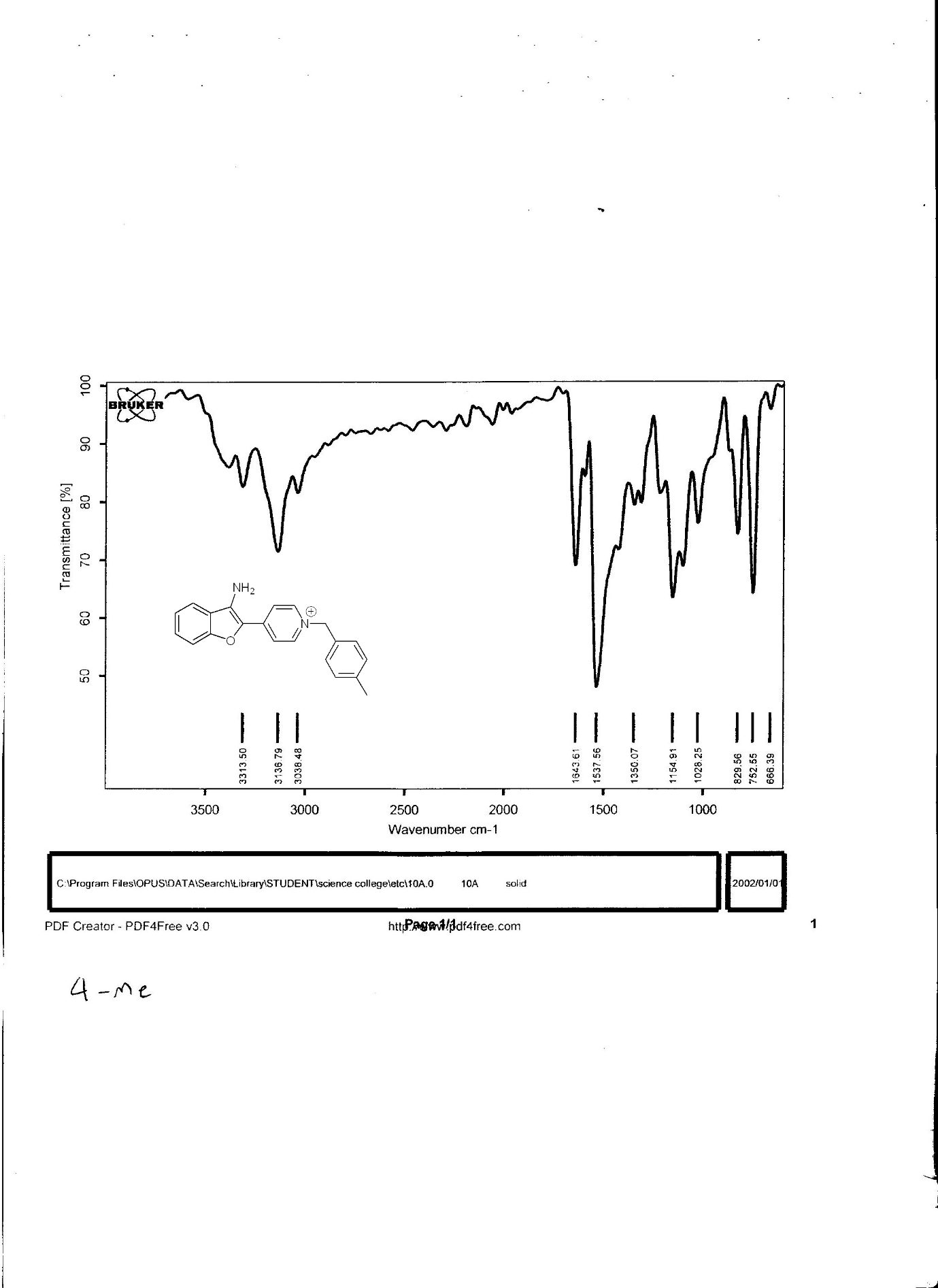
**

Figure S7. IR spectrum of compound **5d**


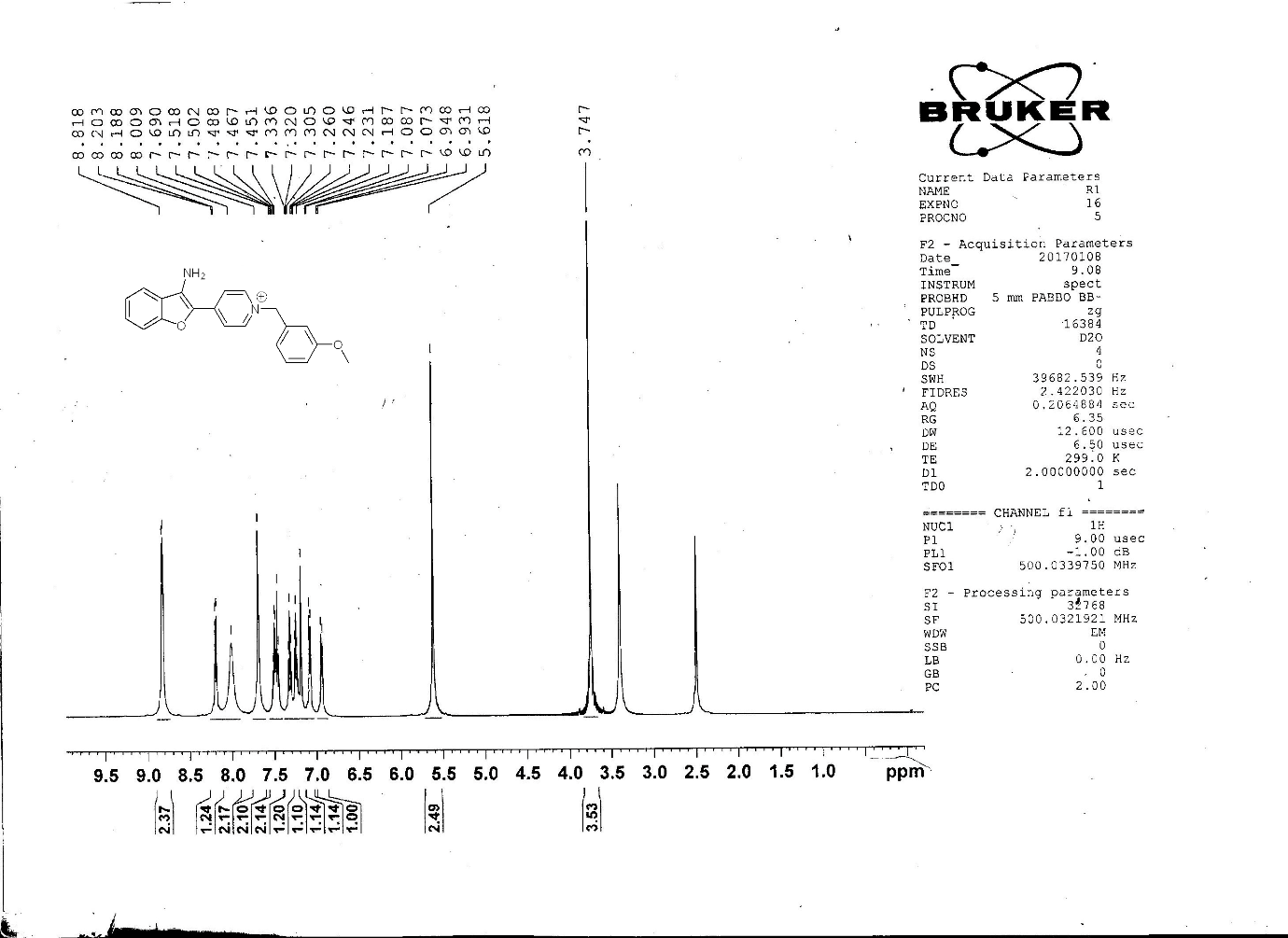


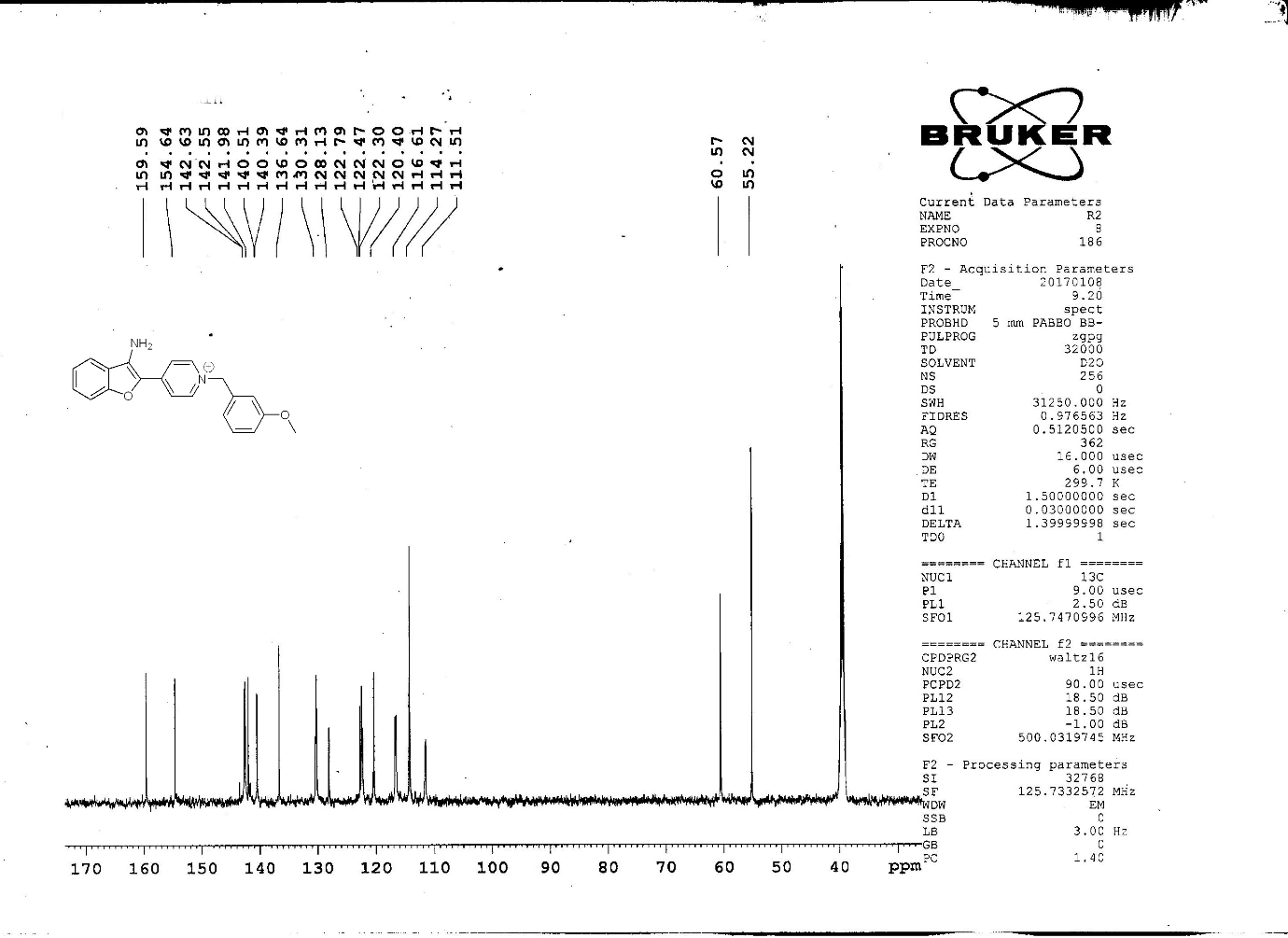


Figure S8. ^1^HNMR and ^13^CNMR spectra of compound **5e**


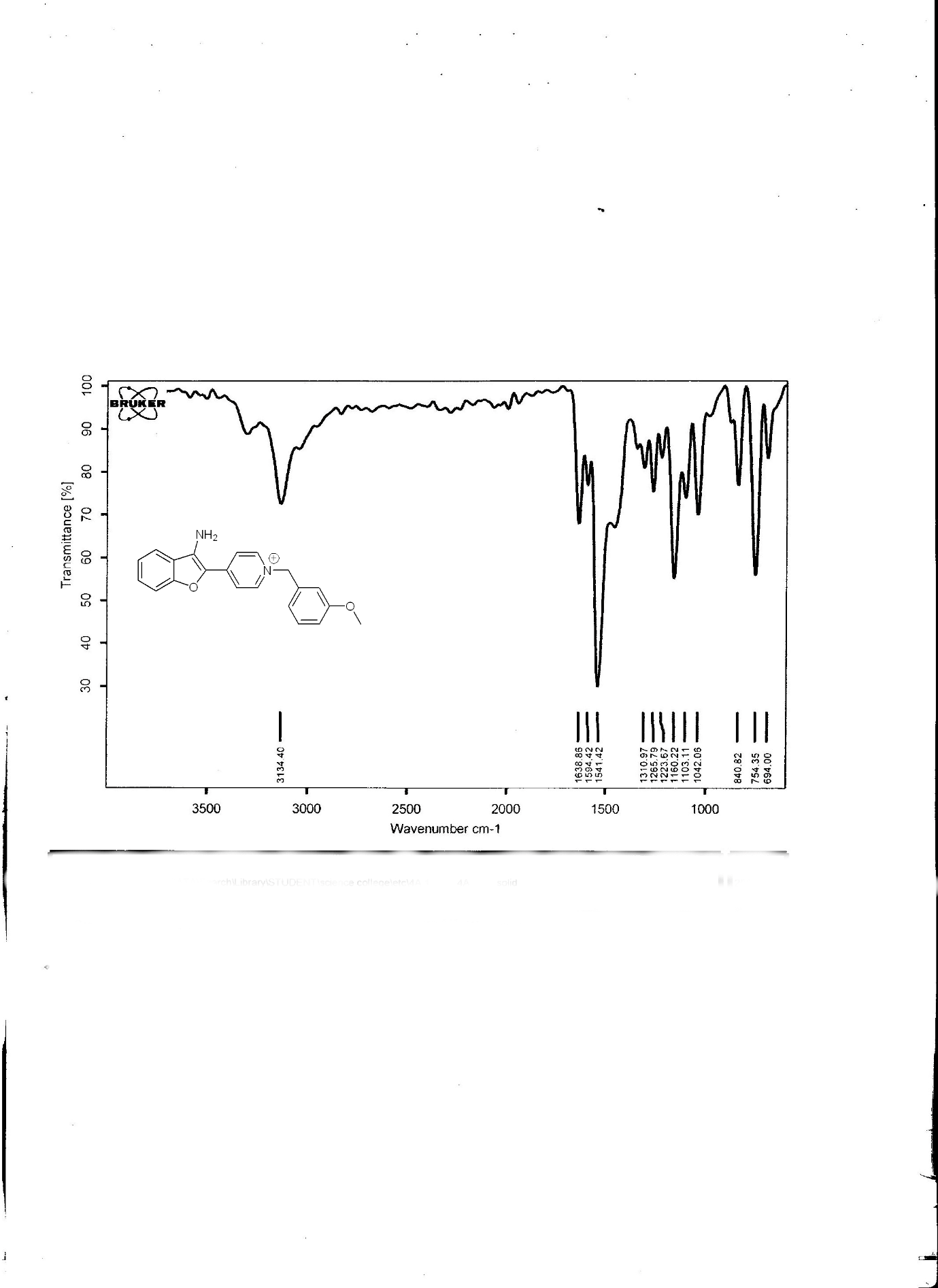


Figure S9. IR spectrum of compound **5e**

**
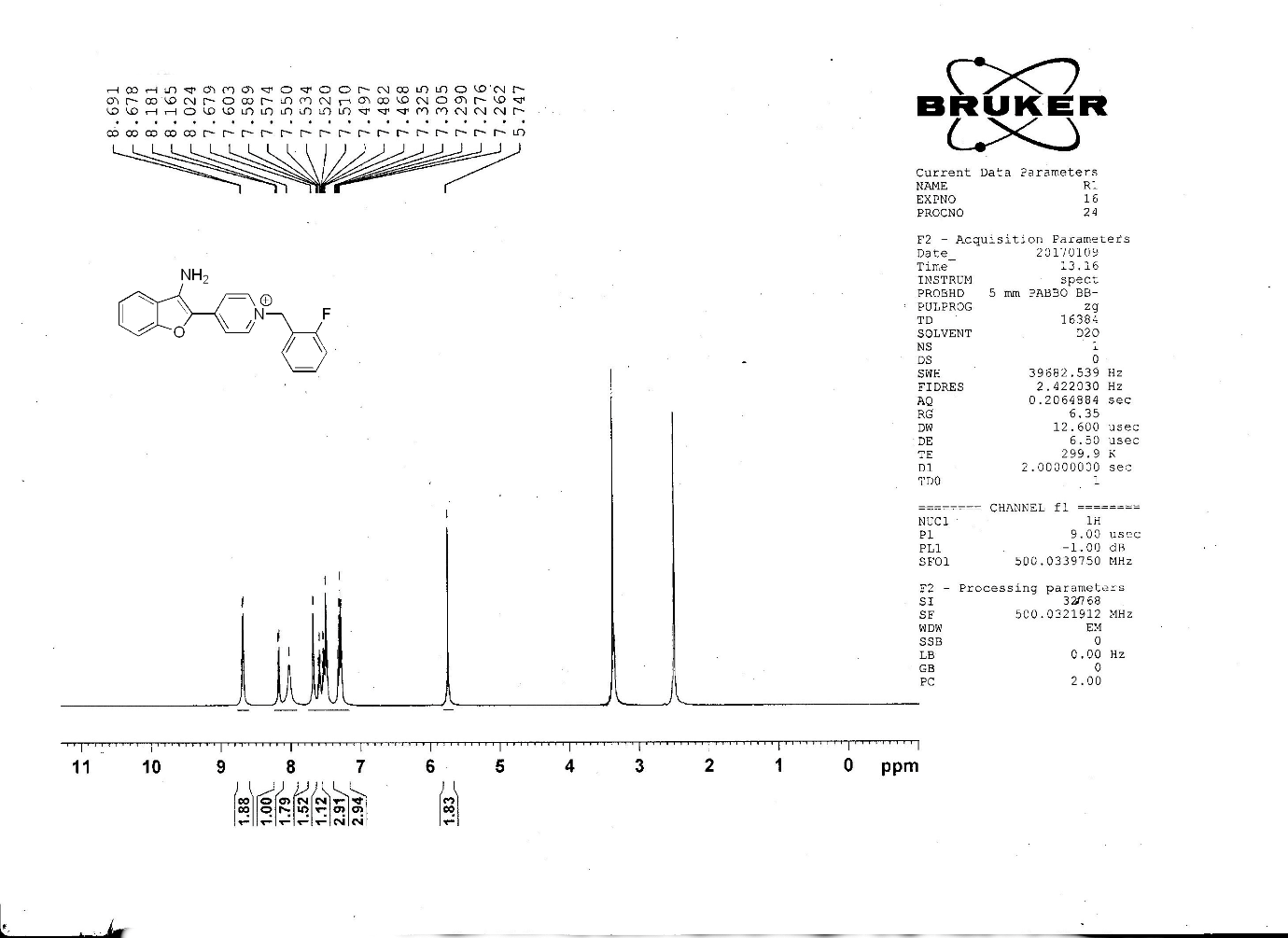
**

**
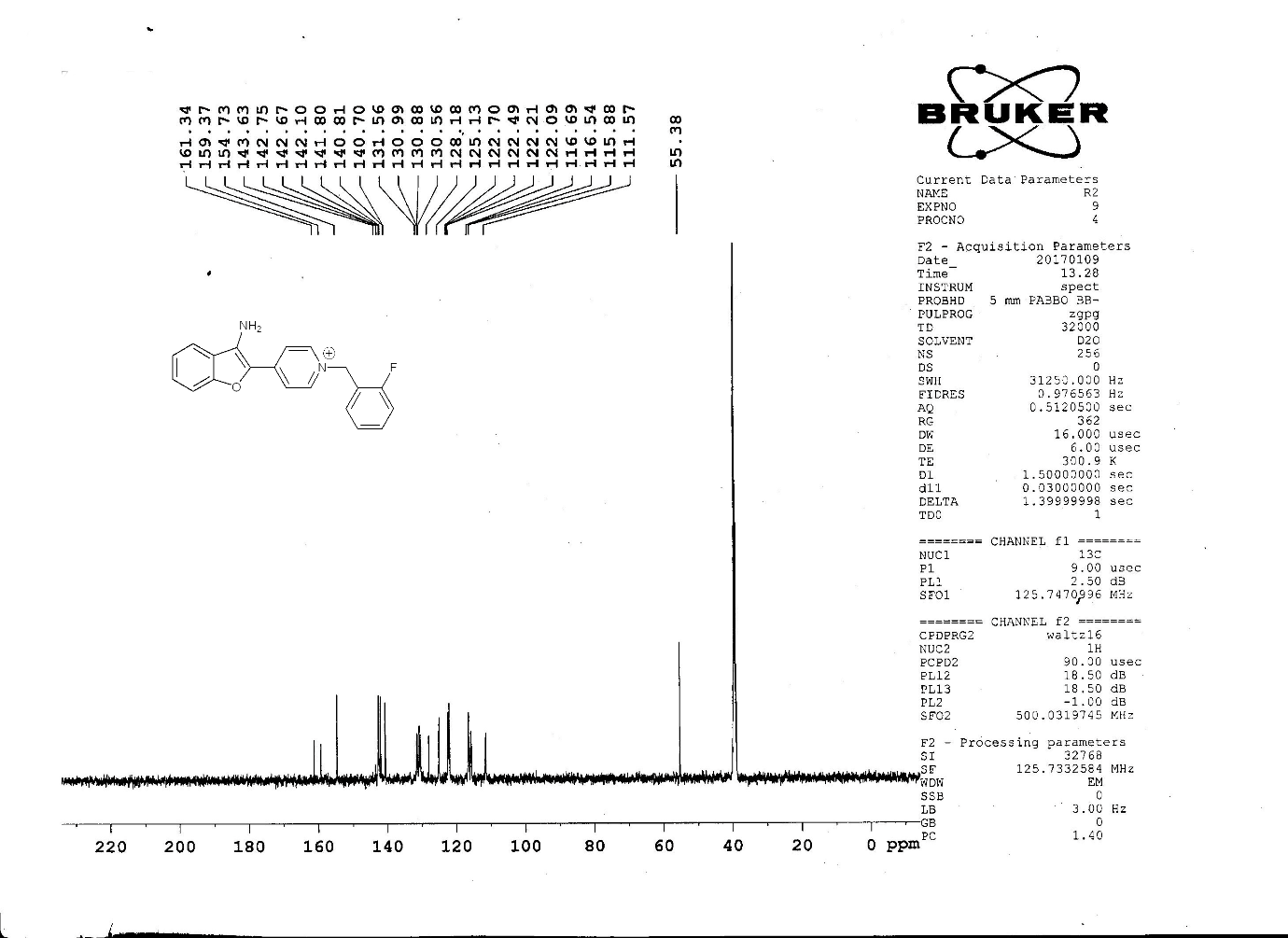
**

Figure S10. ^1^HNMR and ^13^CNMR spectra of compound **5f**

**
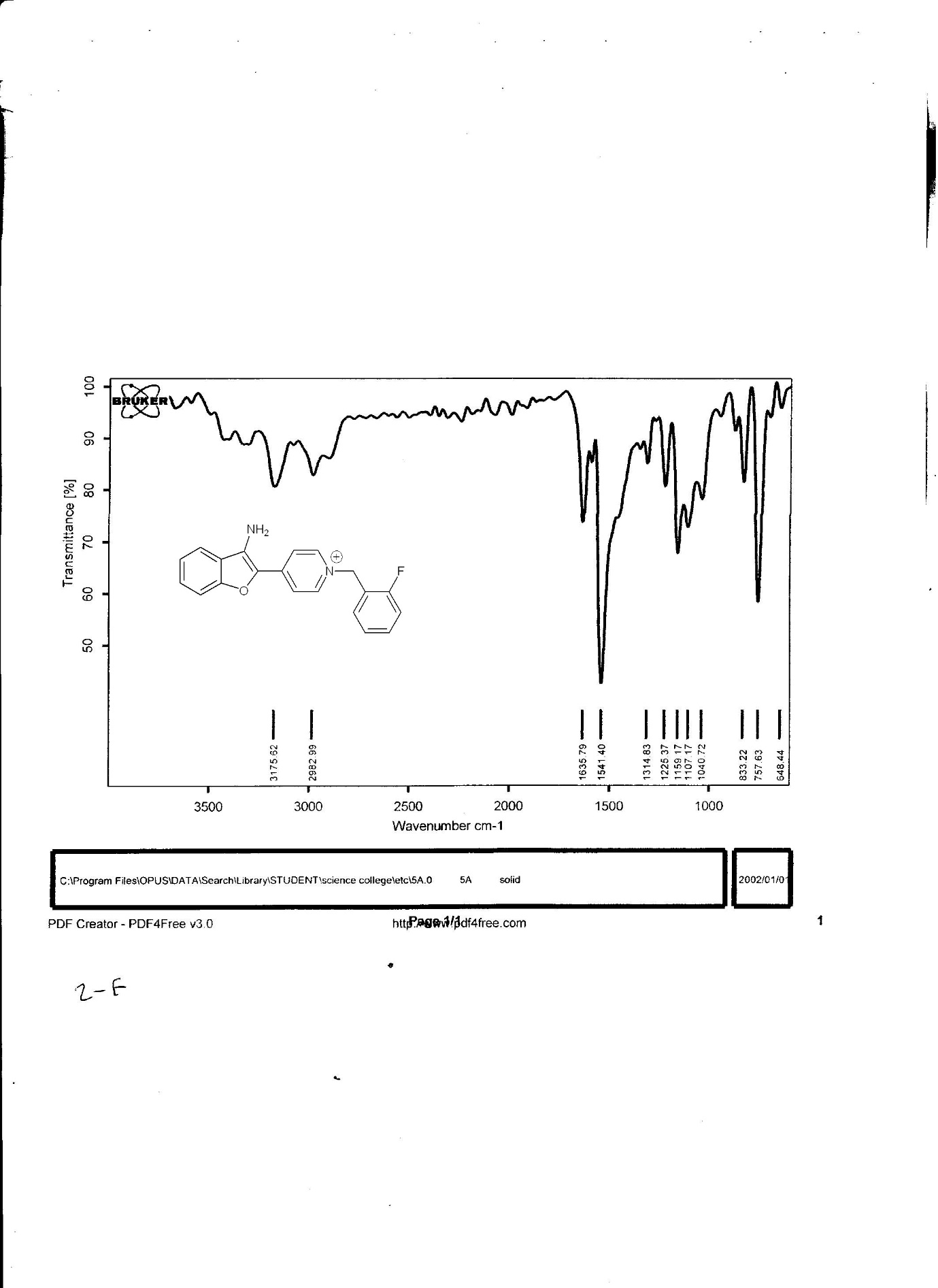
**

Figure S11. IR spectrum of compound **5f**

**
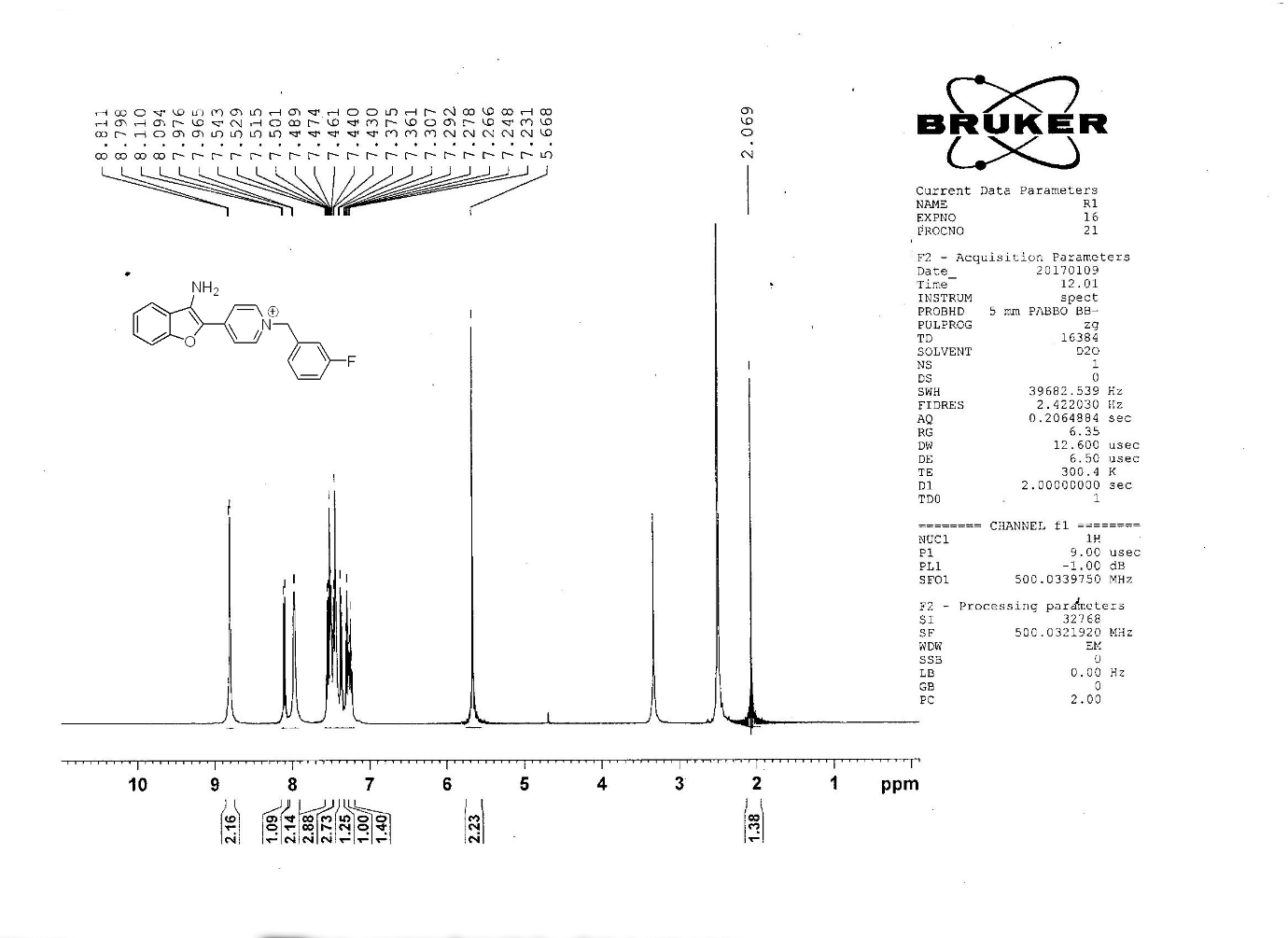

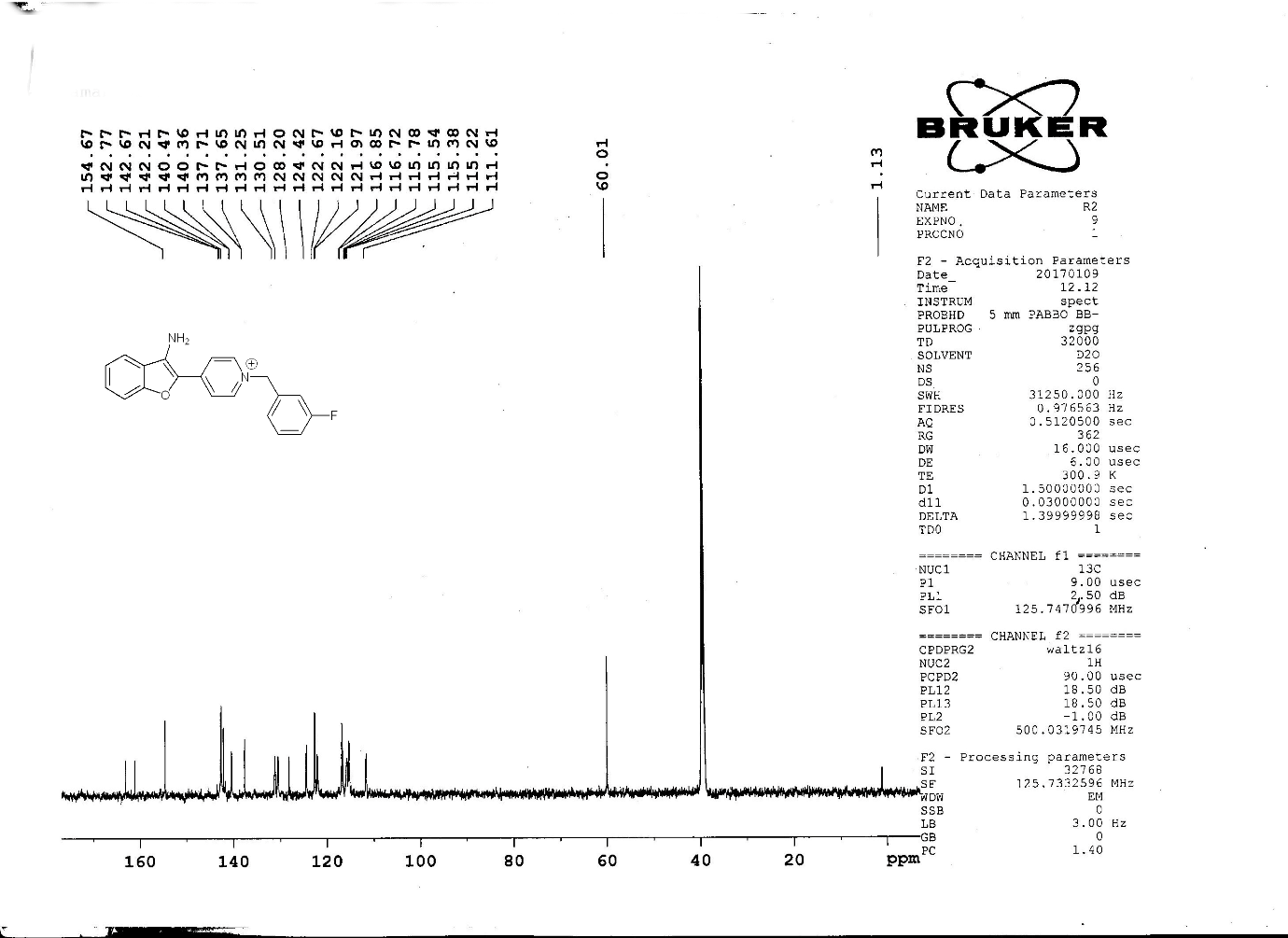
**

Figure S12. ^1^HNMR and ^13^CNMR spectra of compound **5g**

**
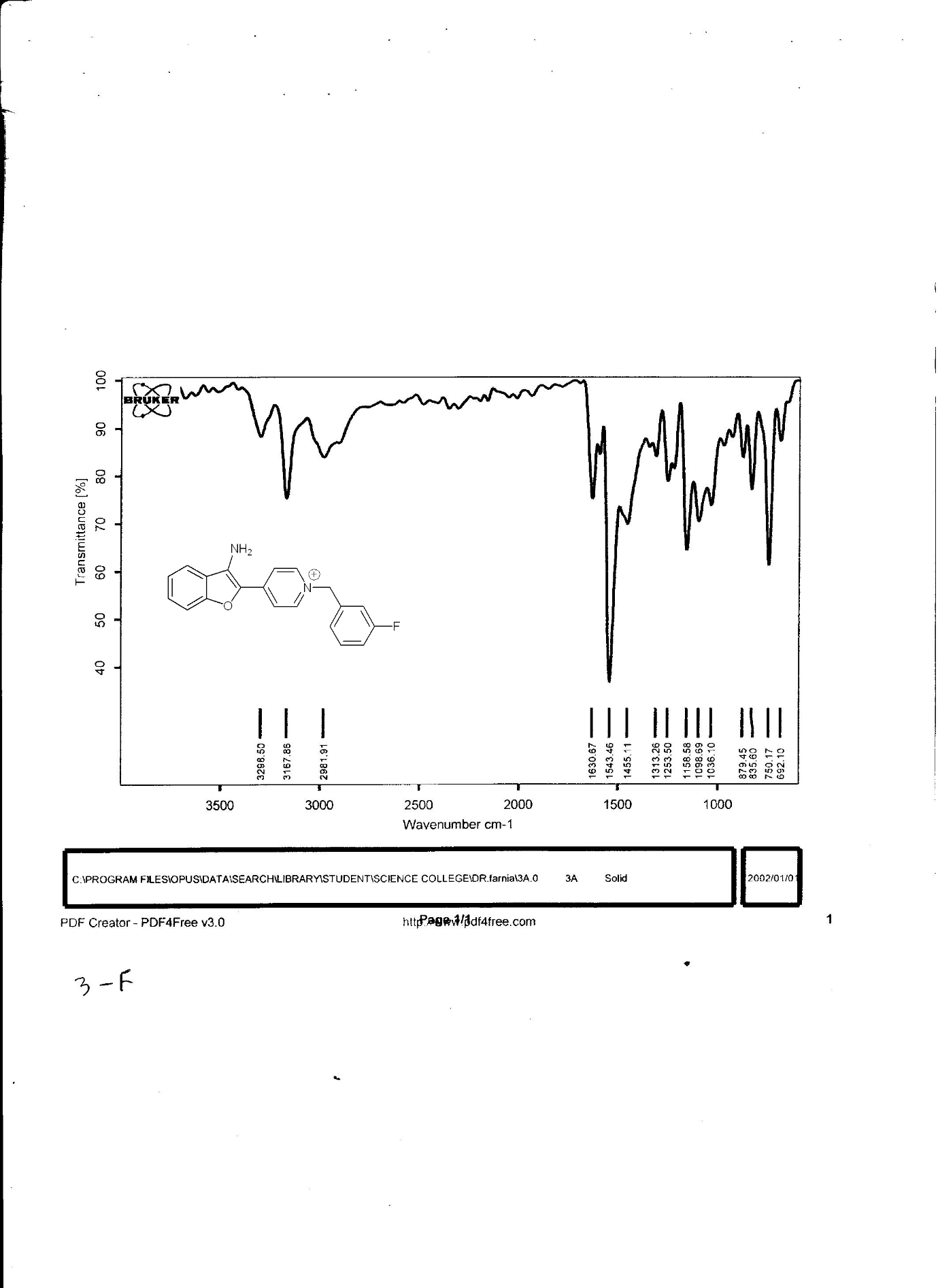
**

Figure S13. IR spectrum of compound **5g**

**
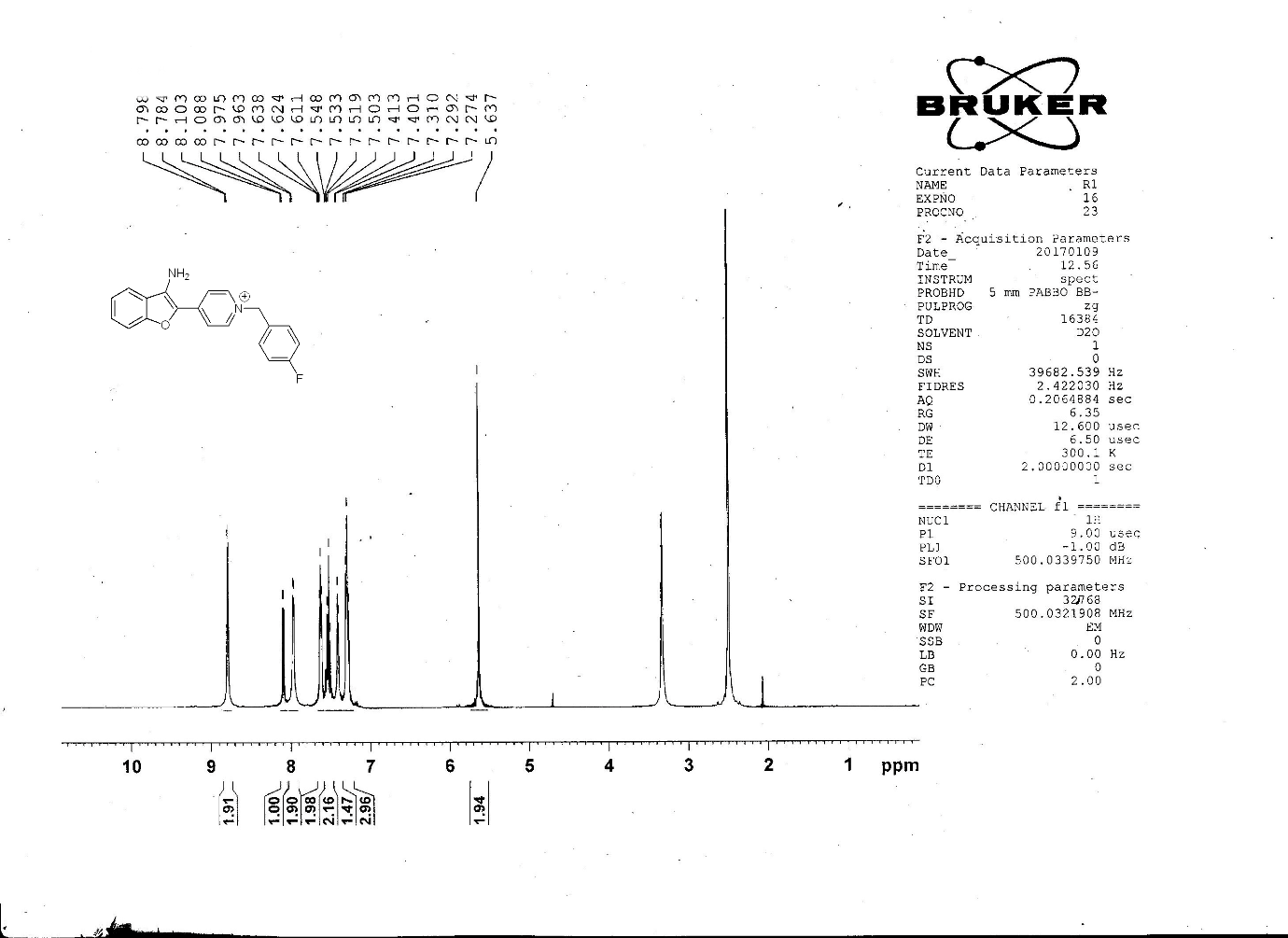
**

**
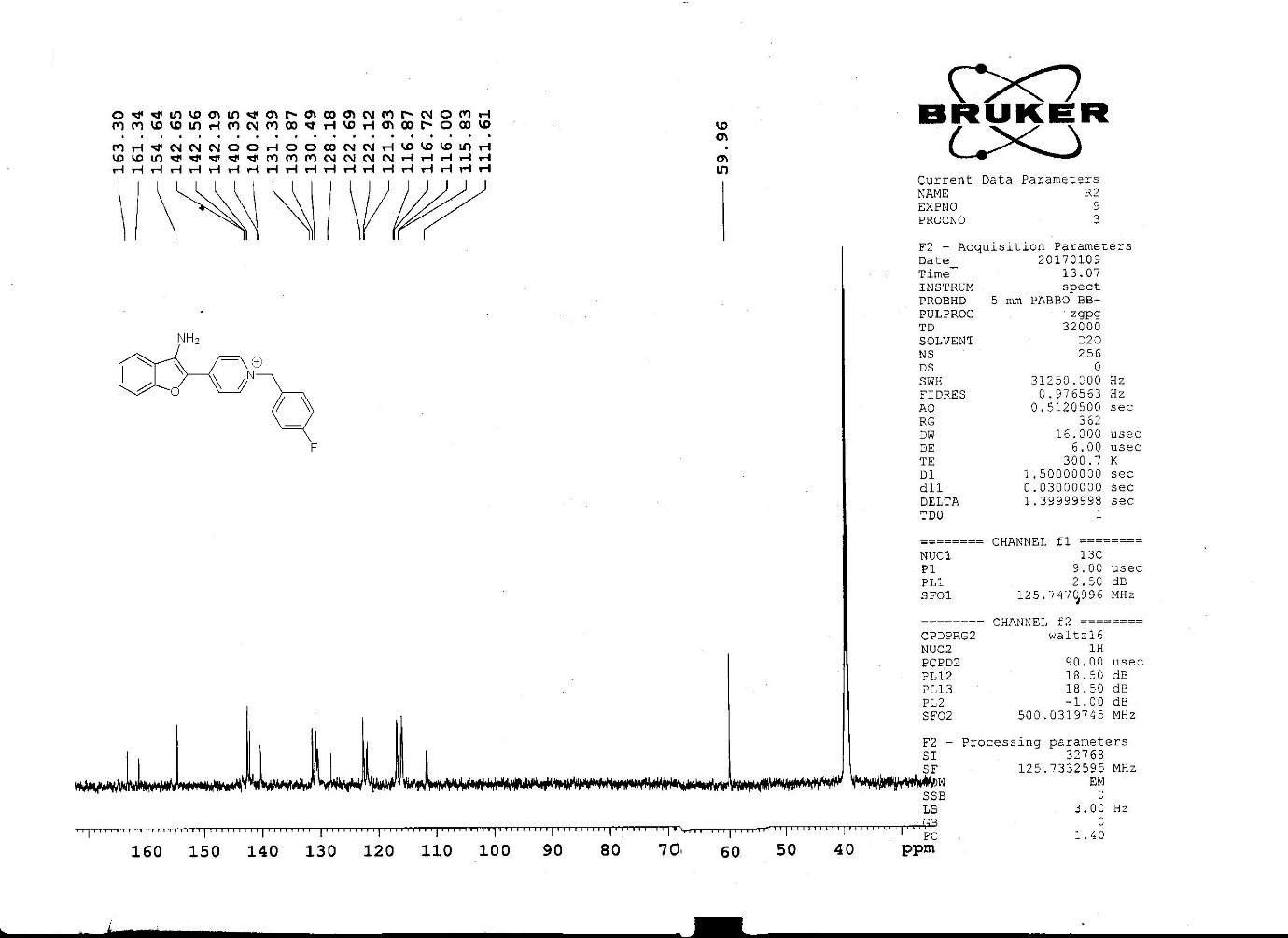
**

Figure S14. ^1^HNMR and ^13^CNMR spectra of compound **5h**


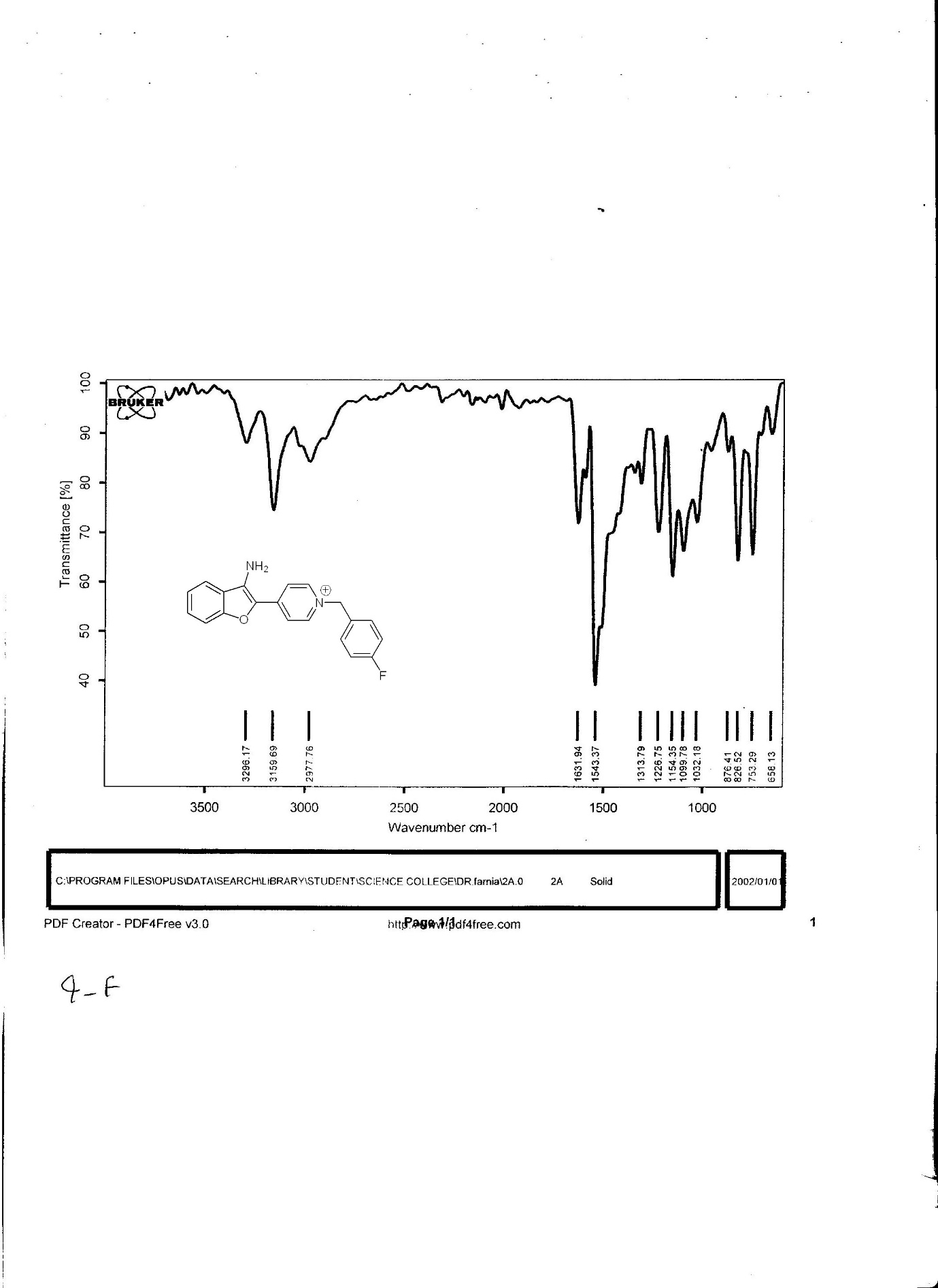


Figure S15. IR spectrum of compound **5h**

**
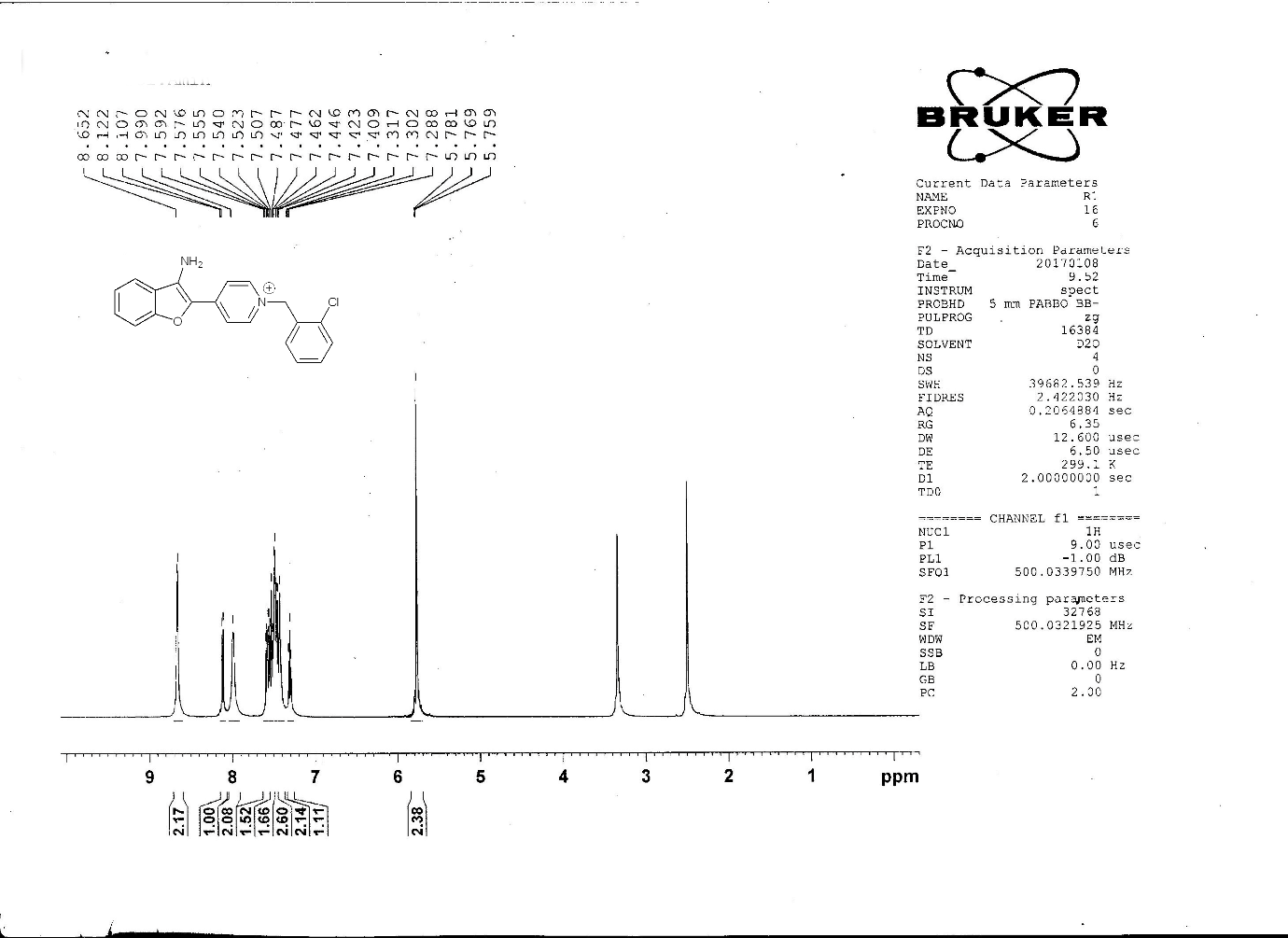
**


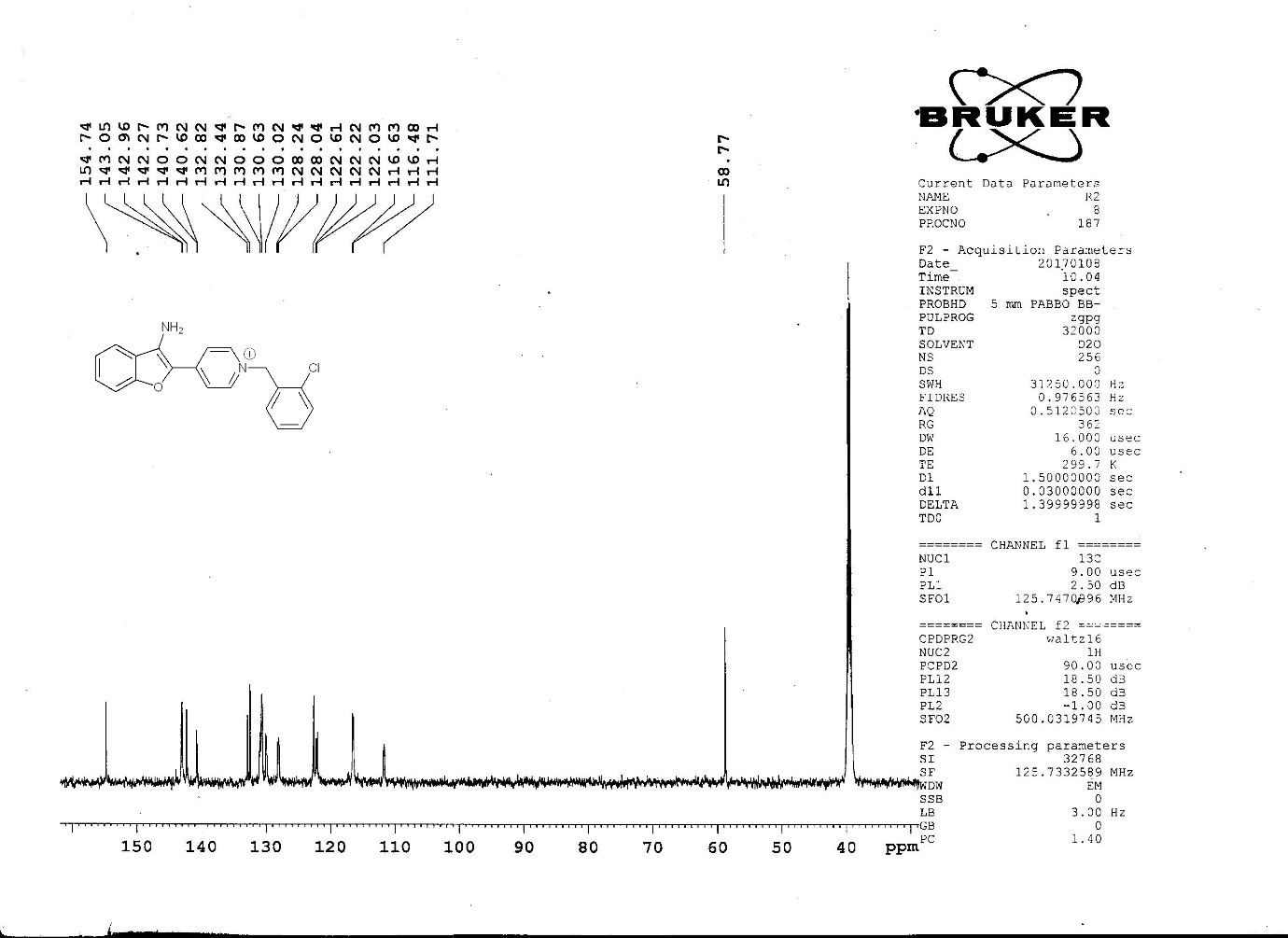


Figure S16. ^1^HNMR and ^13^CNMR spectra of compound **5i**

**
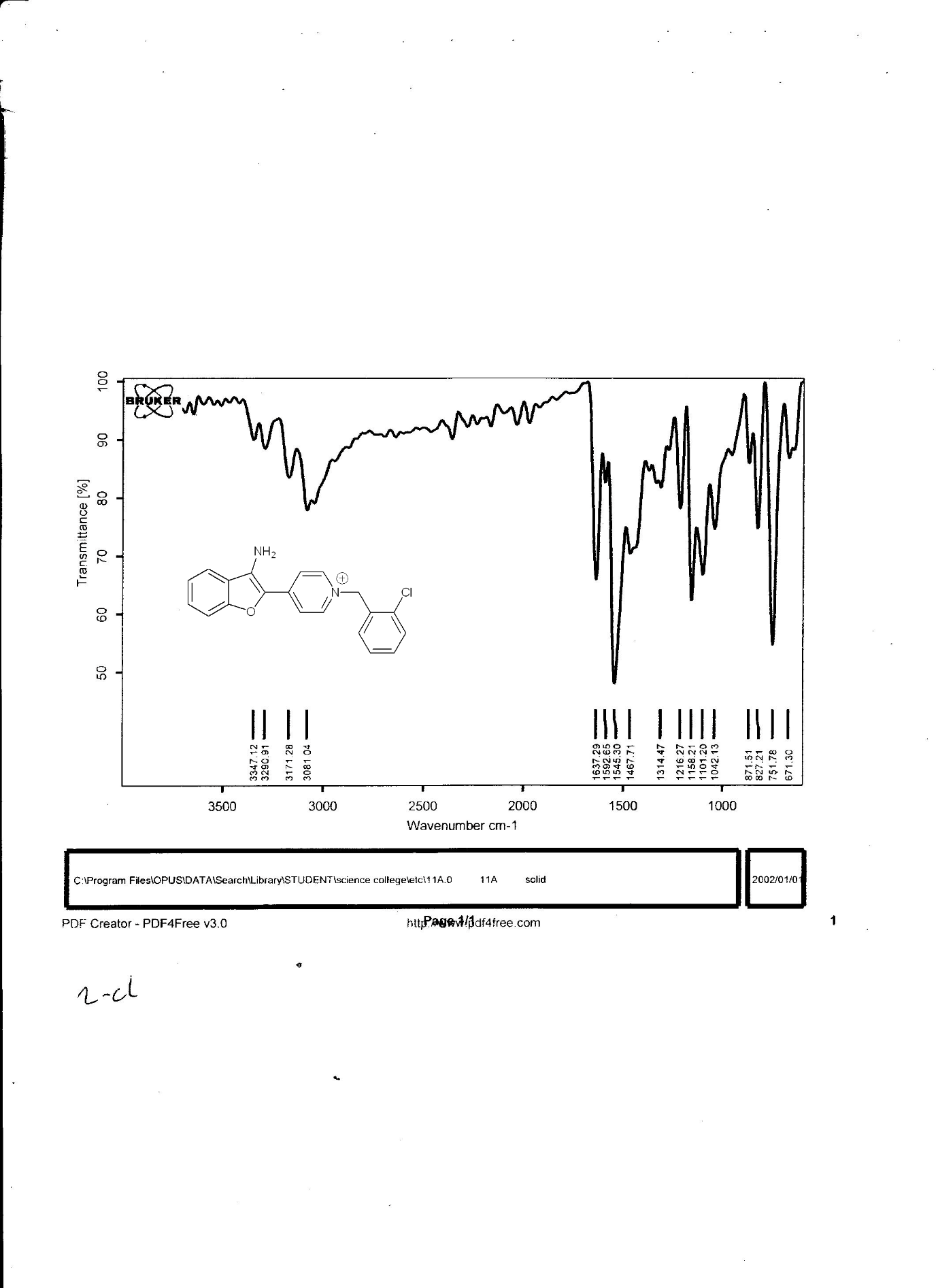
**

Figure S17. IR spectrum of compound **5i**

**
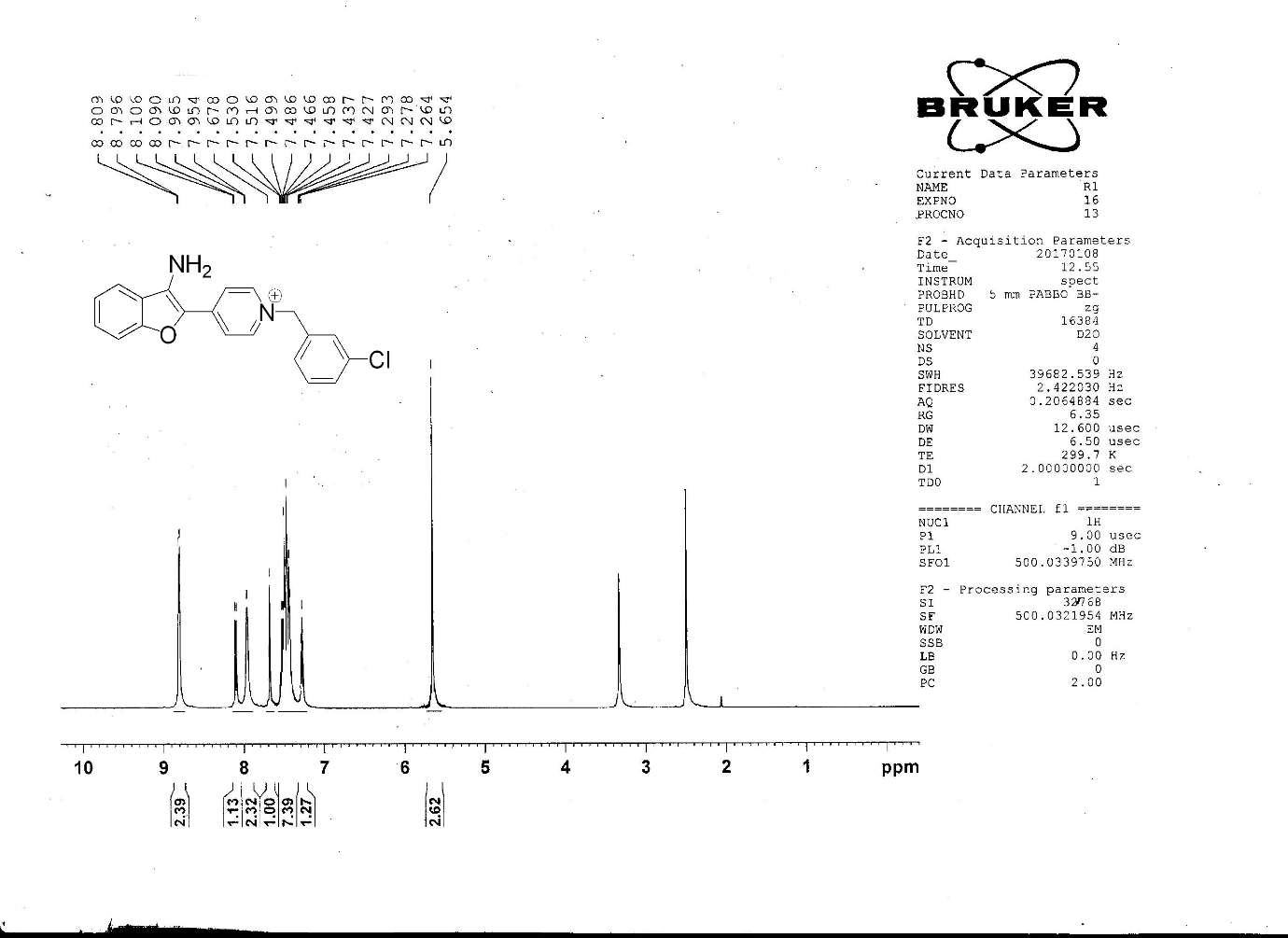
**


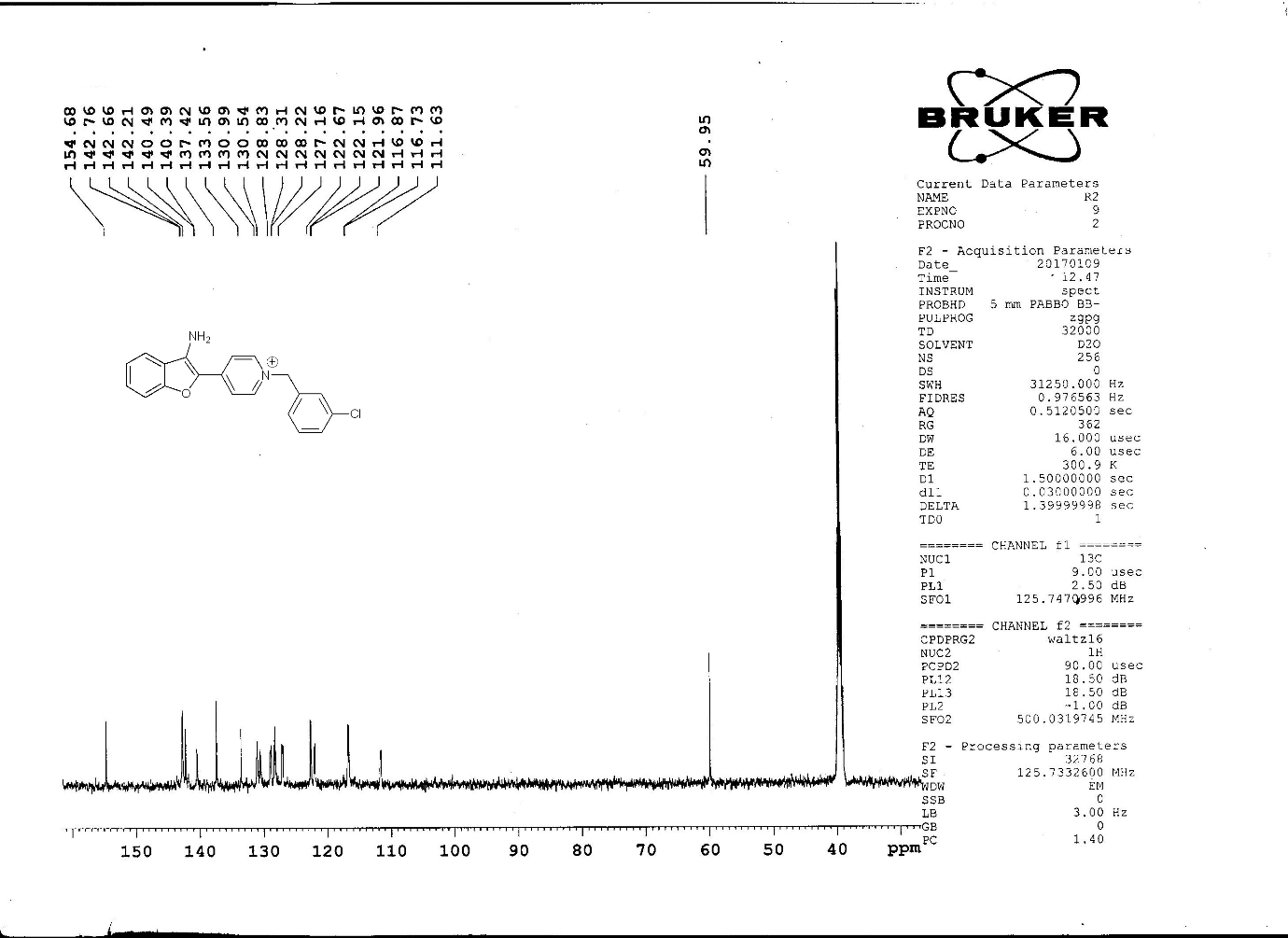


Figure S18. ^1^HNMR and ^13^CNMR spectra of compound **5j**

**
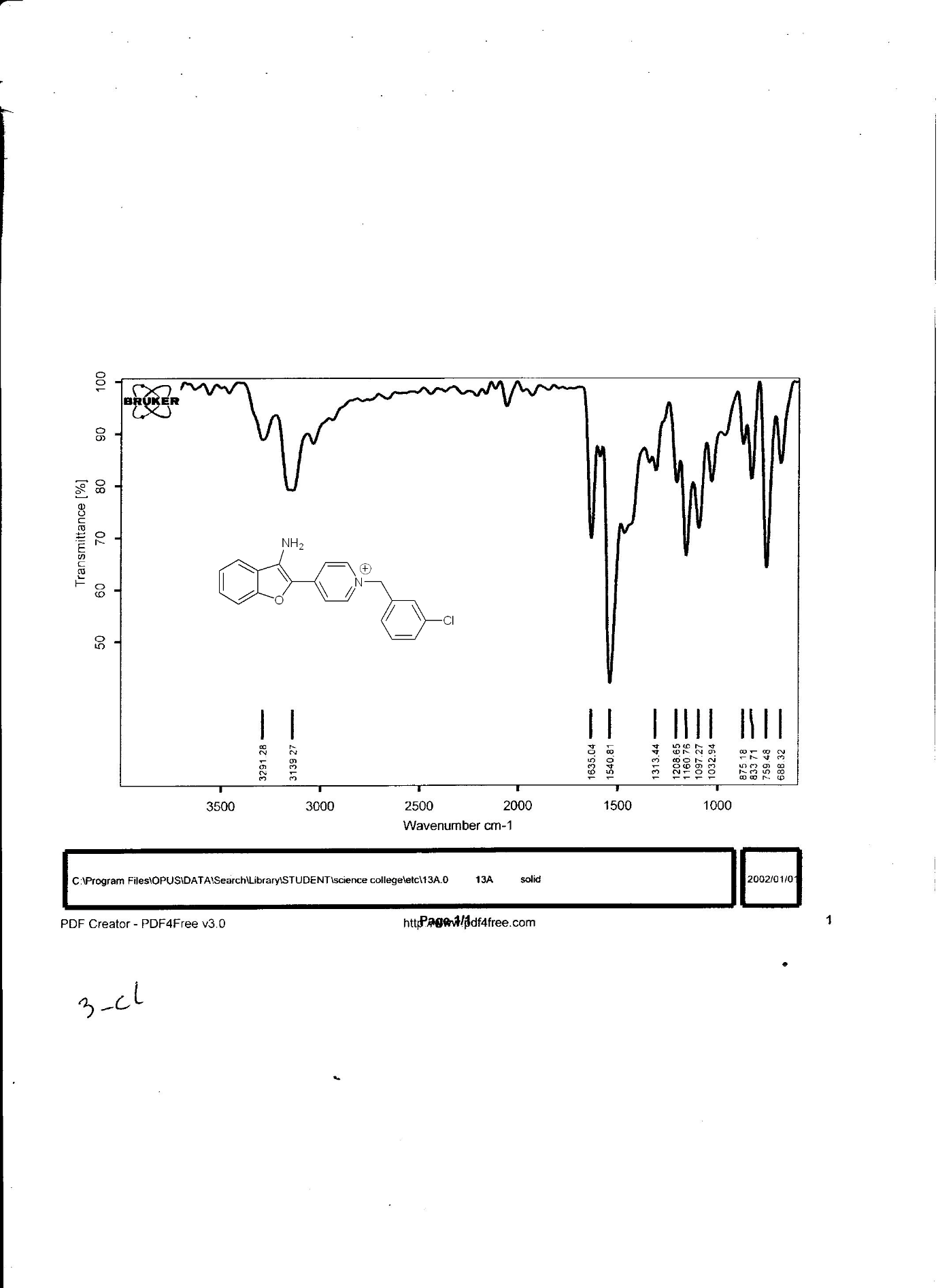
**

Figure S19. IR spectrum of compound **5j**

**
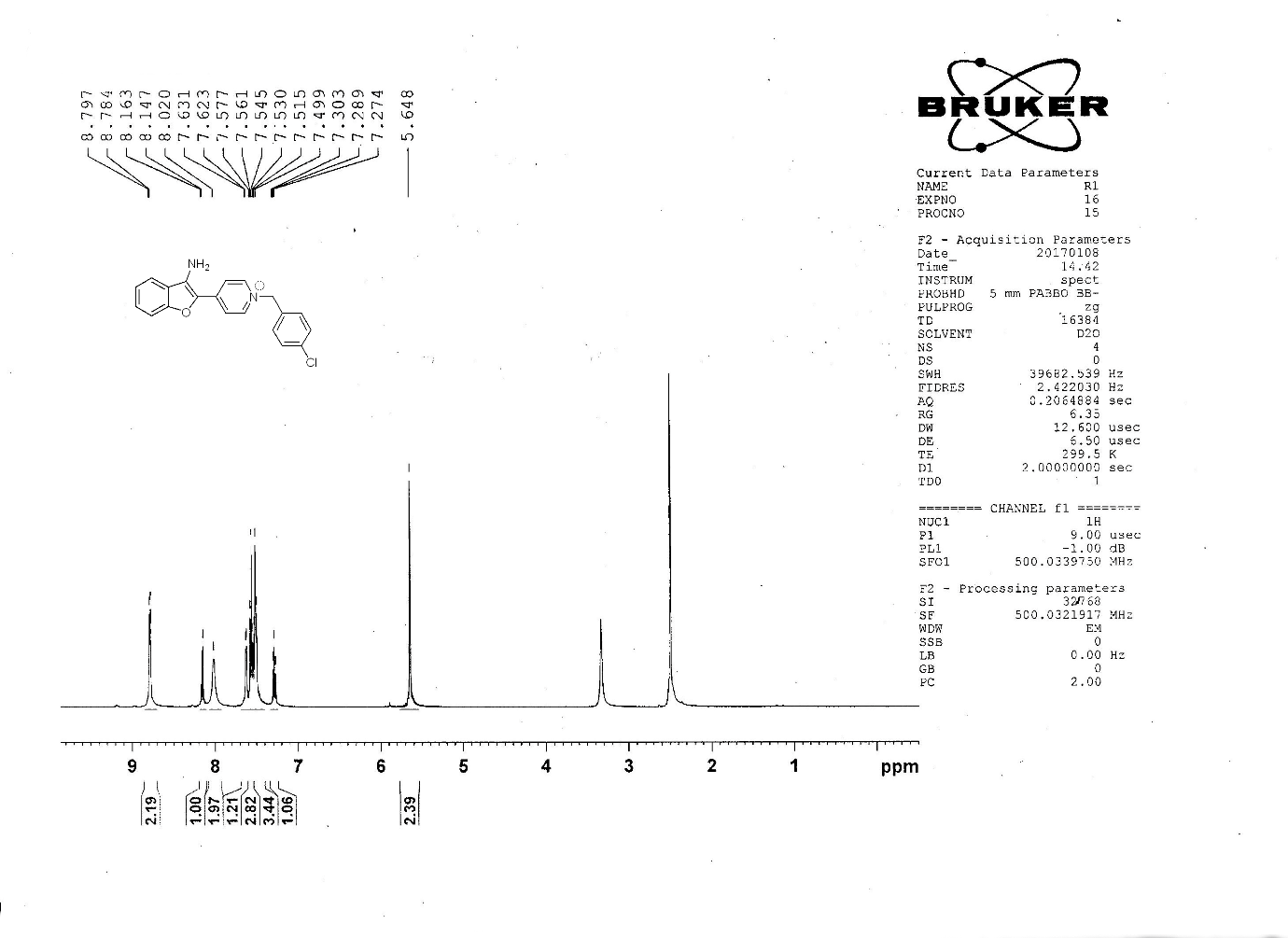
**

**
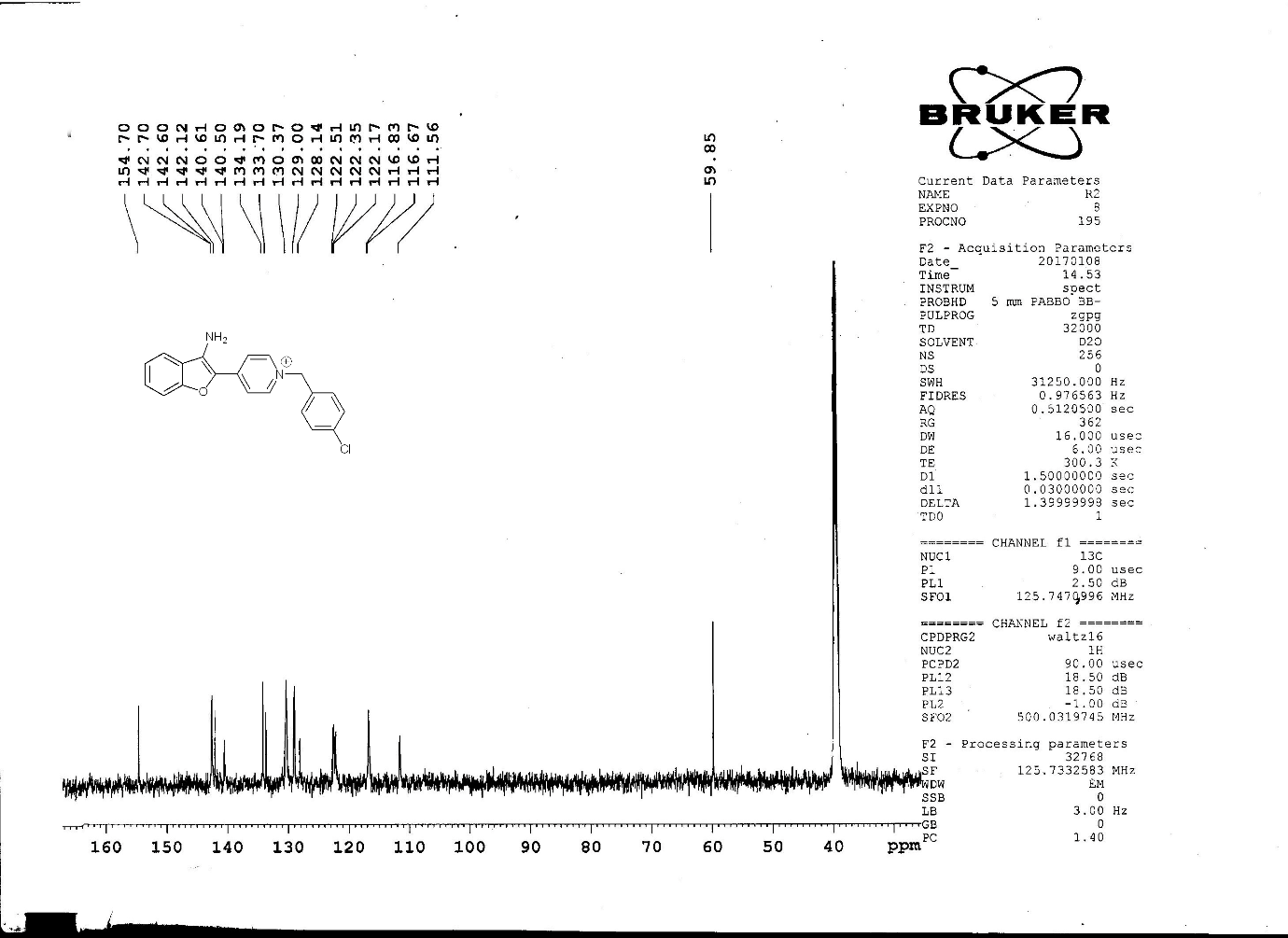
**

Figure S20. ^1^HNMR and ^13^CNMR spectra of compound **5k**


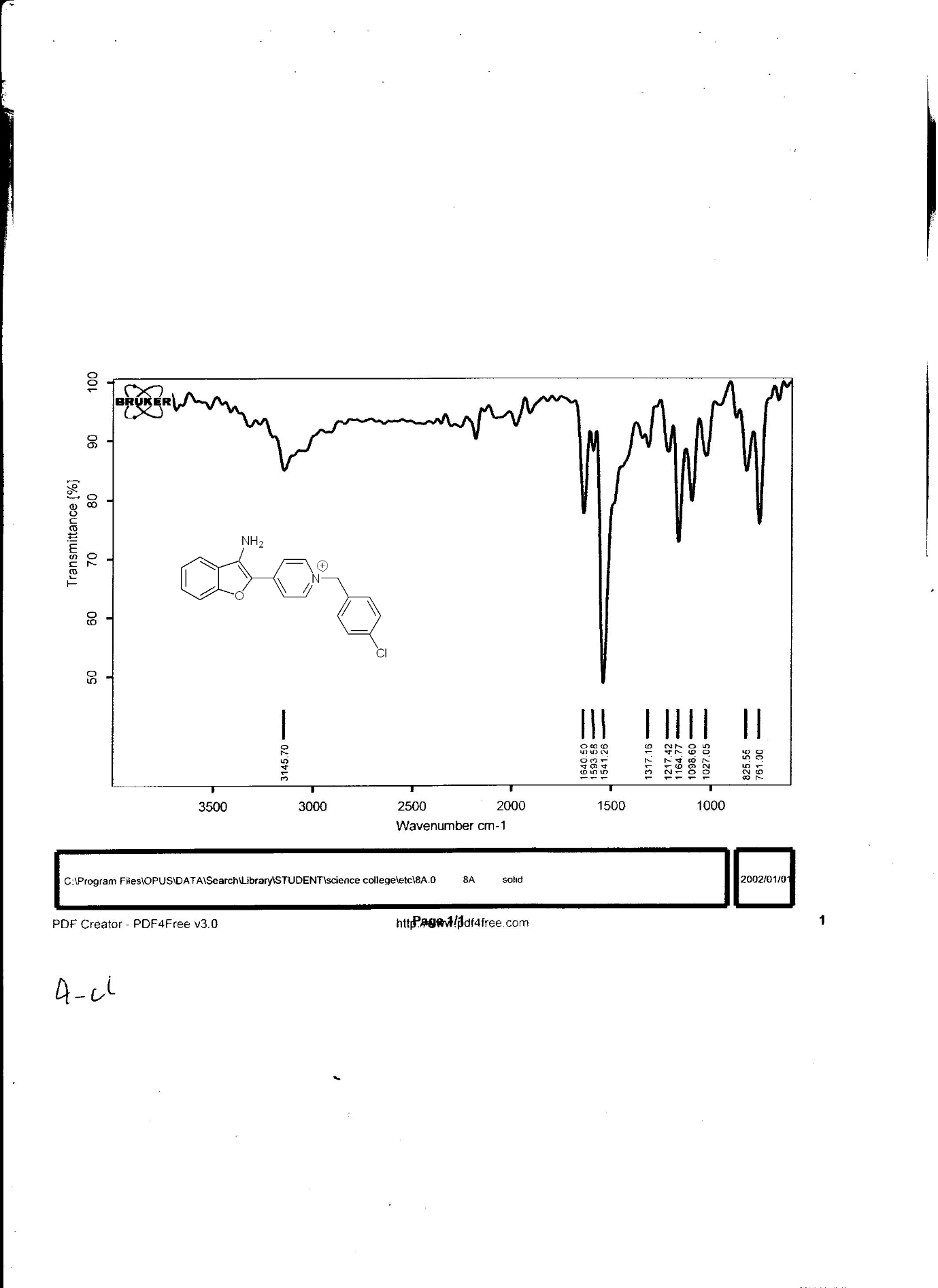


Figure S21. IR spectrum of compound **5k**


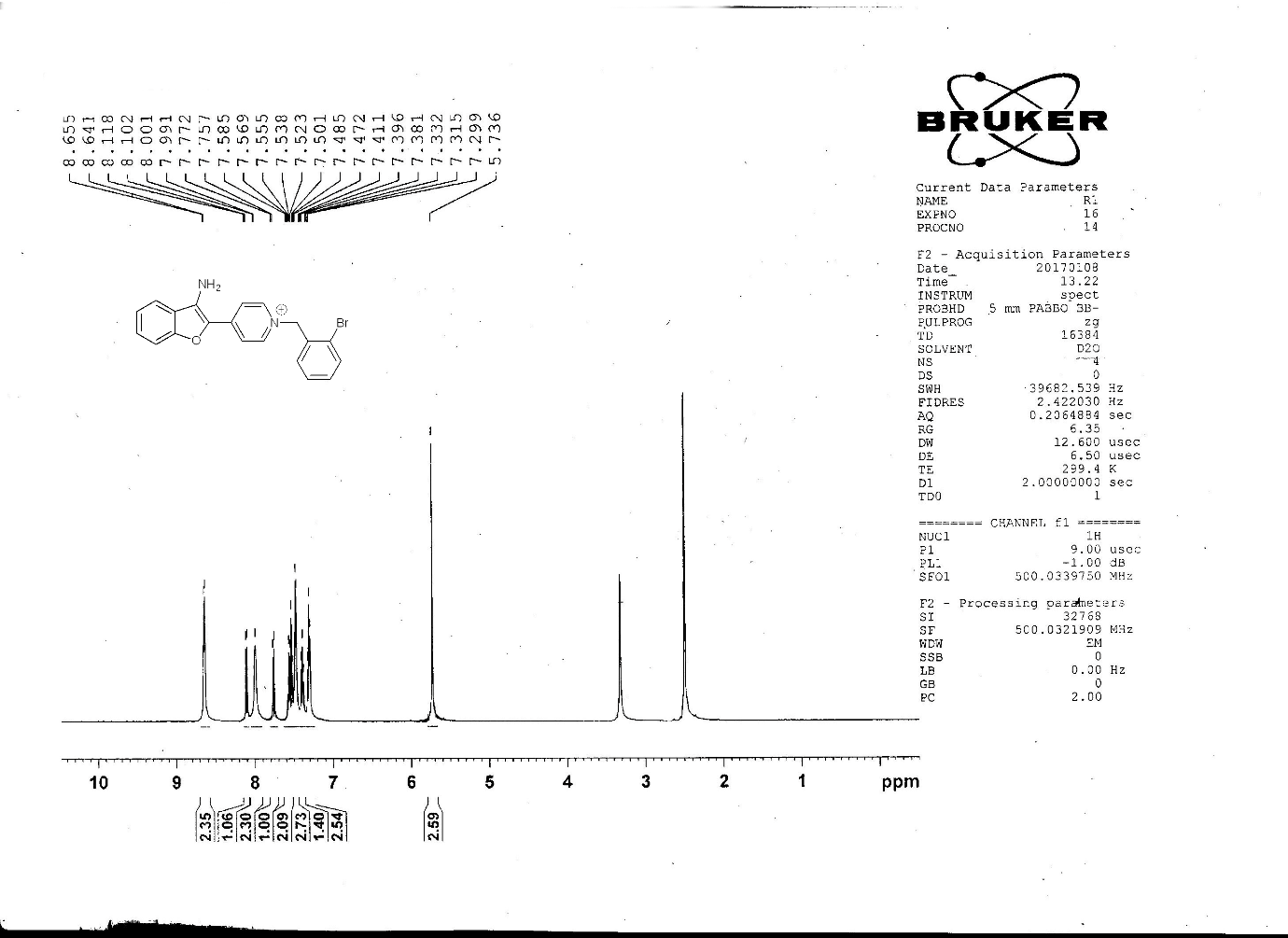

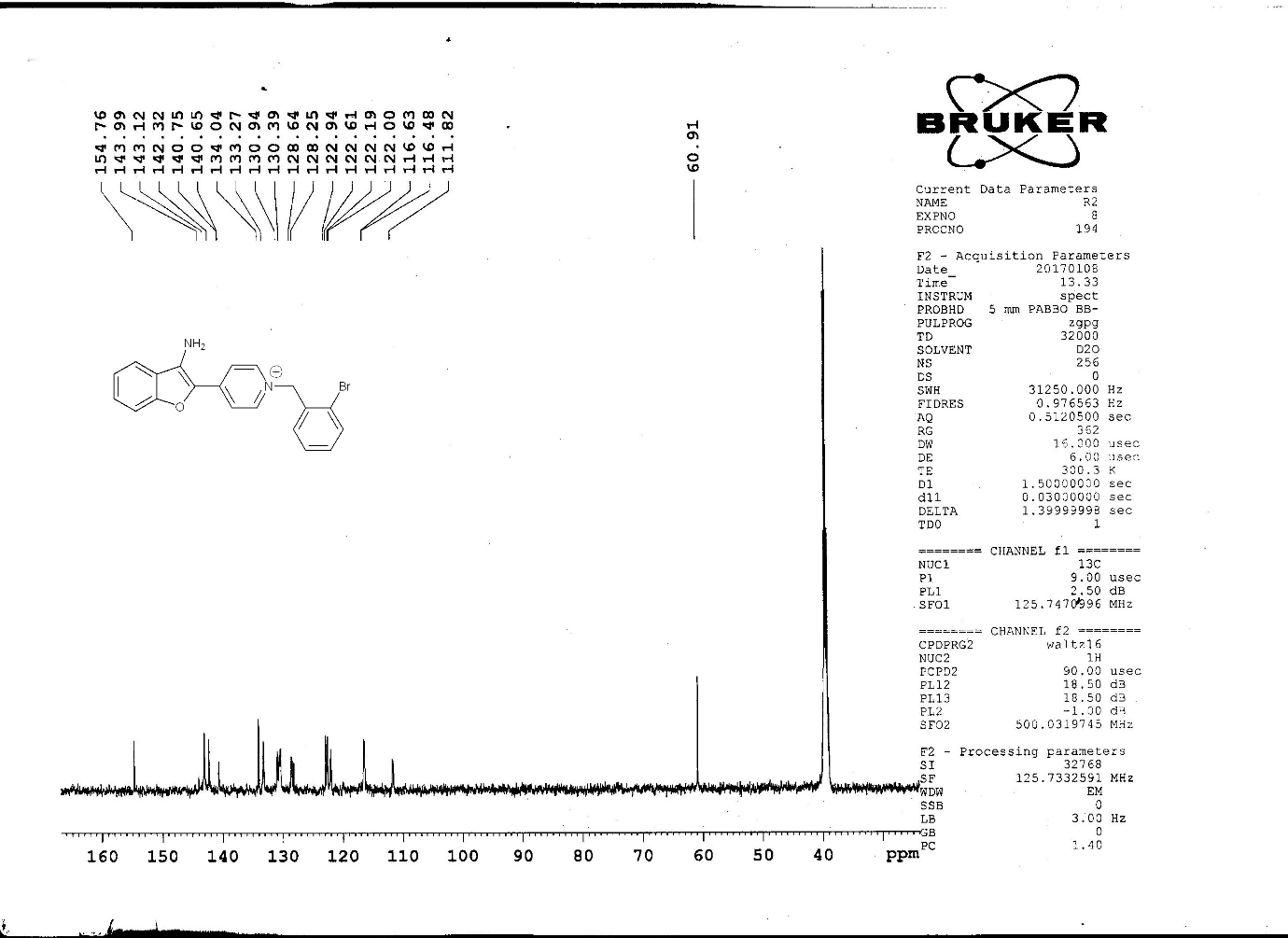


Figure S22. ^1^HNMR and ^13^CNMR spectra of compound **5l**

**
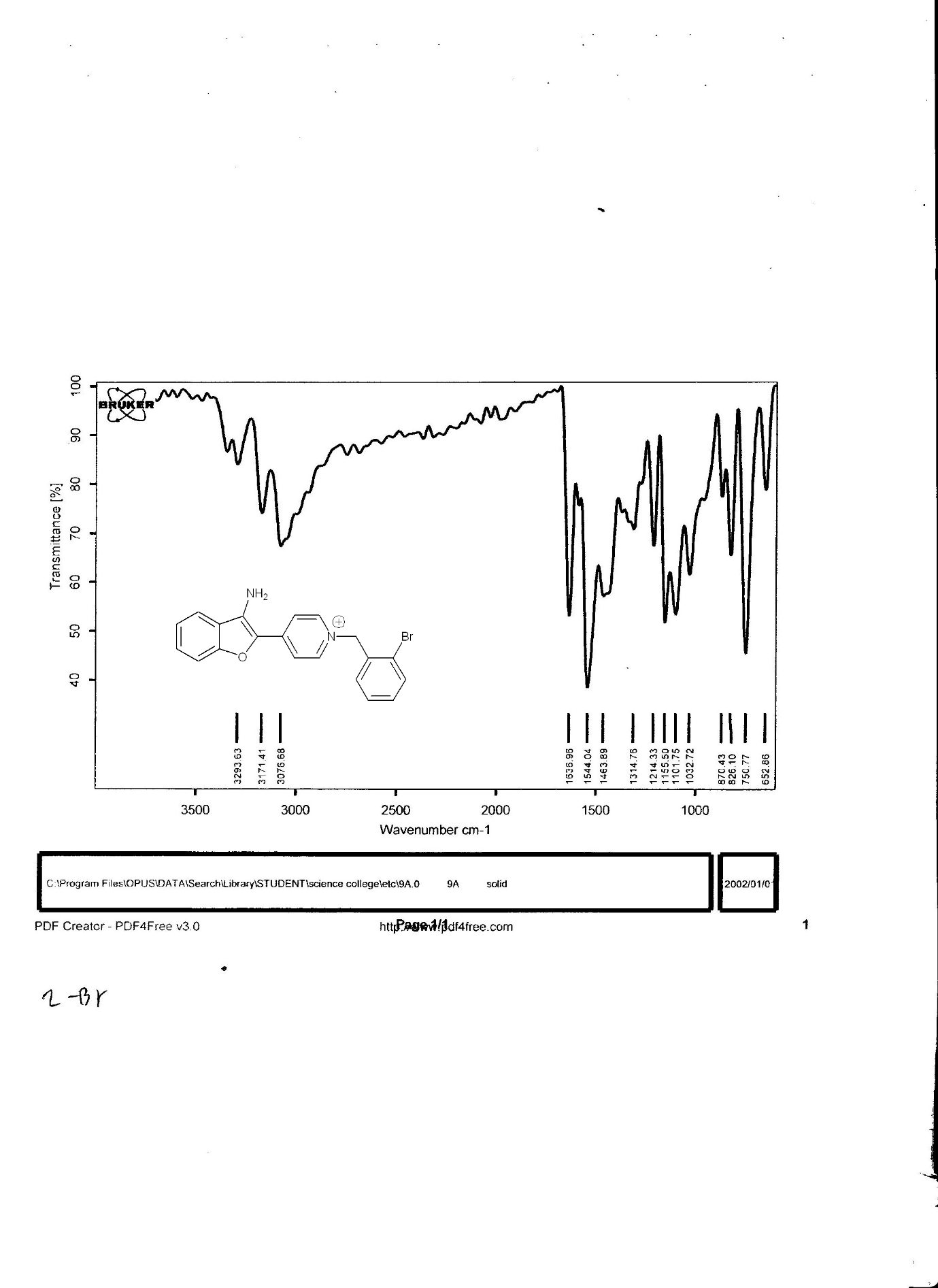
**

Figure S23. IR spectrum of compound **5l**

**
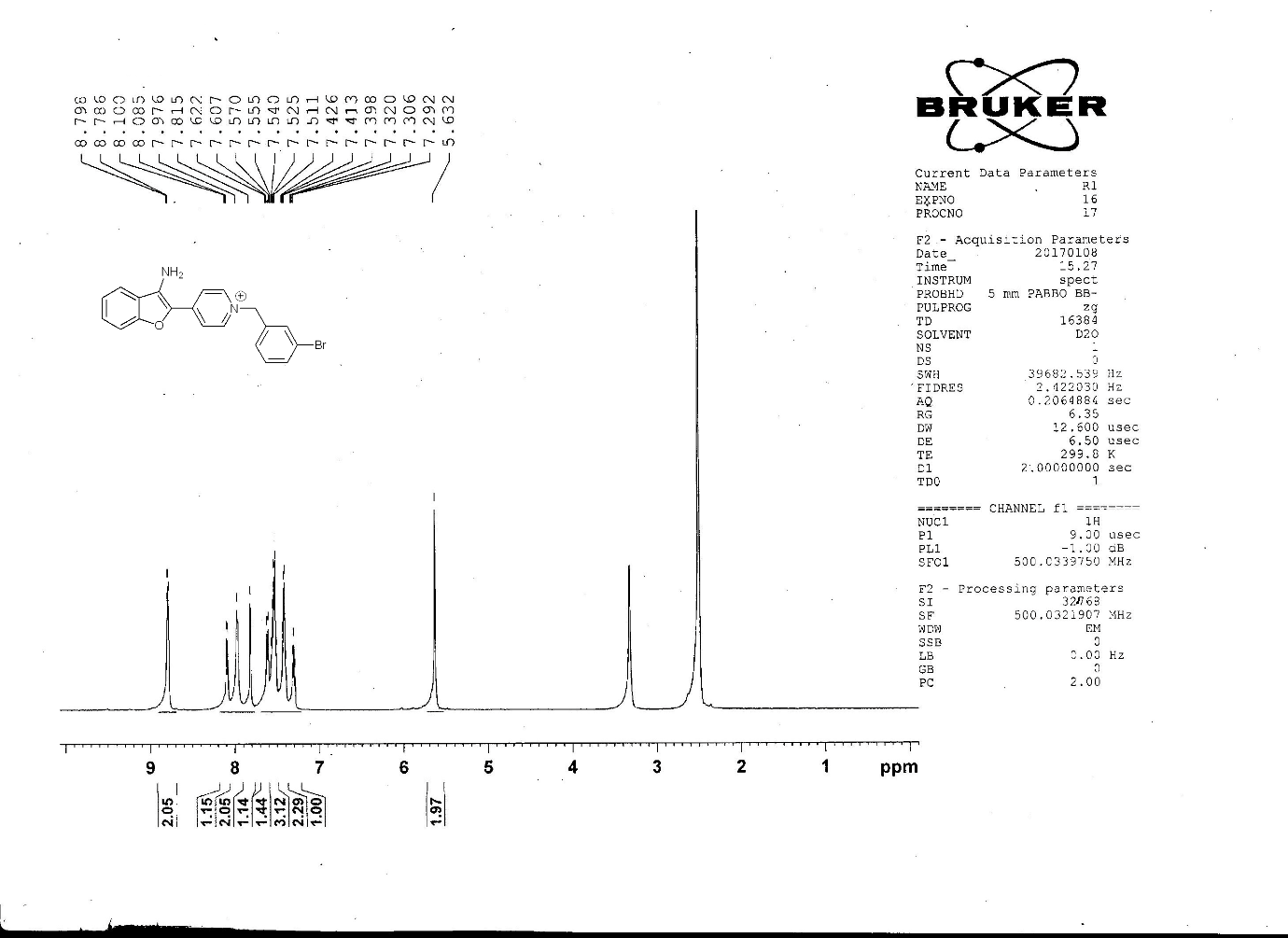
**

**
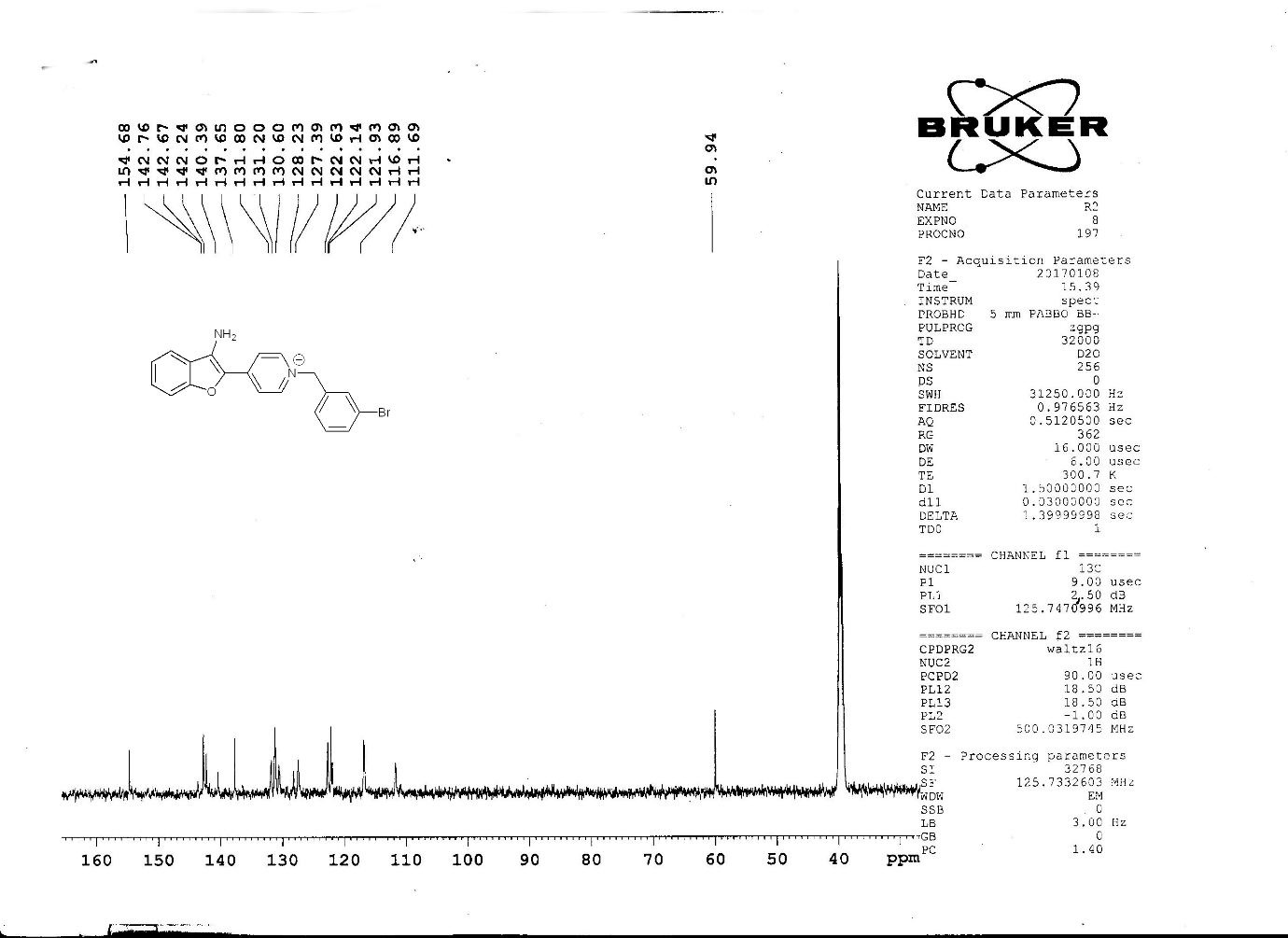
**

Figure S24. ^1^HNMR and ^13^CNMR spectra of compound **5m**


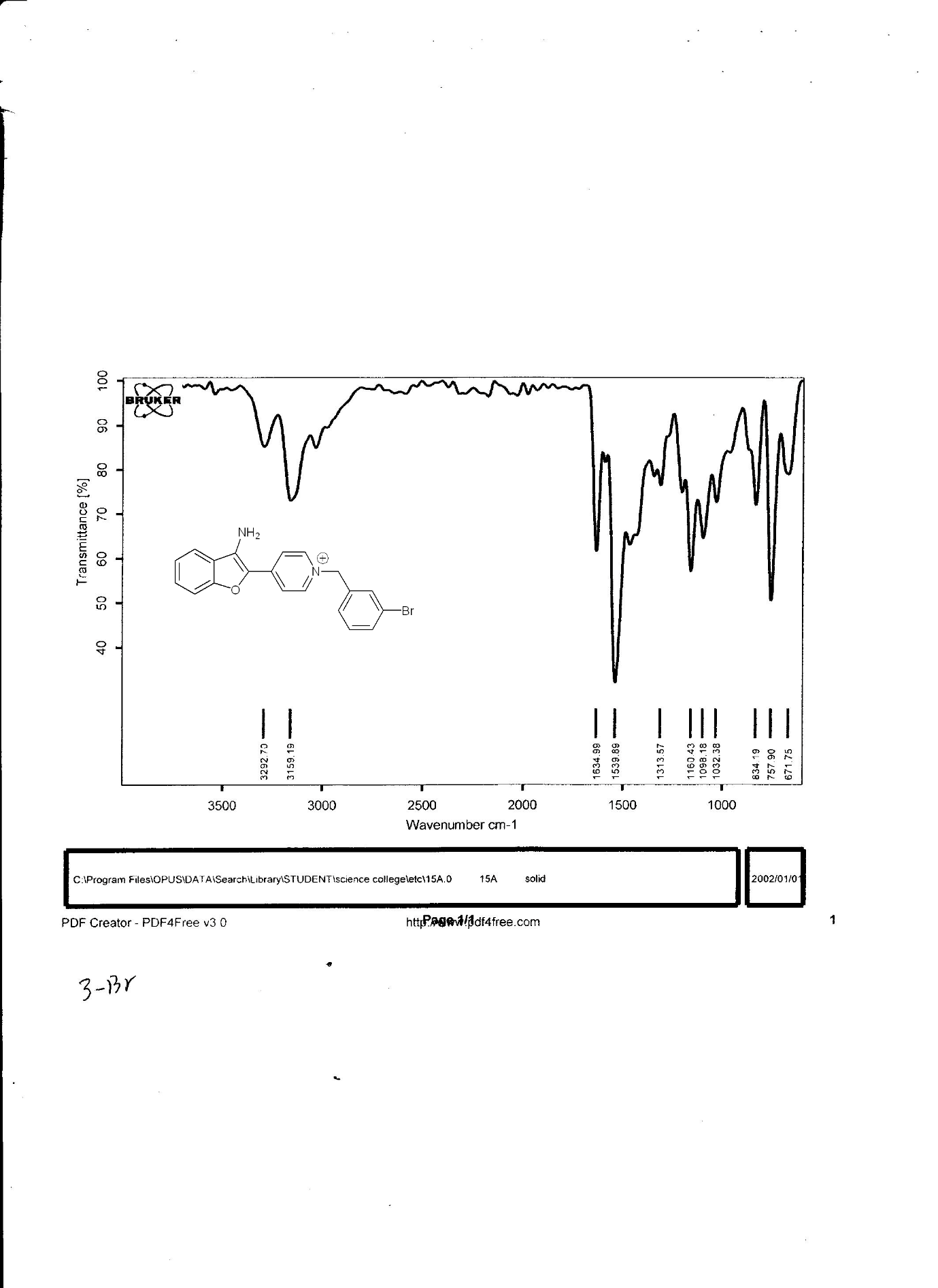


Figure S25. IR spectrum of compound **5m**


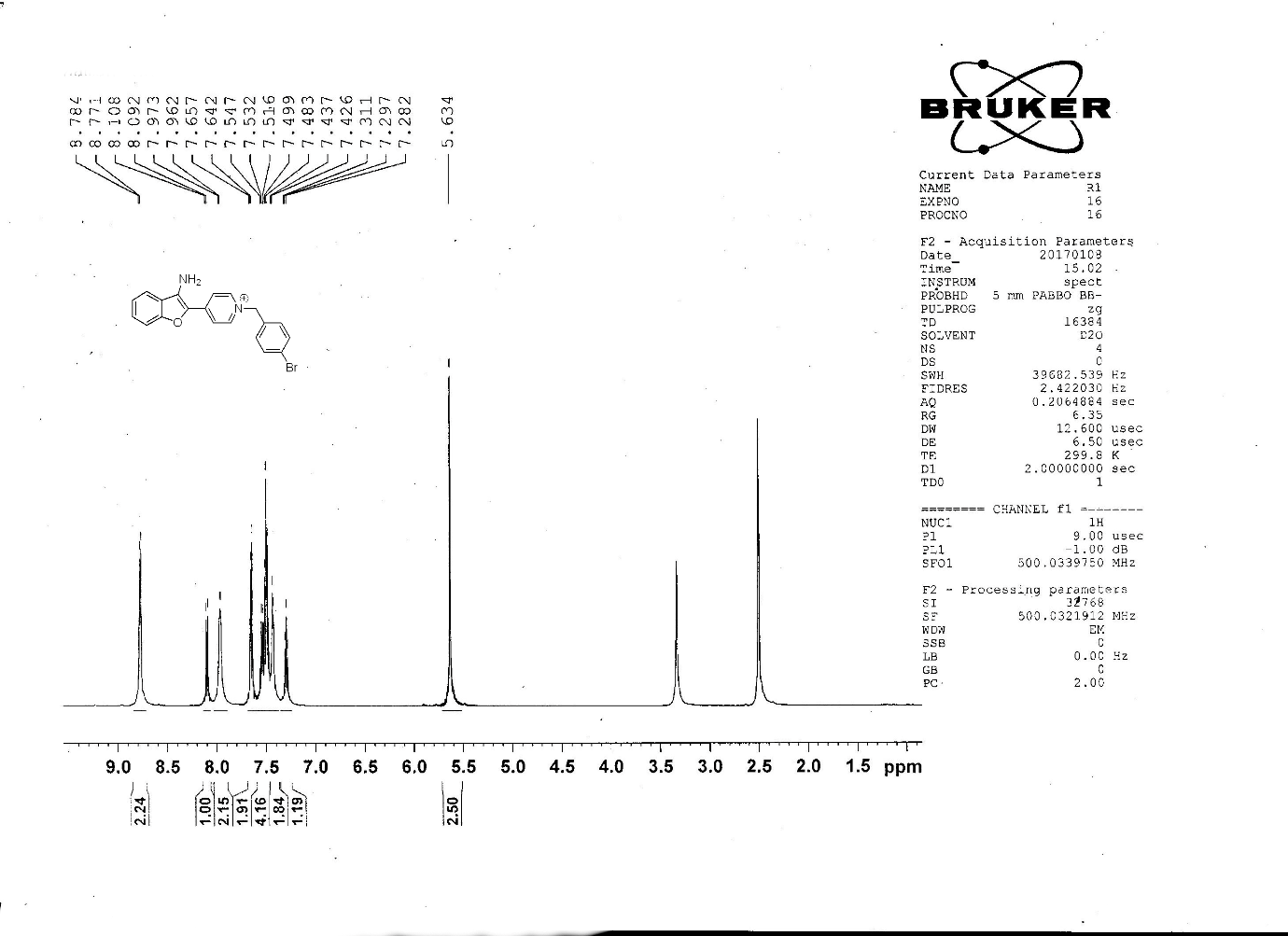


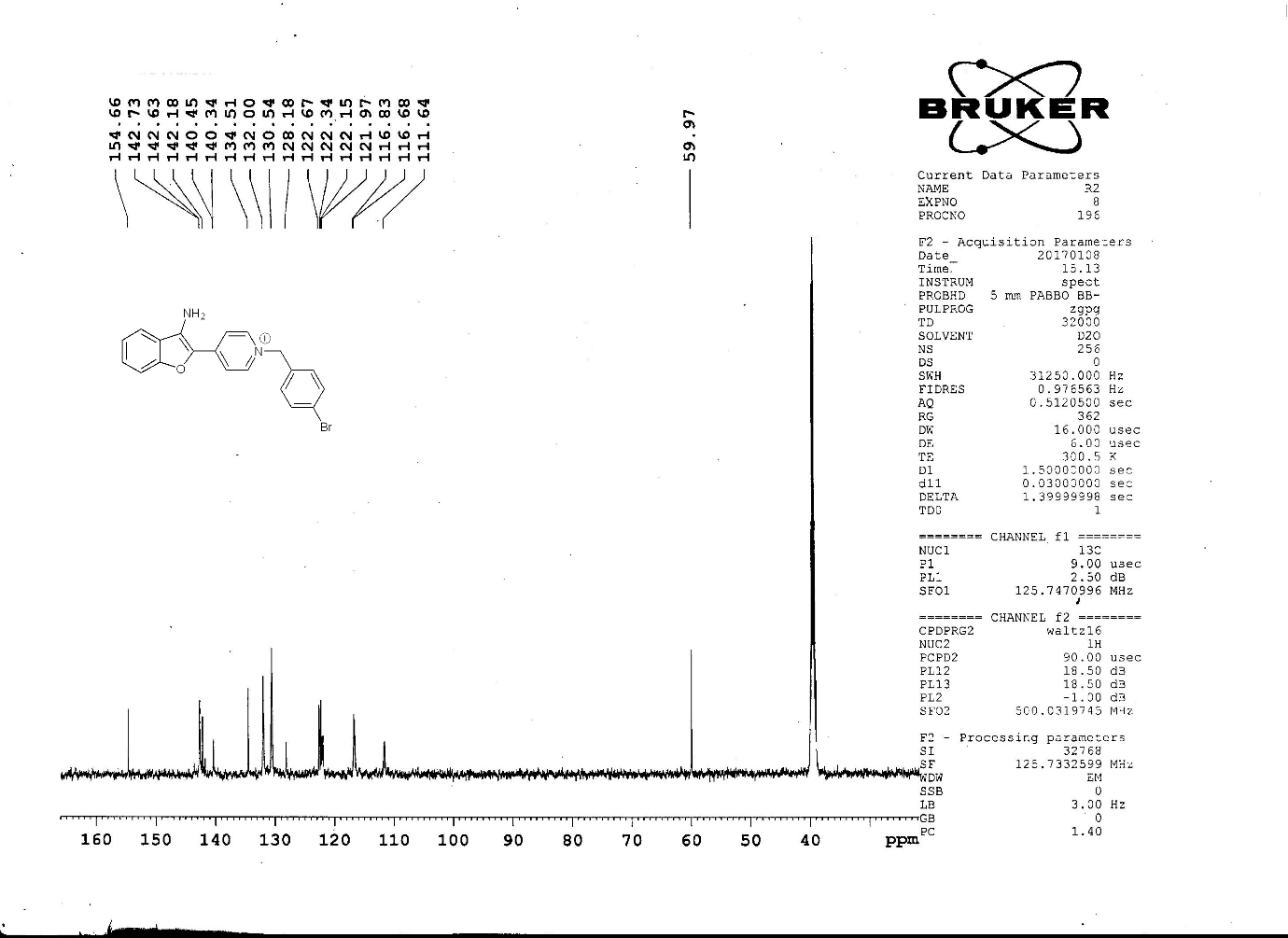


Figure S26. ^1^HNMR and ^13^CNMR spectra of compound **5n**

**
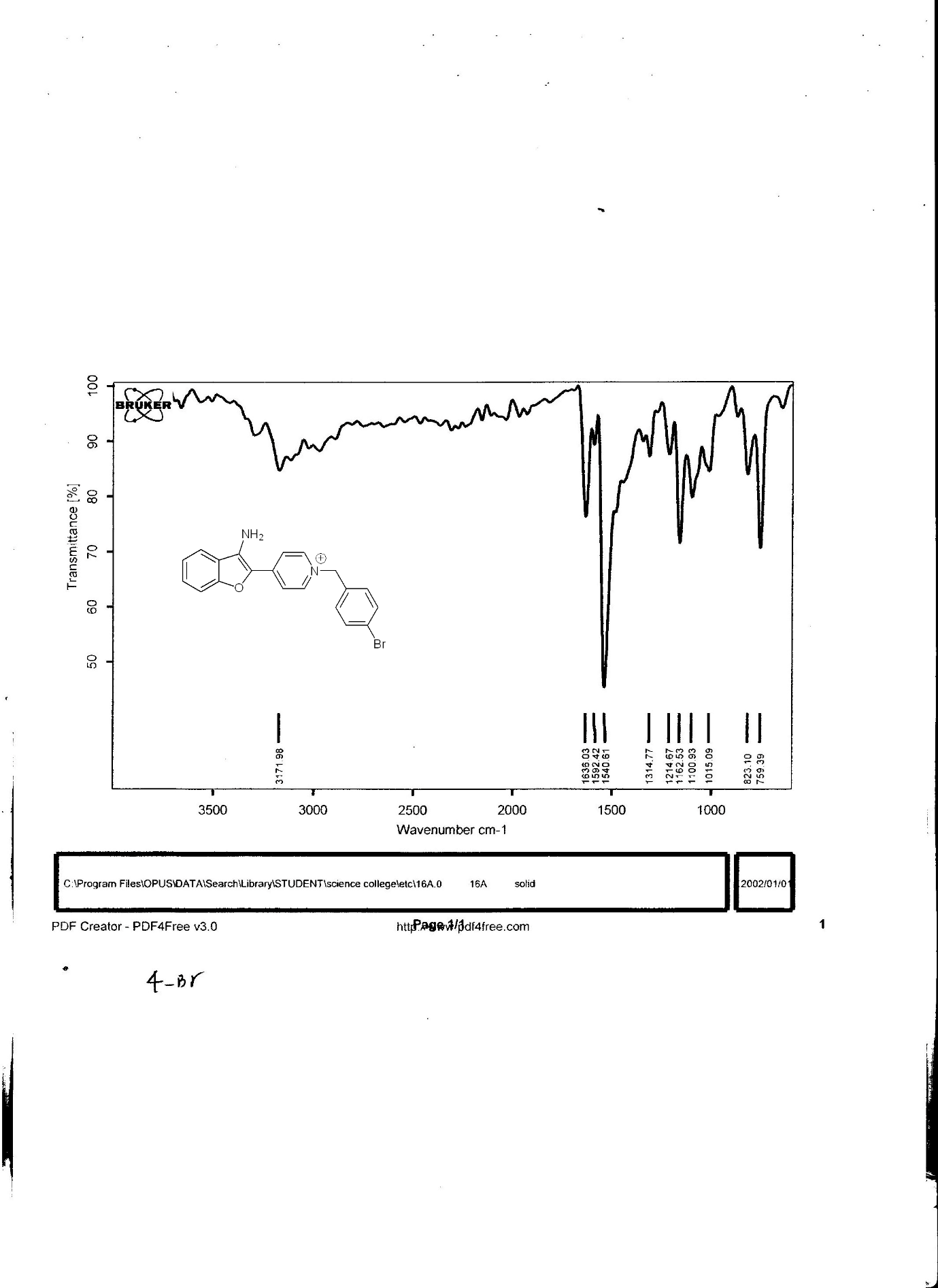
**

Figure S27. IR spectrum of compound **5n**

**
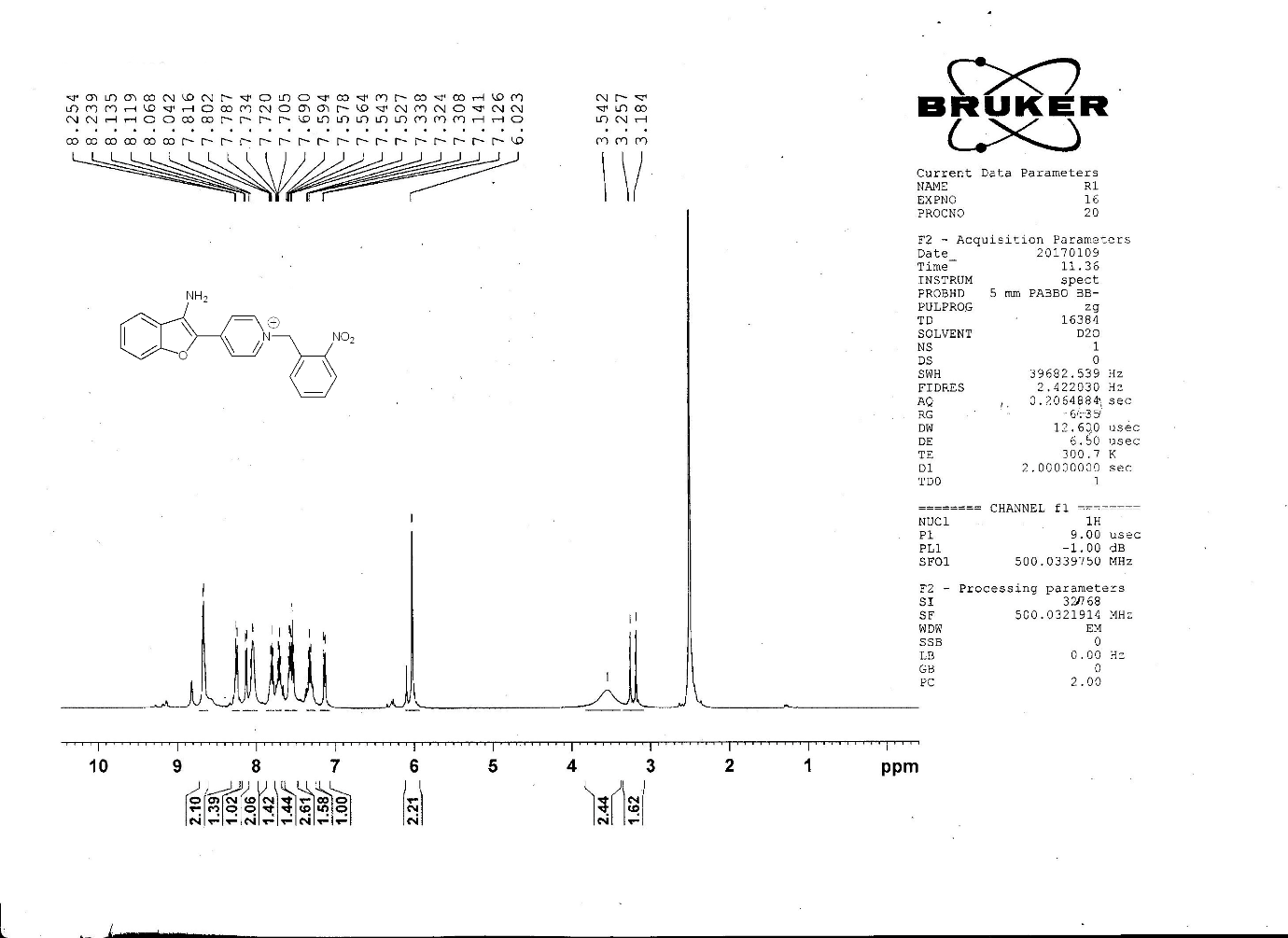

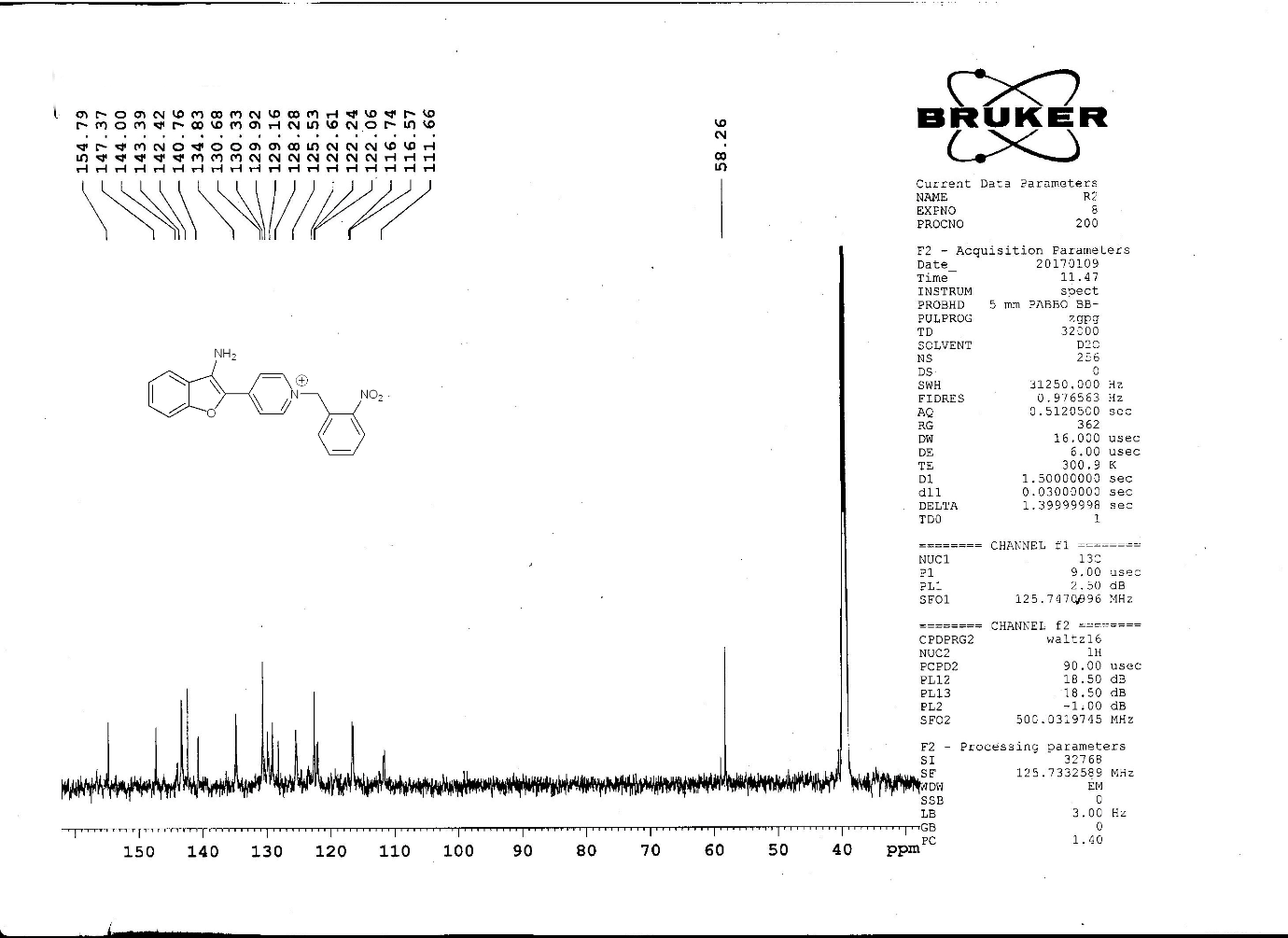
**

Figure S28. ^1^HNMR and ^13^CNMR spectra of compound **5o**

**
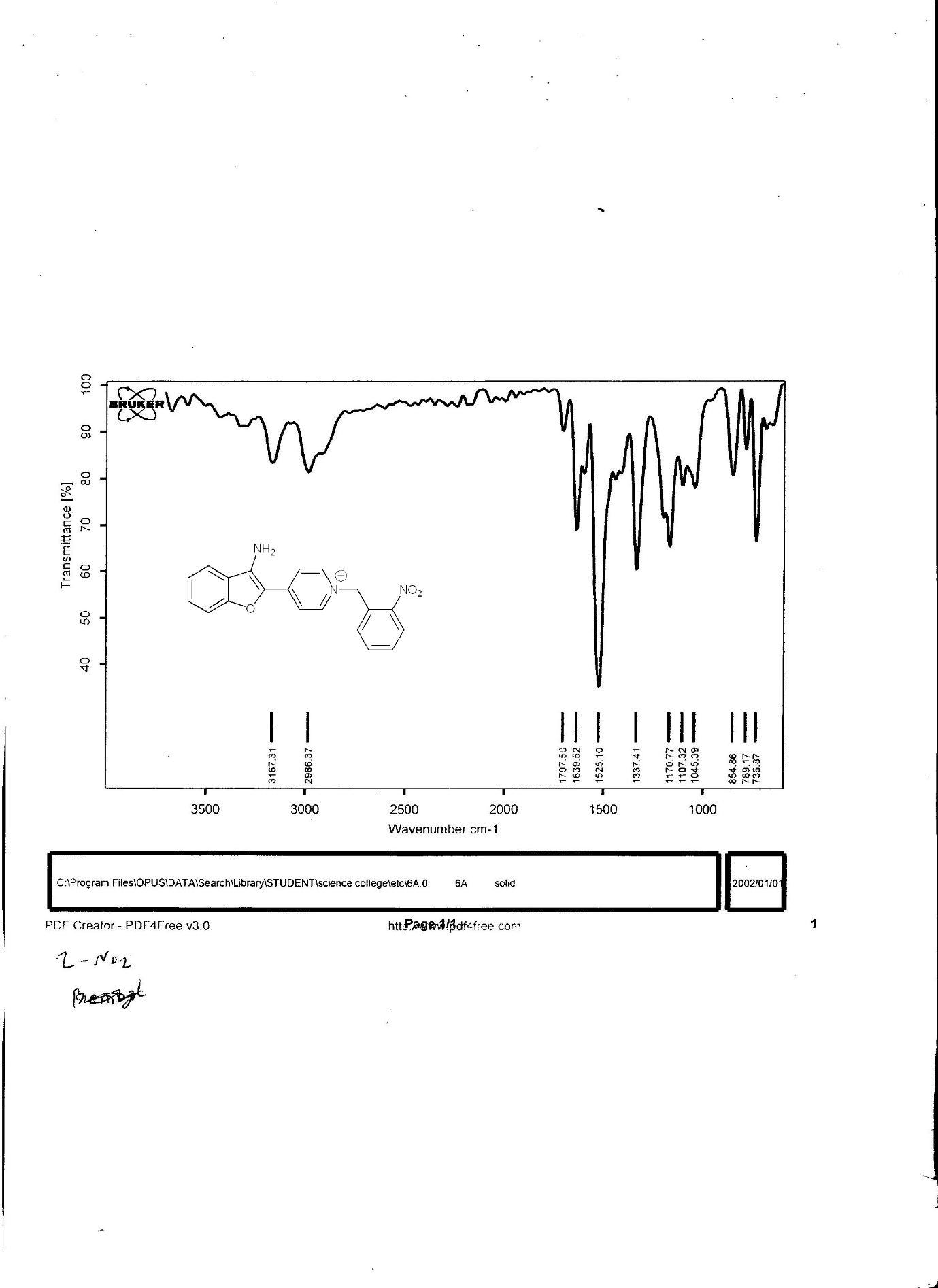
**

Figure S29. IR spectrum of compound **5o**


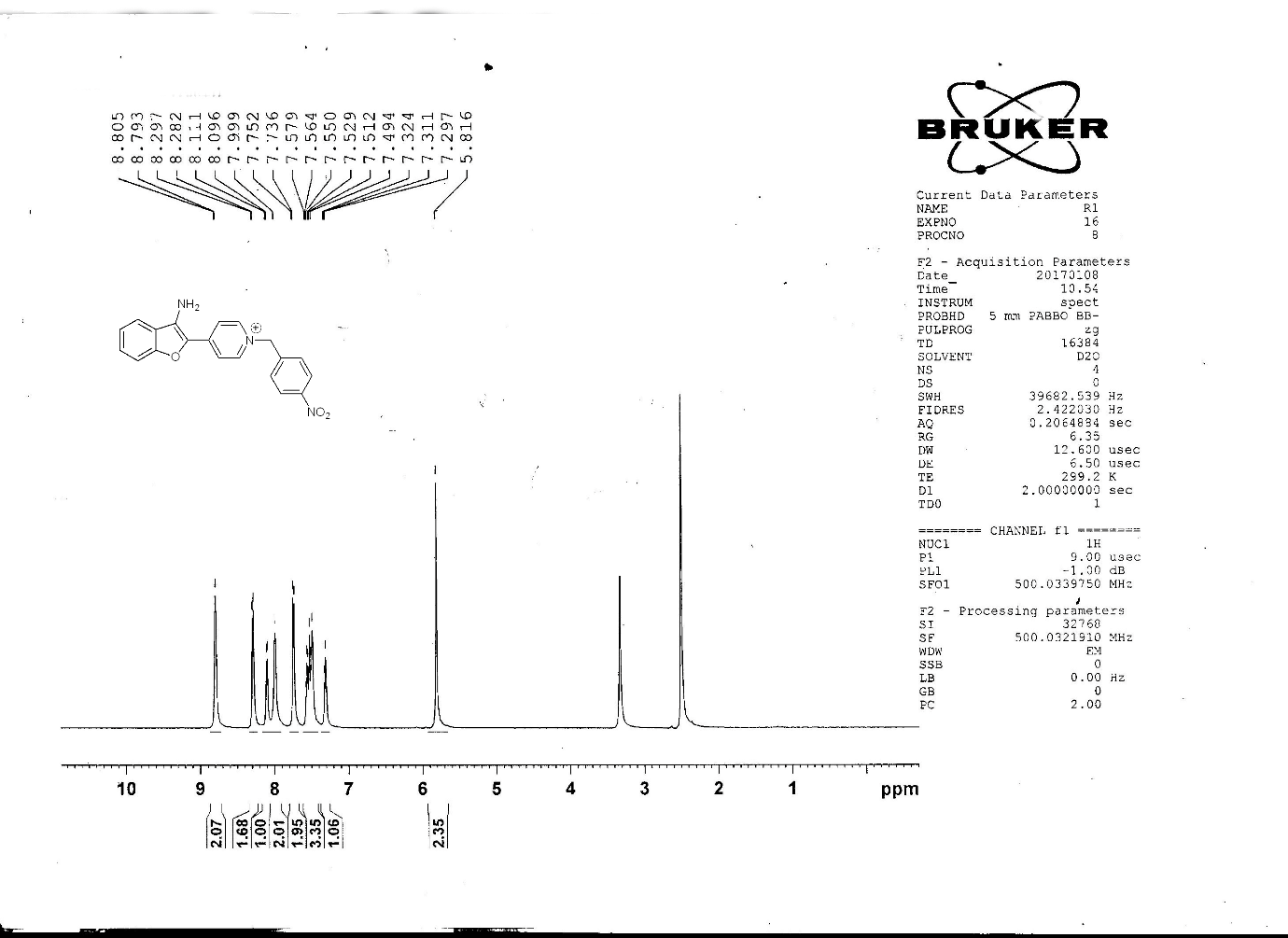


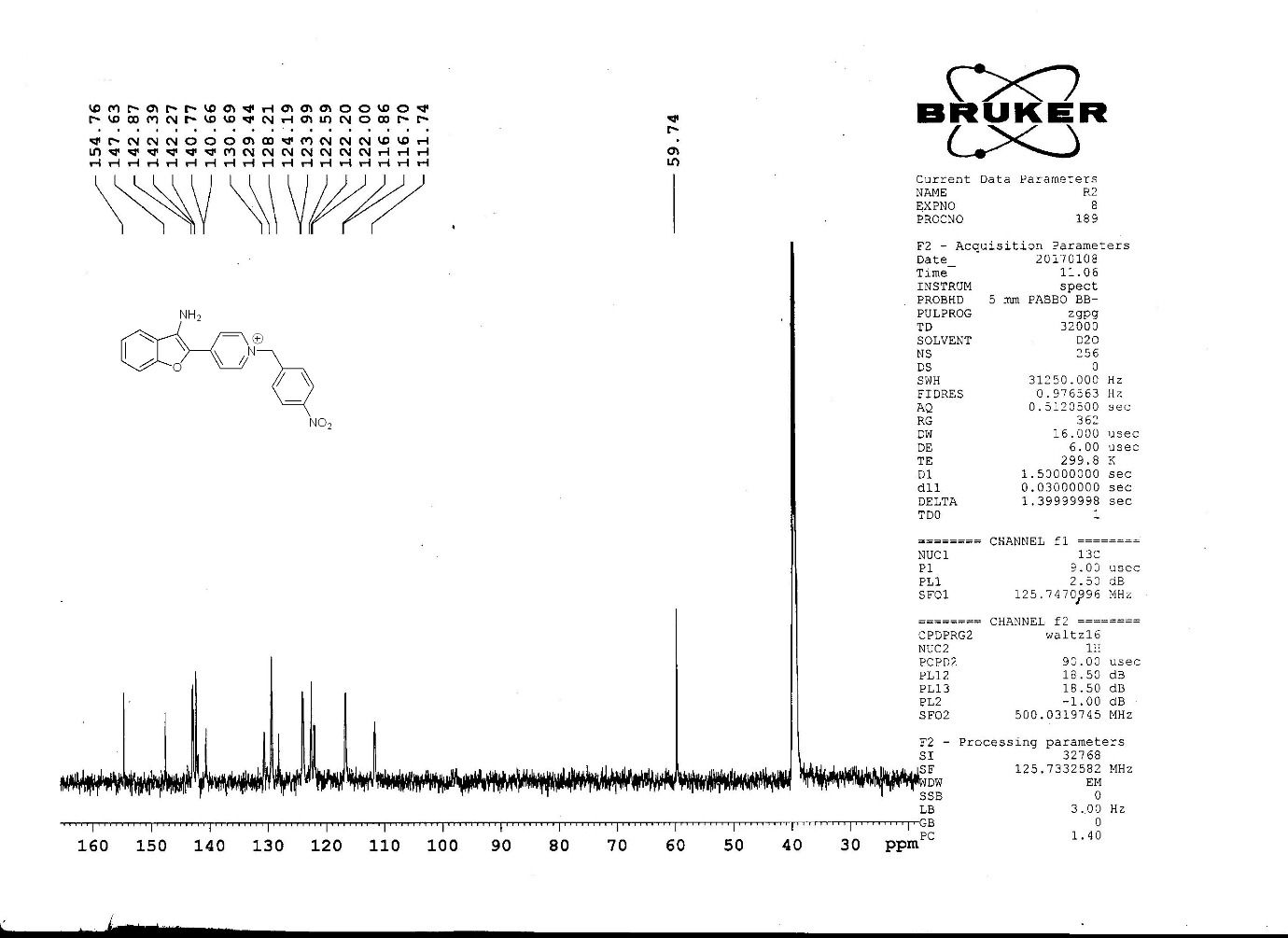


Figure S30. ^1^HNMR and ^13^CNMR spectra of compound **5p**

**
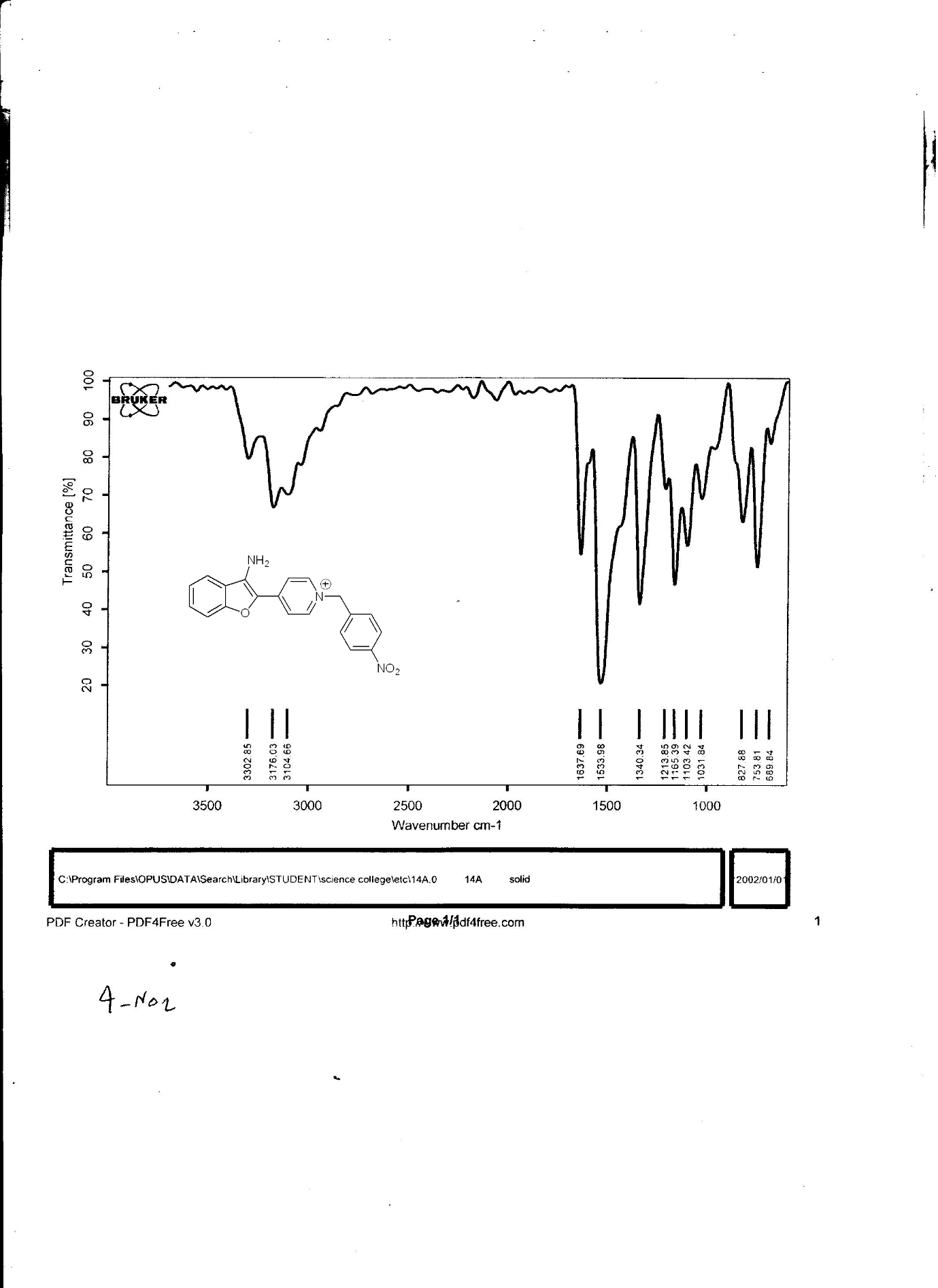
**

Figure S31. IR spectrum of compound **5p**
